# Selective ubiquitination of drug-like small molecules by the ubiquitin ligase HUWE1

**DOI:** 10.1038/s41467-025-63442-x

**Published:** 2025-09-02

**Authors:** Barbara Orth, Pavel Pohl, Florian Aust, Yanlong Ji, Ayshwarya Seenivasan, Olexandr Dybkov, Xiaojun Julia Liang, Lars Bock, Florian Leidner, Sophie Levantovsky, Patrick Schardey, Pascal Sander, Nathanael J. Disch, Masanja L. Trautz, Athanasia Mizi, Argyris Papantonis, Christof Lenz, Helmut Grubmüller, Wieland Steinchen, Christian Behrends, Henning Urlaub, Matthias Gehringer, Sonja Lorenz

**Affiliations:** 1 Max Planck Institute for Multidisciplinary Sciences, Research Group ‘Ubiquitin Signaling Specificity’, Göttingen, Germany; 2 Max Planck Institute for Multidisciplinary Sciences, Research Group ‘Bioanalytical Mass Spectrometry’, Göttingen, Germany; 3 Bioanalytics, Department of Clinical Chemistry, University Medical Center Göttingen, Göttingen, Germany; 4 Department for Medicinal Chemistry, Institute for Biomedical Engineering, Faculty of Medicine, Eberhard Karls University Tübingen, Tübingen, Germany; 5 Department of Pharmaceutical/Medicinal Chemistry, Institute of Pharmaceutical Sciences, Eberhard Karls University Tübingen, Tübingen, Germany; 6 Cluster of Excellence iFIT (EXC 2180) ‘Image-Guided & Functionally Instructed Tumor Therapies’, Eberhard Karls University Tübingen, Tübingen, Germany; 7 Max Planck Institute for Multidisciplinary Sciences, Department of Theoretical and Computational Biophysics, Göttingen, Germany; 8 Munich Cluster for Systems Neurology (SyNergy), Faculty of Medicine, Ludwig-Maximilians-University München, Munich, Germany; 9 Institute of Pathology, University Medical Center Göttingen, Göttingen, Germany; 10 Department of Chemistry, Philipps University Marburg, Marburg, Germany; 11 Center for Synthetic Microbiology, Philipps University Marburg, Marburg, Germany

**Keywords:** Enzyme mechanisms, Enzyme mechanisms

## Abstract

The ubiquitin system regulates eukaryotic physiology by modifying myriad substrate proteins. Substrate specificity and the assembly of ubiquitin signals are determined by ubiquitin ligases, some of which also modify non-protein biomolecules. Here we expand this substrate realm, revealing that the human ligase HUWE1 can target drug-like small molecules. We demonstrate that compounds previously reported as HUWE1 inhibitors present substrates of their target ligase. Compound ubiquitination is driven by the canonical catalytic cascade, linking ubiquitin to the compound’s primary amino group. In vitro, the modification is selectively catalyzed by HUWE1, allowing the compounds to compete with protein substrates. We establish cellular detection methods, confirming HUWE1 promotes — but does not exclusively drive — compound ubiquitination in cells. Converting the existing compounds into specific HUWE1 substrates or inhibitors thus requires enhanced specificity. More broadly, our findings open avenues for harnessing the ubiquitin system to transform exogenous small molecules into novel chemical modalities within cells.

## Abstract (web summary)

Ubiquitination is a versatile modification system in eukaryotic cells. Here, the authors unveil that the ubiquitin ligase HUWE1 can modify drug-like small-molecule substrates, beyond proteins. This discovery may be harnessed to develop specific tool substrates or inhibitors of HECT-type ligases.

## Introduction

The ubiquitin (Ub) system is a central regulator of eukaryotic homeostasis. Historically, Ub signals have been known to modify protein substrates, thereby governing virtually all aspects of protein-mediated cellular functions^1^. It has recently emerged that ubiquitination also targets non-proteinaceous matter, opening a new – and possibly vast – chapter in Ub biology^2,3^. For example, Ub can be transferred to carbohydrates^4–7^, nucleic acids^8,9^, ADP-ribose^10–14^, and metabolites^7,15–17^ in vitro. Moreover, cellular evidence of Ub-modified membrane phospholipids^18^, lipopolysaccharides^19^, and protein-linked ADP-ribose^12,20^ has been presented. Like classical protein ubiquitination, these atypical modifications are assembled by a sequence of E1 (Ub-activating enzyme), E2 (Ub-conjugating enzyme), and E3 (Ub ligase) activities, in which the latter typically determine substrate recognition. To achieve specificity, the E3 family is highly diversified, with over 600 members in the human proteome^21^. The few E3s thus-far identified to drive non-protein ubiquitination fall into different classes, including RING-type ligases of the DELTEX family^11–13^, the SCF^FBS2^-ARIH1 complex, the RBR-type HOIL-1^4–6^, and the non-canonical RNF213^19^ and MYCBP2^15^. In addition, *Legionella* effectors of the SidE-family were found to transfer Ub onto ADP-ribose and subsequently onto substrate proteins of the host via unconventional principles^2,22–25^. Here, we extend the substrate realm of non-protein ubiquitination by demonstrating that Ub ligases can selectively modify exogenous, drug-like molecules, both in vitro and in cells.

Our discovery originated, unexpectedly, from efforts to elucidate the mechanism of commercially available small-molecule inhibitors of human HUWE1. This large (482 kDa) E3 regulates DNA repair, transcription, and protein quality control, among other pathways, and is considered an interesting, yet unexploited cancer-therapeutic target^26^. As a member of the HECT (homologous to E6AP C-terminus) ligase family, HUWE1 employs a reactive cysteine in its C-terminal catalytic HECT domain to form a thioester-linked intermediate with Ub, before transferring it to a substrate. While HECT E3s should, in principle, be druggable by covalent targeting of the catalytic cysteine, this strategy has not yet been successfully implemented. Only few small-molecule HECT E3 inhibitors have been mechanistically characterized^27^, including electrophilic compounds modifying a non-catalytic cysteine in the HECT domain of NEDD4^28, 29^ and a non-covalent, allosteric inhibitor of the HECT domain of SMURF1^30^. The inhibition modes of other HECT E3 inhibitors have remained elusive, and their cellular specificities are not always well-established^27^. Among those understudied compounds, the HUWE1-directed BI8622 and BI8626 caught our attention, due to their drug-like chemical scaffolds, dose-dependent activities, and in vitro selectivity^31^. These inhibitors were identified in a high-throughput screen (HTS) of over 800,000 compounds, monitoring auto-ubiquitination of the isolated HECT domain of HUWE1 (HUWE1^HECT^) in reconstituted reactions. In this assay format, both compounds displayed low-micromolar IC_50_-values and selectivity for HUWE1^HECT^ over other purified HECT domains. While the inhibitors were found to stabilize individual HUWE1 substrates under certain conditions, such as MCL1 upon DNA damage induction, and to have selective cytotoxic effects in colorectal cancer cells, their pharmacokinetic properties do not allow for in vivo applications^31^. Moreover, the lack of understanding their inhibition mechanism has prohibited successful medicinal-chemistry optimization of the compounds toward enhanced specificity and potency.

In this study, we reveal that BI8622 and BI8626 constitute substrates of their target ligase and thus inhibit both HUWE1^HECT^ and the full-length ligase (HUWE1^FL^) in a substrate-competitive manner in vitro. We provide proof-of-principle evidence for HUWE1-mediated – but not specific – compound ubiquitination in the context of human cells. These findings imply that other ubiquitination enzymes have the capacity to modify the compounds with Ub and that a considerable increase in specificity would be required to convert them into specific substrates or inhibitors of HUWE1 for cellular use. Consistently, BI8626 elicits widespread proteomic effects and broadly reduces ubiquitination at many protein sites. Beyond targeting HUWE1, our demonstration that exogenous small molecules can present substrates of the Ub system highlights the interesting possibility to harness ubiquitination for creating novel chemical modalities in cells.

## Results

### HUWE1^HECT^ inhibitors are ubiquitinated at a critical amine

**Fig. 1:**
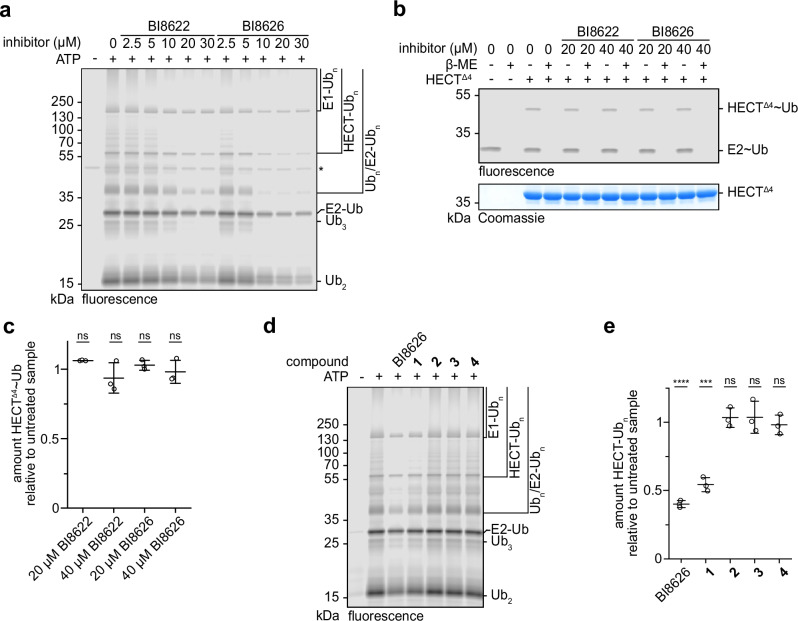
The HUWE1^HECT^ inhibitors require a primary amino group that is ubiquitinated. **a** Multi-turnover HUWE1^HECT^-driven ubiquitination reactions with varying concentrations of BI8622 and BI8626, respectively, followed by a fluorescent Ub tracer. ‘Ub_n_’ denotes ubiquitination at one or more sites of the indicated protein substrate. The asterisk marks an unspecifically labeled band. For protein input, see Supplementary Fig. 1a. **b** Single-turnover assay, monitoring Ub thioester transfer from E2 to HUWE1^HECT^ in the presence of the indicated concentrations of BI8622 and BI8626 with a fluorescent Ub tracer (top); protein input (bottom). Thioester-linked (‘~’) complexes are characterized by their sensitivity to reducing agent (β-ME). Note that a truncated HECT domain variant (‘HECT^Δ4^’) is used to stabilize the HUWE1^HECT^~Ub product^32^. **c** Quantification of assays as shown in (**b**). For each experiment, the amount of the HUWE1^HECT^~Ub product was normalized to inhibitor-free (‘untreated’) conditions. Statistical analyses, using an unpaired, two-sided *t*-test; ns = not significant (compared to untreated conditions; *p *> 0.05). Data are represented as mean ± SD (*n* = 3 biological replicates). **d** Multi-turnover HUWE1^HECT^-driven ubiquitination reactions with 20 μM of BI8626 or the indicated derivatives (Table 1), followed by a fluorescent Ub tracer; for protein input, see Supplementary Fig. 5. **e** Quantification of assays as shown in (**d**). For each experiment, the amount of HUWE1^HECT^ autoubiquitination was normalized to inhibitor-free conditions (‘untreated’). Data are represented as mean ± SD (*n* = 3 biological replicates). Statistical analyses with an unpaired, two-sided *t*-test, relative to untreated conditions, ****p = 0.0000023; ***p = 0.00011. Source data are provided as a Source Data file.

Only few small-molecule HECT E3 inhibitors have been reported and our understanding of their inhibition mechanisms and specificities is still limited^27,30,29^. To decipher how BI8626 and BI8622 inhibit HUWE1^HECT^, we monitored multi-turnover ubiquitination reactions in solution, containing E1 (UBA1), E2 (UBE2L3), HUWE1^HECT^, Ub, and ATP, with a fluorescent Ub tracer. In contrast to the original HTS, which exclusively followed the autoubiquitination of immobilized HUWE1^HECT^^[ 31^, this assay format allowed for all reaction products to be visualized by SDS-PAGE. We found, consistently, that the compounds inhibit ubiquitination dose-dependently in the low-micromolar concentration range, with BI8626 being more potent than BI8622 (Fig. 1a; Supplementary Fig. 1a–c). The inhibitors suppress E3 autoubiquitination, E2 ubiquitination and free Ub chain formation, indicating broad inhibition of HUWE1^HECT^ catalysis, independent of the Ub-accepting protein. Similar effects were observed with an alternative E2, UBE2D3, suggesting the inhibition is not determined by the E2 (Supplementary Fig. 1d, e). In the following, we employed UBE2L3, as it is specific for catalytic cysteine-driven E3s^17^ and more reactive with HUWE1 than UBE2D3^15^. Interestingly, single-turnover assays demonstrate that the two inhibitors do not obstruct Ub transfer from the E2 to HUWE1^HECT^ (Fig. 1b, c); nor do they perturb the preceding Ub transfer from the E1 to the E2 (Supplementary Fig. 1f, g). This implies that the compounds inhibit HUWE1^HECT^ after the formation of a thioester-linked (‘~’) intermediate with Ub, specifically interfering with the second step of HECT E3 catalysis.

We thus reconstituted a stable, vinylthioether-linked proxy of HUWE1^HECT^~Ub^32^ and analyzed its interactions with BI8626 and BI8622, respectively, by differential scanning fluorimetry (DSF) (Supplementary Fig. 2a–d). Moreover, ITC was performed with the more potent BI8626 (Supplementary Fig. 2e). Surprisingly, no interactions between HUWE1^HECT^-Ub and the inhibitors could be detected; analogous studies of *apo* HUWE1^HECT^ were also negative (Supplementary Fig. 2f–j). Likewise, hydrogen-deuterium exchange-mass spectrometry (HDX-MS) analyses of the HUWE1^HECT^~Ub proxy and HUWE1^HECT^, respectively, at a 10-fold molar excess of either inhibitor did not provide evidence of binding (Supplementary Figs. 3, 4), and X-ray crystallographic efforts failed to yield protein structures in complex with the compounds (data not shown). Together, this suggests that the interactions of the inhibitors with HUWE1^HECT^ disfavor detection by any of the techniques and conditions used here or that additional components of the ubiquitination cascade are required to stabilize them.

To derive a structure-activity relationship for BI8626, we synthesized several derivatives (Table 1), initially focusing on the primary amino group at the lower benzyl moiety, as it is common to both inhibitors. Shifting this group from the *meta* to the *para* position of the benzyl ring (derivative **1**) retained HUWE1^HECT^ inhibition (Fig. 1d, e; Supplementary Fig. 5). In contrast, removal of the amino group or substitution by a secondary or tertiary amine caused a loss of inhibition (derivatives **2**–**4**). The requirement of a primary amine sparked the idea that the compounds may be ubiquitinated as part of their inhibition mechanism. To test this hypothesis, we separated ubiquitination reactions by SDS-PAGE and excised the Ub-containing band at ~9 kDa for MS/MS analyses. LysC protease digestion of Ub yields C-terminal peptides with monoisotopic masses of 1449.84 Da (no missed cleavage) and 3210.71 Da (one missed cleavage), which were expected to be readily detectable in both unmodified and compound-linked forms (+408.21 Da for BI8622; +422.23 Da for BI8626). Indeed, we identified the modified peptides with BI8622 and BI8626, respectively, following incubation of the ubiquitination machinery with ATP (Table 2; Supplementary Figs. 6, 7). The observed fragment ions confirm sequence coverage and site-specific compound modification of the Ub C-terminus. Analogous modifications were detected for the primary amine-containing derivative **1** (Table 2; Supplementary Fig. 8). These data establish that drug-like small molecules can be ubiquitinated by HUWE1^HECT^ and suggest that this capacity may be linked to the inhibition mode of the compounds analyzed here.

**Table 1:** Overview of compound derivatives used in this study

**Table 2:** MS/MS analyses of HUWE1^HECT^-driven compound ubiquitination

compound	-	-	BI8622	BI8626	1
ATP	-	+	+	+	+
ESTLHLVLRLRGG-**BI8622**	X	X	yes	X	X
QLEDGRTLSDYNIQKESTLHLVLRLRGG-**BI8622**	X	X	yes	X	X
ESTLHLVLRLRGG-**BI8626**	X	X	X	yes	X
QLEDGRTLSDYNIQKESTLHLVLRLRGG-**BI8626**	X	X	X	yes	X
ESTLHLVLRLRGG-**1**	X	X	X	X	yes
QLEDGRTLSDYNIQKESTLHLVLRLRGG-**1**	X	X	X	X	yes

Table summarizing LC-MS/MS analyses of HUWE1^HECT^-driven multi-turnover ubiquitination reactions, containing the indicated compounds. The identification of the specified C-terminal Ub-derived peptides, resulting from LysC protease digestions with no or one missed cleavage site, respectively, is indicated by 'yes'. For the corresponding, annotated MS/MS spectra, see Supplementary Figs. 6–8.

### The inhibitor ubiquitination is driven by HUWE1^HECT^

**Fig. 2:**
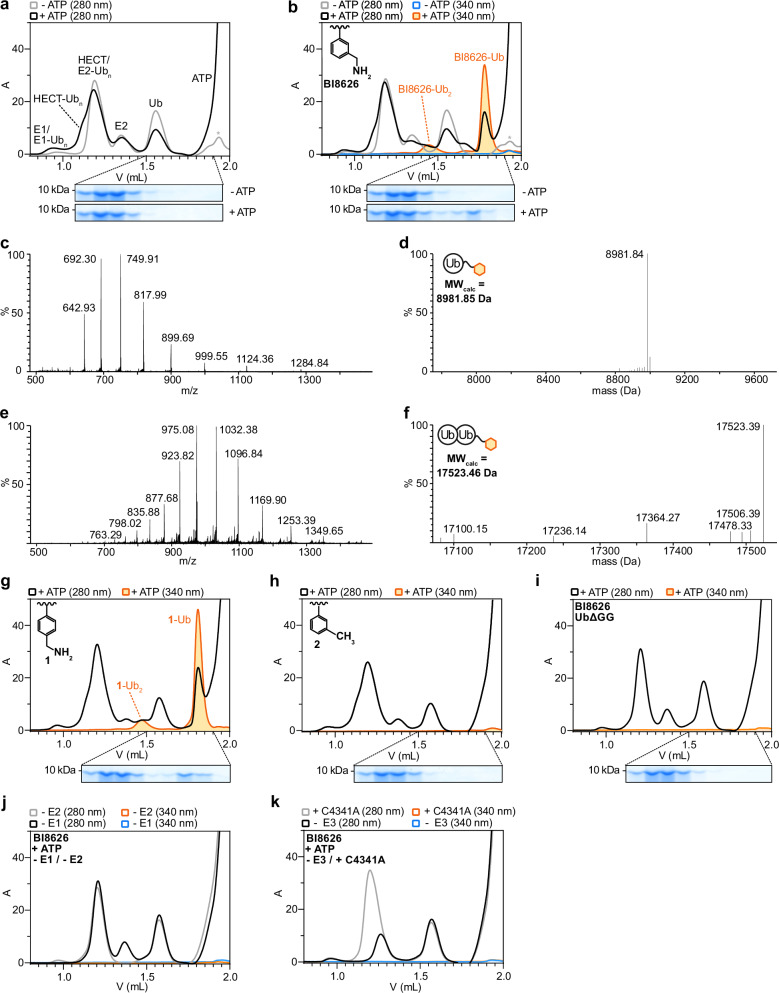
BI8626 ubiquitination requires the catalytic cascade, including HUWE1^HECT^. **a** SEC-based fractionation of HUWE1^HECT^-driven ubiquitination reactions ± ATP, without inhibitor (top). The assignment of the elution peaks is based on SDS-PAGE analyses, as shown in Supplementary Fig. 9a, b. The asterisk marks buffer components. SDS-PAGE analyses of the indicated elution fractions, focusing on the Ub-sized bands (bottom). **b** SEC-based fractionation of HUWE1^HECT^-driven ubiquitination reactions ± ATP in the presence of 20 μM BI8626 (top). SDS-PAGE analyses of the indicated elution fractions, focusing on the Ub-sized bands (bottom). Note that additional, delayed Ub-containing fractions are observed in the presence of ATP, which are not seen in inhibitor-free conditions. For the full SDS-PAGE, see Supplementary Fig. 9d. The lower benzyl moiety of BI8626, including the critical primary amino group is shown. Intact protein MS (**c**) and deconvoluted neutral MW spectrum (**d**) of the major BI8626-containing elution peak (orange; V ~ 1.8 mL) from (**b**). The calculated molecular weight (MW) of BI8626-Ub is specified. Intact protein MS (**e**) and deconvoluted neutral MW spectrum (**f**) of the minor BI8626-containing elution peak (orange; V ~ 1.4 mL) from (**b**). **g** SEC-based fractionation (top) and SDS-PAGE analysis (bottom) of a HUWE1^HECT^-driven ubiquitination reaction, containing 20 μM derivative **1**, analogous to (**b**). The lower benzyl moiety of derivative **1**, including the critical amino group is shown. **h** SEC-based fractionation (top) and SDS-PAGE analysis (bottom) of a HUWE1^HECT^-driven ubiquitination reaction, containing 20 μM derivative **2**, analogous to (**b**). The lower benzyl moiety of derivative **2** that does not contain a primary amino group is shown. **i** SEC-based fractionation (top) and SDS-PAGE analysis (bottom) of a HUWE1^HECT^-driven ubiquitination reaction, containing 20 μM BI8626, analogous to (**b**), but employing UbΔGG instead of WT Ub. **j** SEC-based fractionation of two independent HUWE1^HECT^-driven ubiquitination reactions, containing 20 μM BI8626, analogous to (**b**), but devoid of the E1 or E2. **k** SEC-based fractionation (top) (bottom) of two independent ubiquitination reactions, containing 20 μM BI8626, analogous to (**b**), but devoid of HUWE1^HECT^ or employing a catalytically inactive HUWE1^HECT^ variant (C4341A) instead of the WT. Source data are provided as a Source Data file.

To understand how BI8622 and BI8626 are ubiquitinated, we fractionated ubiquitination reactions by size-exclusion chromatography (SEC) (Fig. 2a; Supplementary Fig. 9a, b), exploiting the fact that the modified compounds elute later than free Ub (presumably due to hydrophobic interactions between the compounds and the column matrix) and that the inhibitors absorb in a UV wavelength-range, in which the protein components do not (Supplementary Fig. 9c). This allowed us to distinguish inhibitor-containing from protein-only elution peaks based on absorbance, monitoring λ = 280 nm for proteins, 340 nm for BI8626, and 320 nm for BI8622. In the following, we present data for BI8626 and BI8622 as main and Supplementary figures, respectively, as they are qualitatively similar. In the presence of ATP, inhibitor absorbance coincided with a major protein peak (Fig. 2b; Supplementary Fig. 9d–f) that was assigned to the respective monoubiquitinated compound by intact protein MS (Fig. 2c, d; Supplementary Fig. 9g, h). A fraction of the conjugate was further converted into a diubiquitinated form, showing that the compounds can provide substrates for Ub chain formation (Fig. 2b, e, f; Supplementary Fig. 9e, i, j). Analogous to BI8626 and BI8622, the primary amine-containing derivative **1** was efficiently ubiquitinated (Fig. 2g), but not the derivative **2**, which is devoid of an amino group at the lower benzyl substituent (Fig. 2h). No compound ubiquitination occurred with a Ub variant lacking the C-terminal di-glycine motif (‘UbΔGG’, residues 1–74) (Fig. 2i; Supplementary Fig. 10a). Together, these results establish that the small-molecule ubiquitination requires the Ub C-terminus and a primary amino group on the compounds, like canonical protein ubiquitination.

In line with this, the inhibitor ubiquitination relies on the entire enzymatic cascade: reactions lacking the E1 or E2 did not promote compound modification (Fig. 2j, Supplementary Fig. 10b), nor did reactions with a catalytically inactive HUWE1^HECT^ variant (C4341A) (Fig. 2k; Supplementary Fig. 10c). This demonstrates that the small-molecule inhibitors are ubiquitinated by the catalytic Cys-dependent activity of HUWE1^HECT^, providing substrates of their target ligase.

To visualize how BI8626 may nucleophilically attack the HUWE1^HECT^-linked donor Ub, we performed 100 2-μs molecular dynamics (MD) simulations with the compound and a crystal structure of a HUWE1^HECT^-donor Ub complex we had previously determined^32^. To accurately describe the dynamics of the ligand, its force field parameters were optimized to reproduce ab-initio calculation (Supplementary Fig. 11a, b). Filtering of the results for compound orientations compatible with a nucleophilic attack of the primary amino-nitrogen atom onto the C-terminal carbon atom of the donor Ub reveals four enriched clusters of poses (Supplementary Fig. 11c–f). Interestingly, top-scoring AlphaFold3 (AF3)-based predictions coincide with those conformations, when restraining the primary amino group of BI8626 proximal to the C-terminal carbonyl of Ub (Supplementary Fig. 11e–g). While the models differ markedly in the position of the compound, they collectively predict contacts of BI8626 with regions in both HECT lobes that are critical for aminolysis, specifically the active site and the C-terminal extension of the C-lobe as well as the ligase-organizing loop (LOL^33–35^) and adjacent β1/ β2-sheet of the N-lobe^35^.

### The compounds are efficient, selective HUWE1^HECT^ substrates

**Fig. 3:**
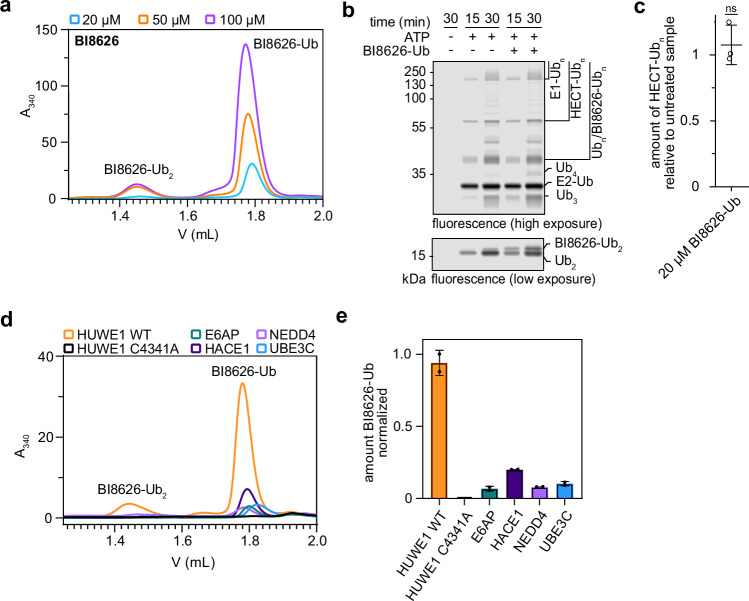
BI8626 is an efficient and selective substrate of HUWE1^HECT^. **a** SEC-based analyses of HUWE1^HECT^-driven ubiquitination reactions, containing the indicated BI8626 concentrations, monitoring the mono- and diubiquitinated BI8626 by absorbance at 340 nm. **b** Multi-turnover HUWE1^HECT^-driven ubiquitination reactions ±20 μM purified mono-ubiquitinated BI8626 (BI8626-Ub), followed by a fluorescent Ub tracer. The bottom part of the gel is shown at a different exposure to allow for diUb and diUb-modified BI8626 to be distinguished. For protein input, see Supplementary Fig. 12b. **c** Quantification of assays as shown in (**b**). For each experiment, the amount of HUWE1^HECT^ autoubiquitination at 30 minutes was normalized to that under inhibitor-free (‘untreated’) conditions. Data are represented as mean ± SD (*n* = 3 biological replicates). Statistical analysis using un unpaired, two-sided *t*-test (ns (not significant): *p* $\ge$ 0.05). **d** SEC-based analyses of BI8626 ubiquitination, driven by the indicated HECT domains, monitoring the mono- and diubiquitinated BI8626 by absorbance at 340 nm. **e** Quantification of BI8626 monoubiquitination by the indicated HECT domains from SEC analyses as shown in (**d**). The signal produced by HUWE1^HECT^ was normalized to 1. Data are represented as mean ± SD (*n* = 2 biological replicates). Source data are provided as a Source Data file.

By providing substrates for ubiquitination, BI8626 and BI8622 may interfere with the activity of HUWE1^HECT^ toward proteins either in a substrate-competitive manner or via product inhibition. Dose-response studies reveal that HUWE1^HECT^ readily ubiquitinates high compound concentrations, where its isopeptide bond formation activity toward acceptor proteins is fully inhibited (Fig. 3a; Supplementary Fig. 12a). This suggests that the ubiquitinated product (‘BI8626-Ub’) does not generally obstruct HUWE1^HECT^ catalysis, but rather that small-molecule and protein substrates may compete with each other for the ligase. In line with this notion, HUWE1^HECT^ remains active upon addition of SEC-purified BI8626-Ub, arguing against product inhibition (Fig. 3b, c; Supplementary Fig. 12b).

We next compared the activity of HUWE1^HECT^ toward BI8626 and BI8622 with that of other HECT domains, including E6AP (UBE3A), HACE1, NEDD4, and UBE3C (Fig. 3d, e; Supplementary Fig. 12c, d). Previous in vitro studies had reported the compound-dependent inhibition to be selective for HUWE1^HECT^, with IC_50_-values of 0.9 (BI8626) and 3.1 μM (BI8622), compared to values of >50 μM for other HECT domains^31^. Our quantitative analyses of inhibitor ubiquitination mirror this trend, demonstrating substantially higher activity for HUWE1^HECT^ than for the other HECT domains. The correlation between selective inhibition and selective compound ubiquitination further supports a substrate-competitive inhibition model. Notably, however, certain compound-directed activities were detected for all tested HECT domains, revealing a general propensity of the domains to ubiquitinate these drug-like molecules.

### The compounds compete with protein substrates for HUWE1^FL^

**Fig. 4:**
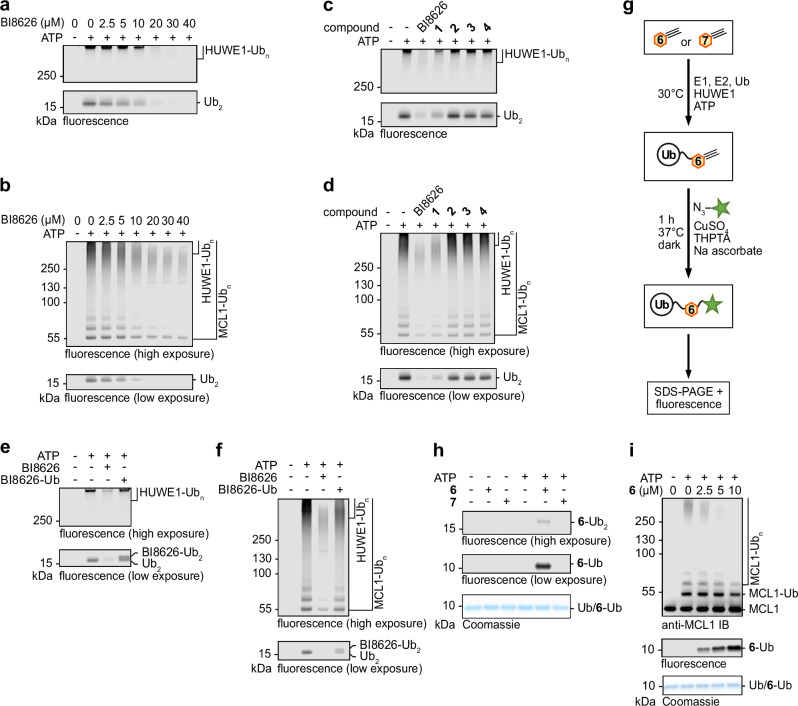
HUWE1^FL^ promotes compound ubiquitination in a manner that competes with protein substrate modification. **a** Multi-turnover HUWE1^FL^-driven ubiquitination reactions in the presence of the indicated BI8626 concentrations, without protein substrate, followed by a fluorescent Ub tracer; for protein input, see Supplementary Fig. 13a. **b** Multi-turnover HUWE1^FL^-driven MCL1 ubiquitination reactions in the presence of the indicated BI8626 concentrations, followed by a fluorescent Ub tracer; for protein input, see Supplementary Fig. 13f. **c** Multi-turnover HUWE1^FL^-driven ubiquitination reactions in the presence of the indicated BI8626 derivatives (20 μM), without protein substrate, followed by a fluorescent Ub tracer; for protein input, see Supplementary Fig. 14e. **d** Multi-turnover HUWE1^FL^-driven MCL1 ubiquitination reactions in the presence of the indicated BI8626 derivatives, followed by a fluorescent Ub tracer; for protein input, see Supplementary Fig. 14f. **e** Multi-turnover HUWE1^FL^ ubiquitination reactions in the presence of 20 μM BI8626 or purified BI8626-Ub, without protein substrate, followed by a fluorescent Ub tracer; for protein input, see Supplementary Fig. 18a. **f** Multi-turnover HUWE1^FL^-driven MCL1 ubiquitination reactions in the presence of 20 μM BI8626 or purified BI8626-Ub, followed by a fluorescent Ub tracer; for protein input, see Supplementary Fig. 18b. **g** Cartoon of our click chemistry-based strategy for detecting inhibitor (derivative **6**; Table 1) ubiquitination in vitro. An azide-linked fluorophore is symbolized by a star. **h** Proof-of-principle experiment for the detection of HUWE1^FL^-driven derivative **6** ubiquitination, according to (**g**). Note that at high exposure (top), a small amount of Ub chain formation on the compound is observed. **i** Multi-turnover HUWE1^FL^-driven ubiquitination reactions in the presence of varying concentrations of derivative **6** and MCL1, followed by fluorescence (for compound ubiquitination) and IB (for MCL1 ubiquitination), respectively. The Ub signal is shown by Coomassie staining. Source data are provided as a Source Data file.

As previous in vitro studies had validated the inhibitors only in the context of isolated HECT domains^31^, we next expanded our analyses to the 482-kDa HUWE1^FL^. BI8626 and BI8622 inhibit the protein ubiquitination activity of the ligase in a dose-dependent manner, as seen in E3 autoubiquitination, free Ub chain formation (Fig. 4a, Supplementary Fig. 13a–e) and substrate-dependent reactions, using MCL1 as a physiological HUWE1 model substrate^36^ (Fig. 4b, Supplementary Fig. 13f–j). Studies with two alternative substrates, histone 2B^37^ and FOXP3^38^ show similar effects, indicating that the compound-mediated HUWE1 inhibition is not MCL1-specific (Supplementary Fig. 14a–d). As for HUWE1^HECT^, the full-length ligase was also inhibited by the primary amine-containing derivative **1**, but not the primary amine-deprived derivatives **2,** **3**, and **4** (Fig. 4c, d, Supplementary Fig. 14e, f). Consistently, MS/MS analyses of reconstituted ubiquitination reactions demonstrated HUWE1^FL^-driven ubiquitination of BI8622, BI8626, and the primary amine-containing derivative **1** in an ATP-dependent manner, analogously to HUWE1^HECT^ (Table 3; Supplementary Figs. 15–17). Compared to BI8626, the purified BI8626-Ub product conferred markedly less inhibition of HUWE1^FL^, in line with the observation that the free compound, but not its ubiquitinated form efficiently inhibited HUWE1^HECT^ (Fig. 4e, f; Supplementary Fig. 18a, b).

**Table 3:** MS/MS analyses of HUWE1^FL^-driven compound ubiquitination

compound	BI8622	BI8626	1	BI8622	BI8626	1
ATP	-	-	-	+	+	+
**BI8622**-Ub	X	X	X	yes	X	X
**BI8626**-Ub	X	X	X	X	yes	X
**1-**Ub	X	X	X	X	X	yes

Table summarizing the results of targeted MS/MS analyses of reconstituted HUWE1^FL^-driven ubiquitination reactions, containing the indicated compounds ± ATP. 'yes' indicates detection of compound-modified tryptic C-terminal ubiquitin peptides characteristic of the conjugated products listed in the left column. For the corresponding assigned spectra, see Supplementary Figs. 15–17.

We next developed a click-chemistry strategy for the specific detection of compound ubiquitination by HUWE1^FL^. Analyses of an additional BI8626 derivative (**5**; Table 1) revealed that a removal of the upper, piperazine-linked benzyl moiety of the compound maintains considerable inhibition (Supplementary Fig. 18c). Substituting this group with an alkyne handle (pentynyl derivative **6** and propargyl derivative **8**, Table 1) preserved inhibition to a similar extent (Supplementary Fig. 18c, d), enabling us to selectively visualize the small-molecule ubiquitination by SDS-PAGE, following click-chemistry labeling of the compound with an azide-linked fluorophore (Fig. 4g). Proof-of-concept assays showed fluorescent signals corresponding to the mono- and diubiquitinated forms of derivative compound **6**, but not of its amine-deprived derivative (**7**, Table 1) within 10 min in an ATP-dependent manner (Fig. 4h). The modification was formed dose-dependently in the presence of MCL1, the ubiquitination of which, selectively monitored by immunoblotting (IB), decreased as the compound modification increased (Fig. 4i). Together, these analyses demonstrate (i) that HUWE1^FL^ ubiquitinates these drug-like molecules in vitro, (ii) that BI8622 and BI8626 are inhibitors of HUWE1^FL^, aside from the catalytic HECT domain, and (iii) that they likely compete with protein substrates for their target ligase.

### HUWE1 inhibitor ubiquitination occurs in cells

**Fig. 5:**
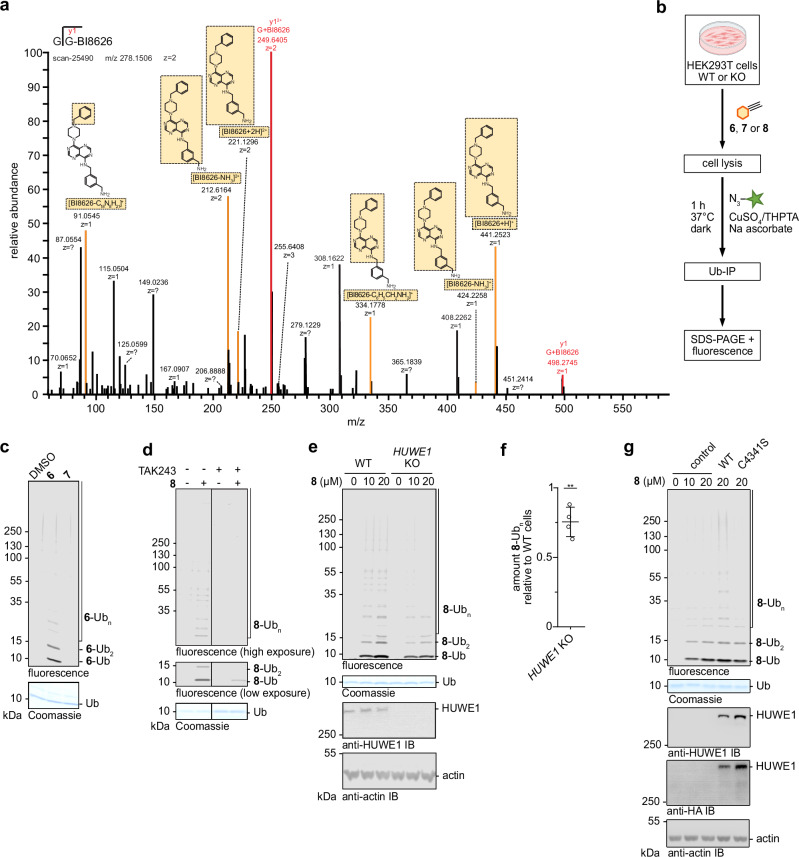
Ubiquitination of BI8626 derivatives occurs in cells. **a** Representative targeted MS² spectrum of a BI8626-modified C-terminal Ub peptide (precursor m/z 278.1506, z = 2), generated by tryptic digestion of cell lysates, following Ub-IP. Annotated fragment ions at m/z 249.6405 (z = 2) and 498.2745 (z = 1) correspond to a glycine–BI8626 conjugate. Additional fragment ions derived from BI8626 itself are observed at m/z 91.0545 (z = 1), 212.6164 (z = 2), 221.1296 (z = 2), 334.1778 (z = 1), 424.2258 (z = 1), and 441.2523 (z = 1), corresponding to characteristic cleavages of the compound. Structural representations of key fragmentation events are indicated, with boxed parts corresponding to the respective fragment ions. **b** Cartoon of our click chemistry-based strategy for detecting inhibitor ubiquitination in cell lysates. Elements of this figure were created in BioRender. Pohl, P. (2025) https://BioRender.com/sygo5n6. **c** Proof-of-principle experiment for the click chemistry-based detection of cell-based derivative **6** ubiquitination according to (**b**). Additionally, the total protein is shown by Coomassie staining (bottom). Compound **7** is clickable, but devoid of a primary amino group, providing a negative control. **d** Click-chemistry-based detection of cellular derivative **8** ubiquitination in the absence and presence of the UBA1 inhibitor TAK243, analogous to (**c**). Note that compound **8** is used here instead of **6**, for it provided enhanced signal (see Methods). The two sections are part of the same gel, shown with different exposures. **e** Click-chemistry-based detection of cellular derivative **8** ubiquitination in HEK293T WT and *HUWE1* KO cells, analogous to (**c**). Additionally, the HUWE1 level and an actin-loading control are shown based on IB. **f** Quantification of the ubiquitinated compound (across all species, including **8**-Ub, **8**-Ub_2_ and **8**-Ub_n_) from (**e**), normalized to WT cells. Data are represented as mean ± SD (*n* = 4 biological replicates). Statistical analysis with an unpaired, two-sided *t*-test **p = 0.0036. **g** Click-chemistry-based detection of cellular derivative **8** ubiquitination in HEK293T *HUWE1* KO cells, analogous to (**c**), upon overexpression of the indicated HUWE1 variants. C4341S catalytically inactive. The HUWE1 level and an actin-loading control are shown based on IB. Source data are provided as a Source Data file.

We next investigated BI8626 ubiquitination in the cellular context. To this end, we treated HEK293T cells with the compound for 1 h, followed by cell lysis, nanobody-based pull-down of Ub, tryptic digestion, and MS/MS analyses of the Ub-derived peptides. Intriguingly, targeted MS² spectra of the precursor ion at m/z 278.15 revealed characteristic fragment ions of BI8626 and signals corresponding to a glycine–BI8626 fragment (at m/z 249.64 (z = 2) and 498.27 (z = 1)), indicating that BI8626 is indeed ubiquitinated in cells (Fig. 5a).

To dissect the requirements of cellular compound ubiquitination we adapted our click-chemistry strategy for the detection of the alkyne-modified BI8626 derivatives in cell lysates (Fig. 5b). Both mono- and polyubiquitinated forms of derivatives **6** and **8** were detected, in contrast to derivative **7** that lacks a primary amino group, providing evidence for the specificity of our readout (Fig. 5c, d). In the following, we focused on the propargyl derivative **8**, since it yielded slightly stronger signal in cells than the pentynyl derivative **6** (Table 1). Our analyses reveal that the cell-based ubiquitination of the compound is markedly reduced in the presence of the E1 inhibitor TAK243, suggesting that the E1-activated ubiquitination cascade is required for the modification, in line with our in vitro results (Fig. 5d).

To interrogate whether the cellular compound ubiquitination depends on HUWE1, we used CRISPR/Cas9-based gene editing to knock out *HUWE1* in HEK293T cells. Interestingly, derivative **8** ubiquitination was still detected in this context, albeit at slightly reduced levels (Fig. 5e). Quantitative analyses indicate that endogenous HUWE1 accounts for no more than ~25% of the total signal in this set-up (Fig. 5f). Consistent with our comparative analyses of purified HECT domains, these cell-based data thus suggest that other ubiquitination enzymes can modify drug-like small molecules in cells. While future studies are required to identify those enzymes, cell treatment with the NAE (NEDD8-activating enzyme)-inhibitor MLN4924 that broadly blocks cullin-RING ligase (CRL) activities^39^, does not markedly affect derivative **8** ubiquitination (Supplementary Fig. 19a). Overexpression of WT HUWE1, but not the catalytically inactive C4341A variant (Fig. 5g) enhances compound ubiquitination in the *HUWE1* knock out context, confirming that the HUWE1-driven modification requires the catalytic activity of the ligase. Taken together, our analyses demonstrate that BI8626 and derivatives thereof provide substrates of the ubiquitination machinery in human cells, revealing that the vast capacities of the Ub system in substrate modification comprise exogenous small-molecule. Compared to reconstituted in vitro reactions in which BI8626 selectively inhibits HUWE1^HECT^ and HUWE1^FL^ by competing with protein substrates for ubiquitination, the situation is more complex in the cell, where a major fraction of the compound ubiquitination is not HUWE1-dependent. Moreover, cellular compound ubiquitination may be dynamic, as proof-of-principle in vitro studies with the promiscuous deubiquitinase (DUB) USP21 show that BI8626-Ub is cleavable (Supplementary Fig. 19b).

Finally, to assess the cellular effects of BI8626, we performed quantitative proteomics of HEK293T cells upon 24-h exposure to the compound, compared to control cells. Total proteome analyses demonstrate widespread compound-induced changes, with numerous proteins becoming either significantly more or less abundant (Supplementary Fig. 20a). Additional Ub remnant profiling (diGly proteomics) reveals predominantly reductions in protein ubiquitination upon BI8626 treatment, in line with perturbations of the Ub system (Supplementary Fig. 20b). Significantly downregulated ubiquitination sites are found in HUWE1 and several of its substrates, including MCL1^36^, HAPSTR1^40^, and various histones^41^, but also in many other proteins not known to be specifically ubiquitinated by HUWE1. In principle, these data are thus compatible with the notion that BI8626 can interfere with HUWE1 activity or HUWE1-related pathways in cells; however, they are also consistent with our finding that the compound lacks cellular specificity for HUWE1 and perturbs protein ubiquitination more broadly.

## Discussion

Deregulation of the Ub system is linked to manifold human diseases, rendering it a prime arena for drug discovery^42^. In particular, the highly diversified Ub ligases provide key therapeutic opportunities, due to their specificity in substrate selection and modification. This potential has first been realized by immunomodulatory imide drugs (IMiDs) that reprogram the cullin-RING ligase CRL4^CRBN^ toward pathogenic neo-substrates, with transformative clinical success in treating hematological malignancies^43^. The development of additional targeted protein degraders has made staggering progress in recent years, with over 20 proteolysis-targeting chimeras (PROTACs) and molecular glues entering clinical trials^44^. The common principle underlying these small-molecule effectors is to harness, rather than to block, cellular Ub ligase activities. Here we reveal that exogenously administered compounds can not only be used to manipulate Ub ligases, but can serve as substrate for ubiquitination in the cell. A related, yet distinct observation has been described in a recent preprint, in which a small molecule from a diversity-oriented synthesis library was found to be specifically ubiquitinated by RNF19-type ligases^45^. In contrast, our work focuses on a class of compounds marketed as selective inhibitors of the tumor-associated HECT-type E3 HUWE1, which are frequently used as research tools.

In particular, our analyses reveal that HUWE1 selectively ubiquitinates the drug-like small molecules BI8622, BI8626, and derivatives thereof, provided they contain a primary amino group. The modification follows a canonical ubiquitination mechanism, requiring E1, E2, and E3 activities, as well as the C-terminal di-glycine motif of Ub. Consistently, the small molecule-Ub amide linkage is cleavable by DUBs, as demonstrated for USP21. This suggests that cellular compound ubiquitination may, in principle, be regulated by the combined actions of ubiquitination enzymes and DUBs. Comparative in vitro studies show that a number of HECT domains beyond HUWE1 have the ability to ubiquitinate BI8622 and BI8626, albeit with markedly different efficiencies. The compounds thus provide selective HUWE1^HECT^ and HUWE1^FL^ substrates, enabling them to function as substrate-competitive inhibitors in an in vitro setting. This mechanism accounts for the identification of the compounds in a HTS^31^, in which HECT domain autoubiquitination was monitored after removal of other reaction components, causing compound ubiquitination to escape detection. In the same vein, it highlights the necessity for caution when screening Ub ligase activities using compound libraries containing modifiable groups.

Using mass spectrometry and click chemistry-based strategies, we demonstrate that BI8626 and derivatives thereof are also ubiquitinated in cells. In this context, the modification is promoted by the catalytic activity of HUWE1, but not dependent on it, with HUWE1 contributing only a minor fraction of total compound ubiquitination. This observation implies that BI8626 does not confer cellular specificity for HUWE1 and that other ubiquitination enzymes have the capacity to act on drug-like small-molecule substrates. In line with this, BI8626 elicits widespread global proteomic perturbations and suppresses ubiquitination at numerous protein sites. While some of the affected proteins are direct targets of HUWE1, our data show that BI8626 exerts broad effects on the Ub system. Thus, to convert the existing compound scaffold into a specific cellular substrate of HUWE1 would require a considerable increase in specificity. It should also be noted, that the particular impact of BI8626 may vary with the cellular context, potentially influenced by the expression levels or localization of those ligases targeting it. To understand whether the cytotoxicity of BI8626 results from a general disruption of the cellular Ub pool, from a competition with protein substrates for certain Ub ligases or from yet uncharacterized interactions of its ubiquitinated form, it will be important to identify the responsible ligases, characterize the dynamics of compound ubiquitination, and assess the lifetime and localization of the ubiquitinated compound within cells.

The mechanism uncovered in this study may be exploited to develop specific small-molecule tool substrates for cell-based analyses of Ub ligase activities, beyond HUWE1. Such compounds would provide valuable probes, specifically for E3s with a broad or poorly-defined protein substrate spectrum. Similarly, specific small-molecule substrates may be transformed into covalent inhibitors of catalytic cysteine-dependent E3s by substituting the modifiable group with an electrophilic warhead targeting the active site. Moreover, the concept of small-molecule ubiquitination in cells could be harnessed to convert suitable ‘prodrugs’ into novel functional modalities, including targeted protein degraders. In this case, a lack of ligase specificity in cellular compound ubiquitination, as identified in our system, could be beneficial. To fully explore the biotechnological and biomedical potential of exogenous small molecule ubiquitination, innovative analytical tools will be required, as implemented here.

## Methods

### DNA constructs

All genes analyzed here represent human sequences. The following plasmids used for recombinant bacterial expression were described previously: HUWE1^HECT^ and variants thereof^32,46^, E6AP^HECT^ ^47^, NEDD4^HECT^^[ 32^, HACE1^HECT^^[ 48^, UBE2L3^48^, chitin-binding domain-tagged Ub (a gift by David Komander)^49^, UBA1^50^, and Ub^50^. The *UBE3C*^*HECT*^ gene (encoding residues 692–1083) in pET49B was provided by Mateusz Ambrozkiewicz. *UBE2D3* was cloned into a modified pCCA1 vector^50^. The *MCL1* gene was cloned into pGEX-6-P3, using 5′-ggaagttctgttccaggggcccatgtttggcctcaaaagaaacgcggt-3′ (forward) and 5′-cgacccgggaattcggggatcccagttattagccaccttctaggtcctctacatgg-3′ (reverse) primers. The vector encoding USP21 (residues 196–565) was a gift from David Komander (http://n2t.net/addgene:61585; RRID: Addgene_61585). The pDARMO_CMVT_3xFLAG-HUWE1_R4187C vector for the preparation of HUWE1^FL^ from mammalian cells (a gift from Eric Fischer; http://n2t.net/addgene:187142; RRID: Addgene_187142)^51^ was mutated to encode the WT sequence, using 5′-ggaaagaagacatggctgtgcatgtccgtc-3′ (forward) and 5′-ctgaatgccattcatgccttcgagggcagc-3′ (reverse) primers. The pCMV vectors for transient transfections of HA-tagged HUWE1^FL^ (WT and C4341S) in mammalian cells were a gift from Manabu Kurokawa^52^. All point mutations were introduced by restriction-free methods.

### Protein preparation

The expression and purification of the following proteins followed published protocols: HUWE1^HECT^ ^46^, HUWE1^HECTΔ4^ (residues 3993–4370)^32^, E6AP^HECT^ ^47^, NEDD4^HECT^ ^47^, HACE1^HECT^ ^48^, UBA1^50^, UBE2L3^47^, UBE2D3^53^, and Ub^50^. The preparation of the vinylthioether-linked HUWE1^HECT^-Ub complex was also previously described^32^. UBE3C^HECT^ was expressed in *E. coli* LOBSTR-BL21(DE3)-RIL at 15 °C overnight, and the cells were lysed in 50 mM Tris (pH 7.5), 200 mM NaCl, 5 mM DTT, and 4 mM MgCl_2_. The cleared lysate was incubated with Glutathione Sepharose 4B (Cytiva) at 4 °C for 2 h, the resin washed 3 x with lysis buffer, resuspended as a 50% slurry, and on-bead 3 C protease cleavage performed overnight at 4 °C, followed by size-exclusion chromatography (SEC) in 10 mM Tris (pH 7.8), 150 mM NaCl, 4 mM MgCl_2_, and 2 mM TCEP (HiLoad Superdex 16/600 200 pg; Cytiva).

MCL1 was expressed in *E. coli* BL21(DE3) at 18 °C overnight, and the cells lysed in PBS (pH 7.4), 500 mM NaCl, 5 mM EDTA and 5 mM DTT. The cleared lysate was incubated with Glutathione Sepharose 4B at 4 °C for 1 h, the resin washed with 20 mM HEPES (pH 7.5), 500 mM NaCl, 2 mM EDTA, 5 mM DTT, and 10% (w/v) glycerol, and GST-tagged MCL1 eluted with the same buffer, supplemented with 20 mM reduced glutathione, followed by overnight cleavage with 3 C protease during dialysis into 20 mM HEPES (pH 7.5), 300 mM NaCl, 2 mM EDTA, 0.5 mM TCEP, and 10% (v/v) glycerol. The cleaved protein was concentrated and subjected to SEC in 20 mM HEPES (pH 7.5), 150 mM NaCl, 0.5 mM TCEP, and 10% glycerol (HiLoad Superdex 16/600 75 pg; Cytiva). Residual GST was removed by a final polishing step, using a GSTrap™ HP column (Cytiva).

USP21 was expressed in *E. coli* LOBSTR-BL21(DE3)-RIL at 20 °C overnight and purified as previously described^54^. In brief, the cells were lysed in 50 mM Tris (pH 7.4), 300 mM NaCl and 10 mM imidazole, followed by Ni-affinity chromatography, overnight dialysis into 25 mM Tris pH 7.4, 200 mM NaCl and 5 mM DTT at 4 °C in the presence of ULP1 protease, followed by SEC (HiLoad Superdex 16/600 75 pg).

For the preparation of HUWE1^FL^, HEK293T cells were seeded one day prior to transfection in DMEM, high-glucose, pyruvate (Thermo Fisher Scientific), supplemented with 10% (v/v) FBS (PAN Biotech) and 1% (v/v) penicillin-streptomycin (Sigma-Aldrich). 24 h after transfection with Lipofectamine 3000 (Thermo Fisher Scientific), the cells were collected in ice-cold 1 x DPBS (Thermo Fisher Scientific) and resuspended in 50 mM HEPES-KOH (pH 7.4), 200 mM NaCl, 2 mM MgCl_2_, 5% glycerol (w/v), 1% (v/v) Triton X-100, and protease-phosphatase inhibitor cocktail (Sigma-Aldrich), before lysis at 4 °C for 30 min. The clarified lysate was incubated at 4 °C for 60 min with Anti-Flag M2 magnetic beads (Merck), equilibrated in the same buffer and the resin was washed 3 x with 50 mM HEPES-KOH (pH 7.4), 200 mM NaCl, 2 mM MgCl_2_, 5% glycerol (w/v), and protease-phosphatase inhibitor cocktail. HUWE1^FL^ was eluted with the same buffer, including 250 μg mL^−1^ 3 x Flag peptide (MedChemExpress), then supplemented with 1 mM TCEP and purified by SEC (Superose 6 10/300 GL, Cytiva) in 30 mM HEPES-KOH (pH 7.4), 150 mM NaCl and 1 mM TCEP.

The BI8626–Ub adduct was prepared from ubiquitination reactions containing 200 nM UBA1, 2.5 μM UBE2L3, 1 μM HUWE1^HECT^, 100 μM ubiquitin, and 120 μM BI8626 in 25 mM HEPES (pH 7.5), 100 mM NaCl, 3 mM MgCl₂, 1 mM ATP, and 0.5 mM TCEP. After 2 h at 30 °C, the reactions were diluted into 50 mM ammonium acetate (pH 4.5) and purified via a HiTrap™ SP HP cation exchange column (Cytiva), using a linear gradient up to 500 mM NaCl. Eluted fractions were pooled, concentrated, and further purified by size-exclusion chromatography (SEC), using a HiLoad Superdex 75 16/600 pg column (Cytiva).

Recombinant N-terminally His-tagged human histone 2B (residues 2–126) and FOXP3 (residues 1–260) were purchased from BPS Bioscience and Antikoerper Online, respectively.

### Enzymatic assays

E3-free multi-turnover ubiquitination reactions to monitor E1 and E2 activities contained 1 μM UBA1, 5 μM UBE2L3, 100 μM Ub, 2 mM ATP and 10 mM MgCl_2_ in 25 mM HEPES (pH 7.4), and 0.2 mM TCEP. Reactions were incubated at 30 °C for 10 min (or otherwise, as specified) and quenched with 50 mM EDTA, followed by the addition of SDS-loading dye. Single-turnover E2~Ub discharge assays were performed similarly to previous studies^35^. In short, 1 μM UBA1, 5 μM UBE2L3, and 100 μM Ub were pre-incubated with 2 mM ATP and 10 mM MgCl_2_ in 25 mM HEPES (pH 7.4) at 30 °C for 30 min, diluted 4-fold, and quenched with 50 mM EDTA. Thereafter, 1.2 μM of the UBE2L3~Ub product was incubated with 5 μM HUWE1^HECT^ at 30 °C for 1 min.

HUWE1^HECT^ multi-turnover ubiquitination reactions were performed for 30 minutes (unless stated otherwise) with 200 nM UBA1, 5 μM UBE2L3 or UBE2D3 (as specified), 1 μM HUWE1^HECT^ and 30 μM Ub in 25 mM HEPES (pH 7.5), 100 mM NaCl, 3 mM MgCl_2_, and 0.5 mM TCEP. HUWE1^FL^ multi-turnover ubiquitination reactions included 50 nM UBA1, 2.5 μM UBE2L3, 0.05 μM HUWE1^FL^, 0.5 μM MCL1 (or 0.5 μM H2B or 1 μM FOXP3) and 20 μM Ub in 20 mM HEPES (pH 7.5), 100 mM NaCl, 3 mM MgCl_2_, and 0.5 mM TCEP. All multi-turnover reactions were started by the addition of 1 mM ATP, incubated at 30 °C, quenched with SDS-loading dye. If not stated otherwise, HUWE1^FL^autoubiquitination reactions were incubated for 20 minutes. Substrate ubiquitination reactions with MCL1 or H2B were incubated for 10 and 20 min, respectively; those with FOXP3 for 30 min. Those reactions that were monitored by fluorescence contained a fluorescent Ub tracer (IRDye 800CW maleimide; LI-COR)^55^ and were monitored using an Odyssey CLx scanner (LI-COR), followed by Coomassie staining. In the fluorescence images, only those marker bands are labeled (in kDa) that are visible by fluorescence; all marker bands are labeled in the Coomassie-stained images. Statistical analyses were performed with Prism 9 v.9.3.1 (GraphPad).

For deubiquitinase assays, 10 μM BI8626-Ub were incubated with the indicated concentrations of USP21 at 30 °C for 30 min and analyzed on a Superdex 75 Increase 3.2/300 column (Cytiva) in 25 mM HEPES (pH 7.5), 100 mM NaCl, and 0.5 mM TCEP at 4 °C.

### Differential scanning fluorimetry (DSF)

DSF was performed in 25 mM HEPES (pH 7.5), 100 mM NaCl, 1 mM TCEP and 0.4% (v/v) DMSO, using a CFX96 Touch Real-Time PCR Detection System (BioRad). 10 μM HUWE1^HECT^ and HUWE1^HECT^-Ub, respectively, at a sample volume of 25 μL were denatured by a linear 25–95 °C gradient over 120 min in a presence of 10 x SYPRO Orange (Invitrogen). T_m_-values were determined as the first derivative of the fluorescence curves, using CFX Maestro™ v.1.1 (Bio-Rad). Melting curves were plotted as normalized -d(fluorescence)/dt with Prism v.9.3.1 (GraphPad).

### Isothermal titration calorimetry (ITC)

ITC was performed with an ITC200 calorimeter (MicroCal) at 25 °C. The proteins were dialyzed into 20 mM HEPES (pH 7.5), 150 mM NaCl, and 1 mM β-mercaptoethanol (β-ME) overnight. 600 μM HUWE1^HECT^ or HUWE1^HECT^-Ub were applied to the cell and a 10-fold molar excess of BI8626 to the syringe (and vice versa). The reference cell was filled with buffer. Titration experiments included 1 injection of 1.5 μL, followed by 15 injections of 2.4 μL in 3-s intervals, with 150-s spacings between individual injections. The reference power was set to 12 μcal s^−1^ and the stirring speed to 270 rpm. To control for instrument functionality, 5 mM CaCl_2_ were titrated into 0.4 mM EDTA in 10 mM MES (pH 5.6). Data were plotted with OriginPro v.2020b (OriginLab).

### Analytical SEC

HUWE1^HECT^ multi-turnover ubiquitination reactions were performed with 500 nM UBA1, 5 μM UBE2L3, 5 μM HUWE1^HECT^ and 100 μM Ub in 20 mM HEPES (pH 7.5), 100 mM NaCl and 0.5 mM TCEP in the presence of 1 mM ATP and 3 mM MgCl_2_. After 15 min incubation at 30 °C, the mixture was injected into a Superdex 75 Increase 3.2/300 column (Cytiva), equilibrated in 25 mM HEPES (pH 7.5), 100 mM NaCl, and 0.5 mM TCEP at 4 °C. Chromatograms were analyzed and peaks integrated with Unicorn v.7.9.02440 (Cytiva). Data were plotted with Prism v.9.3.1 (GraphPad).

### MS analyses of compound ubiquitination

Intact protein LC/MS: protein molecular weights (MWs) were determined by LC/MS on a nanoflow chromatography system (Dionex U3000 nanoRSLC), hyphenated to a hybrid quadrupole/orbitrap mass spectrometer (Q Exactive HF) with a NanoFlex ion source and controlled by Xcalibur v.2.4 (all Thermo Fisher Scientific). Proteins were dissolved in 2% (v/v) acetonitrile (ACN), 0.1% (v/v) formic acid (FA) to a concentration of 100 fmol μL^−1^. For each analysis, 500 fmol were enriched on a pre-column (0.15 ×20 mm, Reprosil-Pur120 C18-AQ 5 μm; Dr. Maisch GmbH) and separated on an analytical RP-C18 column (0.075 mm×200 mm, Reprosil-Pur 120 C18-AQ; 1.9 μm, Dr. Maisch GmbH), using a 23-min linear gradient of 10–80% (v/v) ACN/0.1% (v/v) FA at 300 nl min^−1^. MS data were acquired in full MS mode across the 500–1800 m/z-range at a resolution setting of 120,000 FWHM, an AGC target of 1*10e6 and a maximum fill time of 200 ms. Charge state deconvolution and MW representation were performed in Freestyle v.1.9 (Thermo Fisher Scientific), using default settings for high-resolution data.

LC/MS/MS of ubiquitinated inhibitors from reconstituted HUWE1^HECT^-driven reactions: Proteins were separated by SDS-PAGE, followed by Coomassie staining, and the protein band of interest was cut out. Gel slices were reduced with 10 mM DTT at 56 °C for 55 min, alkylated with 55 mM chloroacetamide at 26 °C for 20 min, and digested with 0.2 μg LysC protease (Mass Spec Grade, Promega) at 37 °C overnight. The extracted peptides were dried in a speed-vac and then resuspended in 2% (v/v) ACN, 0.1% (v/v) FA, and analyzed on an Orbitrap Exploris 480 mass spectrometer, coupled to a Dionex U3000 nanoHPLC system (Thermo Fisher Scientific). Peptides were pre-concentrated and desalted on a trap column (PepMap, C18, 5 μm, 0.3 × 5 mm; Thermo Fisher Scientific) at 10 μL min^−1^ in the above solvent, followed by the separation on a home-made capillary column (300 × 0.075 mm, ReproSil-Pur 120 C18-AQ, 1.9 μm; Dr Maisch GmbH), using a 58-min linear gradient from 10–45% of 0.08% (v/v) FA in 80% (v/v) ACN) versus 0.1% (v/v) FA at 300 nl min^−1^. The mass spectrometer was operated in data-dependent acquisition (DDA) mode with a top-20 method, using higher-energy collisional dissociation (HCD) with an isolation width of 1.6 m/z and a collision energy setting of 28%. MS spectra across an m/z-range of 350–1600 were acquired at a resolution setting of 120,000 FWHM at m/z 200, and MS/MS spectra at a resolution setting of 30,000. Normalized AGC target (%) and maximum injection time (IT) for MS and MS/MS were set to 100 in 60 ms and 100 in 120 ms, respectively. Fixed first mass and dynamic exclusion values were set to 100 m/z and 9 s, respectively. Raw files were processed using MaxQuant v.2.0.3.0 (Max Planck Institute of Biochemistry)^56^. MS/MS spectra were searched against a UniProtKB human database containing 20422 protein entries (March 2023), supplemented with the human Ub sequence via the Andromeda search engine^57^. Precursor and fragment ion mass tolerances were set to 6 and 20 ppm, respectively. BI8622 (change: 408.2062448016; position: protein C-term; type: Standard; new terminus: none) and BI8626 (change: 422.2331282541; position: protein C-term; type: standard; new terminus: none) were added to the modifications database in MaxQuant. Protein C-terminal BI8622, BI8626, and methionine oxidation were allowed as variable modifications. Cysteine carbamidomethylation was defined as a fixed modification. The minimum peptide length was set to 7 amino acids, with a maximum of 2 missed cleavages. The false discovery rate (FDR) was set to 1% at both the peptide and the protein level, using a forward and reverse concatenated decoy database approach.

Samples for LC/MS/MS analyses of the ubiquitinated inhibitors from reconstituted HUWE1^FL^-driven reactions were prepared by in-solution trypsin (Mass Spec Grade, Promega) digestion, instead of the gel-based LysC digestion procedure described above. This change was made, as it yielded superior signal and thus facilitated the detection of small-molecule ubiquitination from both in vitro reactions and cell lysates. In both cases, the samples were reduced and alkylated with 10 mM TCEP and 20 mM iodoacetamide at 37 °C in the dark for 60 min. An equal mixture of Sera-Mag SpeedBeads with hydrophilic and hydrophobic surfaces (GE Healthcare; 1:1 mix (v/v)) was washed twice with water and added to the sample at 50:1(w/w) resin-to-protein ratio. After adding ACN to a final percentage of 70% (v/v) and removing the supernatant, the resin was washed 3 x with 90% (v/v) ACN, resuspended in 50 mM triethylammonium bicarbonate (TEAB), containing sequencing-grade modified trypsin (1:25 (w/w) enzyme-to-protein ratio), and incubated at 37 °C, shaking at 1,000 rpm overnight. The resulting peptides were then dried, dissolved in 5% ACN/0.1% FA (both v/v), and measured on a hybrid quadruple-Obitrap Exploris480 mass spectrometer (Thermo Fisher Scientific) coupled to an Ultimate 3000 UHPLC system (Thermo Fisher Scientific). To this end, peptides were pre-concentrated and desalted on a trap column (PepMap, C18, 5 μm, 0.3 × 5 mm; Thermo Fisher Scientific) at 10 μL min^−1^ in loading buffer (2% (v/v) ACN, 0.1% FA), followed by the separation on a self-made capillary column (ReproSil-Pur 120 C18-AQ, 1.9 μm, 300 × 0.075 mm; Dr Maisch GmbH), using a 63-min linear gradient from 10–38% of 0.08% (v/v) FA in 80% ACN/0.1% FA (both v/v) at 300 nl min^−1^. Two MS measurements were performed, including full MS and targeted MS/MS scans (Full MS/tMS2). Full MS settings: detector type: Orbitrap; resolution: 120,000; *m/z*-range: 150–1300; RF lens (%)−50; AGC target: standard, maximum IT (ms): auto, data type: profile. tMS2 settings: isolation window (*m/z*): 1.4; activation type: HCD; HCD collision energy (%): 28; detector type: Orbitrap; Orbitrap resolution: 15,000; AGC target: standard; maximum IT (ms): 54; data type: profile. For a list of targeted masses, see Supplementary Table.

Sample preparation for LC/MS/MS detection of ubiquitinated compounds from cell lysates was performed according to the following workflow: HEK293T cells at 70% confluency were treated with 15 μM BI8626 or an equal volume of DMSO for 1 h. Cells were collected in ice-cold 1 x DPBS and resuspended in sodium phosphate buffer (pH 7.0), supplemented with 1% (v/v) NP-40, 3 M urea, 10 mM N-ethylmaleimide (NEM), 50 μM PR-619 (Sigma-Aldrich), 10 μM MG-132 (Thermo Fisher Scientific) and protease-phosphatase inhibitor cocktail. After 20 min incubation on a rotating wheel at 4 °C, the lysate was cleared by centrifugation, the total protein concentrations determined, and the sample (2.3 mg) diluted with 10 mM Tris (pH 7.5), 150 mM NaCl and 0.5 mM EDTA to a final volume of 1 mL. 25 μL of Ubiquitin-Trap Magnetic Agarose beads (Proteintech) were added and the mixture was rotated at 4 °C for 80 min, before washing, once with 10 mM Tris (pH 7.5), 150 mM NaCl, 0.05% (v/v) NP-40, 0.5 mM EDTA and 3 M urea and twice with 10 mM Tris (pH 7.5), 150 mM NaCl, 0.05% (v/v) NP-40, 0.5 mM EDTA and elution in 40 μL of 4% (w/v) SDS in 50 mM Tris (pH 7.5) at RT, followed by concentration determination with a Pierce BCA Protein Assay Kit (Thermo Fisher Scientific). Thereafter, the samples were treated and analyzed, as described above for the HUWE1^FL^-driven reconstituted ubiquitination reactions.

### HDX-MS

Two experiments were performed: 50 μM HUWE1^HECT^ (1) or HUWE1^HECT^-Ub conjugate (2) were incubated with 500 μM of BI8626 or BI8622 for 1 h on ice prior HDX-MS. Individual HDX reactions were generated with a 2-arm autosampler (LEAP Technologies) by mixing 6.5 μL sample with 58.5 μL of HDX buffer (20 mM HEPES pH 8.0, 150 mM NaCl, 5 mM DTT) prepared with 99.9% D_2_O, incubation at 25 °C for various times, before quenching by mixing 55 μL with an equal volume of 400 mM KH_2_PO_4_/H_3_PO_4_ (pH 2.2) and 2 M guanidine-HCl (precooled at 1 °C). Non-deuterated samples were generated analogously by 10-fold dilution with H_2_O-based buffer. 95 μL of the quenched reactions were injected into an ACQUITY UPLC M-class system with HDX technology (Waters)^58^ through a 50 μL loop, following digestion for 3 min (solvent: H_2_O + 0.1% (v/v) FA (100 μL min^−1^)) by passing them through a cartridge (2 mm × 2 cm, 12 °C) of immobilized protease. The resulting peptides were trapped on an AQUITY UPLC BEH C18 VanGuard column (2.1 × 5 mm, 1.7 μm; Waters) at 0.5 °C. In (1), the sample was split by a T-junction for digestion with porcine pepsin or a 1:1 mixture of protease type XVIII from *Rhizopus* sp. and protease type XIII from *Aspergillus saitoi*, and these digests combined with another T-junction. In (2), digestion was conducted with porcine pepsin or the protease type XVIII/XIII mixture, and the resulting datasets merged during subsequent analysis (see below). The trap column was then placed in line with an ACQUITY UPLC BEH C18 column (1.0 ×100 mm, 1.7 μm; Waters), and peptides eluted at 0.5 °C with a gradient of H_2_O + 0.1% (v/v) FA (solvent A) and ACN + 0.1% (v/v) FA (solvent B) at 30 μL min^−1^ as follows: 0-7 min: 95–65% A; 7–8 min: 65–15% A; 8–10 min: 15% A; 10–11 min: 5% A; 11–16 min: 95% A. Peptides were ionized by electrospray ionization (250 °C capillary temperature, 3.0 kV spray voltage) and mass spectra acquired on a G2-Si HDMS with ion-mobility separation (Waters) in positive-ion mode from 50 to 2,000 m/z using enhanced high-definition MS (HDMS^E^) and high-definition MS (HDMS) modes for non-deuterated and deuterated samples, respectively^59,60^. Lock-mass correction was implemented with [Glu1]-fibrinopeptide B standard (Waters). During peptide separation, the protease-loaded column was washed with 3 × 80 μL 0.5 M guanidine-HCl in 4% (v/v) ACN; blank injections were performed between each sample. For each protein state and time-point, three individual HDX reactions were measured.

Peptide identification and HDX analysis was performed with ProteinLynx Global SERVER v.3.0.1 (PLGS; Waters) and DynamX v.3.0 (Waters), as described previously^61^. In brief, peptides were identified from the non-deuterated samples acquired with HDMS^E^, using low-energy, elevated-energy, and intensity thresholds of 300, 100, or 1000 counts, respectively. Identified ions were matched to peptides with a database containing the amino acid sequences of HUWE1^HECT^, porcine pepsin, and their reversed sequences (peptide tolerance: automatic; fragment tolerance: automatic; min fragment ion matches per peptide: 1; min fragment ion matches per protein: 7; min peptide matches per protein: 3; maximum hits to return: 20; maximum protein mass: 250,000; primary digest reagent: non-specific; missed cleavages: 0; false discovery rate: 100). Only peptides with an intensity >10,000 counts, $\le\!$30 residues, $\ge \!$2 products with $\ge \!$0.05 products/residue, $\le \!$25 ppm mass error and retention time tolerance of 0.5 min were analysed. All spectra were manually inspected and, if necessary, individual samples or/and peptides excluded from the analysis. The maximal deuterium uptake of a given peptide was calculated by the number of residues minus one for the N-terminal residue and minus the number of prolines. To render the residue-specific HDX differences from overlapping peptides, the shortest peptide covering a certain residue was used; where multiple peptides were of the shortest length, the peptide with the residue closest to the peptide’s C-terminus was utilized.

### Quantitative proteomics

For total proteomics and Ub remnant profiling (diGly proteomics), HEK293T cells were treated with 15 μM BI8626 (in DMSO) or an equivalent volume of DMSO for 24 h, washed with ice-cold 1 x DPBS, and lysed in 9 M urea, 50 mM Tris (pH 8.0) and 150 mM NaCl, supplemented with protease inhibitor (Roche) and 50 µM PR-619, and processed essentially as described^62^. Briefly, the cells were lysed by sonication, non-solubilized material removed by centrifugation, and the protein concentration was determined with a Pierce BCA Protein Assay Kit. 5 mg was used for further MS processing and four biological replicates per condition.

After reduction with 5 mM DTT and alkylation with 10 mM chloroacetamide, followed by quenching with 5 mM DTT, lysates were digested with 5 ng/μl LysC protease (Wako) at RT for 1 h. Subsequent digestion of peptides with trypsin (Promega) at a 1: 200 enzyme: peptide ratio was performed^63^. Desalted and lyophilized peptides were resuspended in 1.5 ml IAP buffer (50 mM MOPS (pH 7.2), 10 mM Na_2_HPO_4_, 50 mM NaCl) and centrifuged to remove insoluble material. 0.1% of the digested peptides were kept in 200 μl 0.15% TFA for whole cell proteomic analyses. The residual supernatant was incubated with pre-equilibrated anti-diGly antibody (32 μg/IP) conjugated to protein A agarose beads (PTMScan® Ub Remnant Motif Kit; Cell Signaling) at 4 °C for 1 h. Each sample was immunoprecipitated sequentially two times. Unbound peptides were removed through 3x washing with IAP buffer and once with PBS. Bound material was eluted 4x with 50 μl 0.15% TFA on Micro Bio-Spin^TM^ columns (BioRad). Input and IP samples were desalted using custom-made C18 stage-tips (C18 material; Supelco Analytical) and solved in 0.1 % formic acid before applying it to LC-MS/MS^64^.

For MS data collection and analysis, samples were loaded on 75 μm × 15 cm custom-made fused silica capillary packed with C18AQ resin (Reprosil-PUR 120, 1.9 μm; Dr. Maisch) and separated using an Easy-nLC1200 liquid chromatograph (Thermo Fisher Scientific) followed by peptide detection on a Q Exactive HF mass spectrometer (Thermo Fisher Scientific) in data-dependent acquisition. The following buffer B (80% ACN in 0.1 formic acid) gradient was used at a flow rate of 250 nl/min: 3–6% for 2 min, 6–30% for 90 min, 30–44% for 20 min, 44–75% for 10 min, 75–100% for 5 min, 100% for 5 min, 100–3% for 5 min, 3% for 3 min. For diGly IP samples, peptides were separated on a 60 min buffer B gradient at a flow rate of 400 nl/min, as follows: 10–38% for 35 min, 38–60% for 5 min, 60–100% for 5 min, 100% for 5 min, 100–5% for 5 min, 5% for 5 min). Peptides were ionized using a Nanospray Flex Ion Source (Thermo Fisher Scientific) and identified in fullMS / ddMS² mode. For whole cell proteomics, the top 20 most intense peaks from each full MS scan were selected for subsequent fragmentation, with a dynamic exclusion of 120 s, excluding peptides with unassigned charge or charges of 1 or >8. For whole proteome analysis, the MS1 resolution was set to 120,000 with a scan range of 300–1700 m/z, MS2 to 15,000. AGC target1 was set to 3e6, AGC target2 to 1e5. For diGly IP samples, the top 5 most intense peaks were picked for fragmentation, with a dynamic exclusion of 40 s, excluding peptides with unassigned charge or charges of 1, 2 or >8. MS1 resolution was set to 60,000 with a scan range of 300–1750 m/z, MS2 to 30,000. AGC target1 was set to 1e6, AGC target2 to 5e5.

Data collection was controlled by Tune v.4.0.4084.22 (Thermo Scientific) and Xcalibur v.2.4 (Thermo Fisher Scientific). Raw data files from quadruplicate samples were analyzed using MaxQuant v.1.6.0.1 (Max Planck Institute of Biochemistry)^56,57^. Andromeda search engine in reversed decoymode based on a human reference proteome (Uniprot-FASTA, UP000005640, downloaded November 2024) with an FDR of 0.01 on the protein level. Digestion parameters were set to specific digestion with trypsin/p with a maximum number of 2 missed cleavage sites and minimum peptide length of 7. Methionine oxidation (15.994946), N-terminal protein acetylation (42.010565) and diGly remnant (114.042927; excluded from the C terminus) were set as variable modifications and carbamidomethylation of cysteine as fixed modifications. The peptide mass tolerance was set to 20 ppm (first search) and to 4.5 ppm (main search). Label-free quantification (with a minimum ratio count set to 2), re-quantification and match-between runs was selected. The resulting file for diGly modified peptides and the protein group file for the whole proteome were processed using Perseus v.1.6.14.0 (Max Planck Institute of Biochemistry)^65^. Common contaminants and reverse identifications were excluded. For the whole cell proteome, protein groups were further filtered for peptide count and MS/MS count ≥ 2 and a valid value in at least 3 out of 4 replicates per condition. All values were log_2_ transformed. Remaining missing values were replaced from a normal distribution with 0.3 width and 1.8 down shift calculated separately for each sample. For diGly modified peptides, peptides were filtered for a valid value in at least 3 out of 4 replicates. Similarly, the remaining missing values in the diGly peptide dataset were imputed. Statistical analyses were performed using a two-sided Two-Sample *t*-test with permutation-based FDR (*q* < 0.05).

### Computational simulations of BI8626 binding poses

To predict possible conformations for the nucleophilic attack of BI8626 on the HUWE1^HECT^ ~ Ub complex, MD simulations and AlphaFold3 v.3.0.1 (DeepMind; ‘AF3’)^66^ were employed. For the MD, we started from a crystal structure of a HUWE1^HECT^-Ub^32^, in which the Ub adopts the conserved donor orientation with respect to the HECT C-lobe and the HECT domain adopts an L-conformation, as required for aminolysis with protein substrates, assuming that the same conformation accommodates the reaction with BI8626. Details for the force field optimization, the simulations, and the filtering of the resulting poses are provided in the Supplementary Methods. With AF3, we initially predicted BI8626 binding to HUWE1^HECT^ in the presence of Ub and to a restrained HUWE1^HECT^~donor Ub complex, respectively; however, neither of those simulations showed highly-scoring models of BI8626 in a position compatible with a nucleophilic attack of the compound’s primary amino group on the C-terminal carbonyl group of ubiquitin. We thus incorporated restraints in AF3 to enforce this requirement. Details on the procedure are provided in the Supplementary Methods.

### Mammalian cell culture and genetic engineering

Commercial HEK293T (ATCC) were cultured in ‘DMEM, high glucose, pyruvate’ (Thermo Fisher Scientific) with 10% (v/v) fetal bovine serum (FBS) (Thermo Fisher Scientific), 1% (v/v) penicillin-streptomycin (Sigma-Aldrich), according to standard techniques at 37 °C in 5% CO_2_ and regularly tested for mycoplasma contaminations.

The protocol for CRISPR/Cas9-mediated *HUWE1*-knockout in HEK293T cells was previously described^67^. In short, two sgRNAs were cloned separately into pX330-U6-Chimeric_BB-CBh-hSpCas9 (a gift from Feng Zhang; http://n2t.net/addgene:42230; RRID:Addgene_42230)^68^ and pSpCas9(BB)−2A-GFP (PX458) (a gift from Feng Zhang; http://n2t.net/addgene:48138; RRID:Addgene_48138)^69^. Transfections were performed with Lipofectamine LTX (Thermo Fisher Scientific), single cells FACS-seeded into 96-well plates, and clones derived from single cells screened for *HUWE1* deficiency by sequencing and immunoblotting.

Transient transfections of HA-tagged HUWE1^FL^ WT and C4341S (Fig. 5g) were performed in antibiotic-free and FBS-free media using Lipofectamine 3000 (Thermo Fisher Scientific). 6 h post transfection the media was exchanged to media supplemented with antibiotics and FBS. 24 h post transfection cells were treated with 10 μM or 20 μM BI8626 derivative **8** for 1 h and then collected.

### Click chemistry

In vitro applications**:** Multi-turnover ubiquitination reactions were performed with 50 nM UBA1, 2.5 μM UBE2L3, 50 nM HUWE1^FL^ and 20 μM Ub in 20 mM HEPES (pH 7.5), 100 mM NaCl, 0.5 mM TCEP, and 20 μM derivative **6** or **7** ± 0.5 μM MCL1 at 30 °C for 10 min. The reactions were started by addition of 1 mM ATP/3 mM MgCl_2_ and quenched with 3 M urea. Click-chemistry labeling was performed by incubation of the samples with 20 μM IRDye 800CW Azide Infrared Dye (LI-COR), 5 mM CuSO_4_, 10 mM THPTA and 50 mM sodium ascorbate for 60 min at 37 °C in the dark, following by quenching with 40 mM EDTA. Following SDS-PAGE, the gels were fixed in 10% acetic acid and 30% ethanol for 2 ×15 min and washed in water overnight.

Cell lysate-based applications: HEK293T WT or *HUWE1* KO cells at ~70% confluency on a 10-cm dish were treated with 10 μM or 20 μM BI8626 derivative **6,** **7** or **8** for the indicated times. As indicated in the results section, derivative **8** yielded slightly stronger signal than **6** and was thus used for analyses of the prerequisites of cellular small-molecule ubiquitination. Treatment with 1 μM TAK243 (MedChemExpress) or 10 μM MLN4924 (Sigma-Aldrich) was performed for 1 h, prior to derivative **8** administration. Cells were collected with ice-cold 1 x DPBS, centrifugated, and the cell pellet was resuspended in 300 μL sodium phosphate buffer (pH 7.0) (Jena Bioscience), supplemented with 1% (v/v) NP-40, 3 M urea, 10 mM NEM, 50 μM PR-619, 10 μM MG-132 and protease-phosphatase inhibitor cocktail. After 20 min rotating at 4 °C, the lysate was cleared by centrifugation, the total protein concentration determined with a Pierce BCA Protein Assay Kit, the sample diluted to ~7.5 μg μL^−1^ and supplemented with 5 μM IRDye 800CW Azide (LI-COR), a mixture of 2 mM CuSO_4_/10 mM THPTA (Jena Bioscience) and 100 mM sodium ascorbate. The click chemistry reaction was performed in the dark at 37 °C, shaking at 300 rpm. After 1 h, the reaction was quenched with 40 mM EDTA, diluted 1.2-fold with 10 mM Tris (pH 7.5), 150 mM NaCl and 0.5 mM EDTA. 25 μL Ubiquitin-Trap Magnetic Agarose beads (Proteintech) were added, the mixture was rotated at 4 °C for 80 min, the resin washed 3 x with 10 mM Tris (pH 7.5), 150 mM NaCl, 0.05% (v/v) NP-40, 0.5 mM EDTA and 3 M urea, following protein elution in 40 μL of 1.5 x SDS-loading dye at 95 °C for 5 min. 10 μL were subjected to SDS-PAGE and analyzed by fluorescence scanning (Odyssey CLx; LI-COR) and Coomassie staining.

### Antibodies

The following primary antibodies were used: anti-Actin mouse monoclonal antibody (A1978, Sigma-Aldrich; dilution 1:1000); anti-HECTH9 AX8D1 mouse monoclonal antibody (5695, Cell Signaling Technology; dilution 1:1000), and anti-MCL1 D2W9E (94296, Cell Signaling Technology; dilution 1:1000), and anti-HA-HRP mouse monoclonal antibody (H6533, Sigma-Aldrich; dilution 1:4000). Secondary antibodies for luminescence-based detection included goat anti-rabbit HRP-linked antibody 7074 or goat anti-mouse HRP-linked antibody 7076 (both Cell Signaling Technology; dilution 1:10,000). For IP-MS, the PTMScan Ubiquitin Remnant Motif (K-ε-GG) Kit (5562, Cell Signaling Technology) was used.

### Compound synthesis

BI8626, BI8622, and derivative **7** were synthesized by WuXi AppTec. All other derivatives were synthesized in-house, as described below. An overview of the syntheses is provided in Supplementary Fig. 21. NMR spectra for the synthesized compounds 1, 2, 3, 4, 5, 6, and 8 are provided in Supplementary Figs. 22–28.

All chemicals and materials were procured from commercial providers and used without additional purification. For purifications of synthesis intermediates and final products, automated flash column chromatography was performed on Puriflash 430 or Puriflash XS520PLUS instruments (Interchim). Thin layer chromatography (TLC) was performed on silica gel-60-F_254_ aluminium plates (Merck) with UV detection at 254 nm and 366 nm. MS was carried out with a TLC-MS interface (Advion) with electrospray ionisation (ESI) in positive and/or negative mode and the following settings: ESI voltage: 3.50 kV, capillary voltage: 187 V, source voltage: 44 V, capillary temperature: 250 °C, desolvation gas temperature: 250 °C, and nitrogen flow: 5 L min^−1^. GC/MS analyses were carried out on a Hewlett Packard HP 6890 series GC-system equipped with a HP-5MS capillary column (00.025 mm × 300 mm, 25 μm film thickness) and a HP 5973 mass selective detector (EI ionization). Helium was used as carrier gas, employing a temperature gradient of 160–300 °C.

HPLC was performed on a 1100 system (Agilent Technologies) with a 1260 DAD (diode array detector) at 254, 230, and 280 nm. Method A: Kinetex (2.6 μm C8 100 Å 150 × 4.6 mm; Phenomenex) column, injection volume: 5 μL, flow rate: 0.5 mL min^−1^ at 23 °C; 0 min: 40% MeOH, 60% phosphate buffer (pH 2.3); 15 min: 85% MeOH, 15% phosphate buffer (pH 2.3); 20 min: 85% MeOH, 15% phosphate buffer (pH 2.3); 22 min: 40% MeOH, 60% phosphate buffer (pH 2.3); 28 min: 40% MeOH, 60% phosphate buffer (pH 2.3). Method B: Luna 5 u C8 RP (150 × 4,6 mm; 5 μm; Phenomenex) column, injection volume: 10 μL, flow rate: 1.5 mL min^−1^ at 35 °C; 0 min: 40% MeOH, 60% 0.01 M phosphate buffer (pH 2.3); 8 min: 85% MeOH, 15% phosphate buffer (pH 2.3); 13 min: 85% MeOH, 15% phosphate buffer (pH 2.3); 14 min: 40% MeOH, 60% phosphate buffer (pH 2.3); 16 min: 40% MeOH, 60% phosphate buffer (pH 2.3).

NMR spectra were recorded on Avance III HD 400 (^1^H: 400 MHz, ^13^C: 101 MHz) or Avance 200 (^1^H: 200 MHz, ^13^C: 50 MHz) instruments (Bruker) and analyzed with MestReNova v.14.2.0-26256 (Mestrelab Research). The samples were dissolved in deuterated DMSO or chloroform, and chemical shifts indicated relative to tetramethylsilane (TMS). The multiplicity of signals is indicated below with s = singlet, d = doublet, dd = doublet of doublets, t = triplet, q = quartet, and m = multiplet. Spectra were calibrated using the residual proton peaks or the ^13^C peaks of the solvent.

#### N-(4-(aminomethyl)benzyl)−8-(4-benzylpiperazin-1-yl)pyrimido[5,4-d]pyrimidin-4-amine (**1**)

Boc-protected intermediate **10** (0.092 mmol, 1.0 eq., 50 mg) was dissolved in HCl in EtOH (2.5 N, 3 mL) and the reaction mixture stirred for 16 h. After TLC indicated complete conversion, the solvent was removed under reduced pressure and the crude product purified by flash column chromatography on silica gel, using DCM/MeOH + TEA (3–8%) to afford 22 mg of **1** as white solid. Yield: 54%. ^1^H NMR (400 MHz, DMSO) δ 8.79 (t, *J* = 6.5 Hz, 1H), 8.48 (s, 1H), 8.36 (s, 1H), 7.37–7.13 (m, 11H), 4.66 (d, *J* = 6.4 Hz, 2H), 4.51–4.18 (m, 4H), 3.68–3.55 (m, 2H), 3.51 (s, 2H), 3.33–3.21 (m, 4H). ^13^C NMR (101 MHz, DMSO) δ 159.03, 157.58, 153.20, 152.49, 142.63, 137.76, 136.95, 134.28, 132.52, 128.97, 128.20, 127.09, 127.02, 126.95, 66.35, 61.88, 52.73, 45.34, 43.13. HR-MS m/z: 441.2492 [M + H]^+^(meas.); m/z: 441.2509 [M + H]^+^(calc.). HPLC t_ret_ = 2.975 min (method A).

#### 8-(4-benzylpiperazin-1-yl)-N-(3-methylbenzyl)pyrimido[5,4-d]pyrimidin-4-amine hydrochloride (**2**)

Intermediate **9** (0.30 mmol, 1.0 eq., 60 mg) was suspended in THF (dry, 20 mL) and DIPEA (0.90 mmol, 3.0 eq., 116 mg) added. Subsequently, 3-methylbenzylamine (0.33 mmol, 1.1 eq., 40 mg), dissolved in THF (dry, 5 mL), was added slowly at − 10 °C and the initial reaction mixture stirred for 1 h at the same temperature. After complete consumption of the starting material, the mixture was warmed up to RT and piperazine (0.46 mmol, 3.0 eq., 40 mg), dissolved in THF (dry, 5 mL), added. The mixture was stirred at RT for another hour, until HPLC indicated complete conversion. Afterwards, the mixture was diluted with ethyl acetate and washed 3 x with saturated sodium hydrogen carbonate solution. The solvent was removed under reduced pressure and the crude product purified by flash column chromatography on silica gel, using DCM/MeOH (2.5%) to afford 119 mg of **2** as brown oil. Yield: 93%. ^1^H NMR (200 MHz, CDCl_3_): δ 8.51 (s, 1H), 8.45 (s, 1H), 7.40 – 7.08 (m, 10H), 4.77 (d, J = 5.9 Hz, 2H), 4.50 (m, 4H), 3.58 (s, 2H), 2.67–2.59 (m, 4H), 2.36 (s, 3H). ^13^C NMR (50 MHz, CDCl_3_): δ 159.32, 158.24, 153.57, 152.50, 138.49, 137.80, 137.76, 134.73, 133.17, 129.32, 128.70, 128.57, 128.41, 128.38, 127.30, 124.90, 63.09, 53.45, 47.27, 44.72, 21.48. TLC-MS (ESI) m/z: 426.2 [M + H]^+^; 424.2 [M-H]^-^. HPLC t_ret_ = 5.935 min (method B).

The free base of **2** (0.28 mmol, 1.0 eq., 119 mg) was dissolved in HCl in EtOH (1.25 N, 5 mL) for precipitation as hydrochloride. The solvent was removed under reduced pressure to afford 129 mg of product **2** (x HCl) as white crystalline solid. Yield: 99%. ^1^H NMR (400 MHz, DMSO) δ 12.06 (s, 1H), 9.44–9.35 (m, 1H), 8.66 (s, 1H), 8.44 (s, 1H), 7.65 (dd, *J* = 6.1, 2.9 Hz, 2H), 7.48–7.39 (m, 3H), 7.22–7.10 (m, 3H), 7.04 (d, *J* = 7.1 Hz, 1H), 5.66–5.51 (m, 2H), 4.72 (d, *J* = 6.1 Hz, 2H), 4.33 (s, 2H), 3.78 (t, *J* = 12.7 Hz, 2H), 3.50–3.35 (m, 2H), 3.28–3.10 (m, 2H), 2.25 (s, 3H). ^13^C NMR (101 MHz, DMSO) δ 157.80, 157.70, 152.47, 151.98, 138.27, 137.33, 132.95, 132.21, 131.47, 129.41, 129.35, 128.68, 128.16, 127.86, 127.56, 124.38, 58.56, 50.18, 43.66, 43.66, 20.95. HR-MS m/z: 426.2393 [M + H]^+^(meas.); m/z: 426.2399z [M + H]^+^(calc.). HPLC t_ret_ = 5.989 min (method B).

#### 8-(4-benzylpiperazin-1-yl)-N-(3-((methylamino)methyl)benzyl)pyrimido[5,4-d]pyrimidin-4-amine (**3**)

Intermediate **14** (0.15 mmol, 1.0 eq., 84 mg) was dissolved in HCl in EtOH (2.5 N, 3 mL) and the reaction mixture was stirred at RT until TLC indicated complete conversion. Afterwards, the solvent was removed under reduced pressure and the resulting precipitate was purified by flash column chromatography on silica gel, using a gradient of DCM/MeOH + 2 N NH_3_ (5–7.5%). The free base was then taken into MeOH and precipitated, using HCl in EtOH to afford 39 mg of product **3** (x 2 HCl) as beige solid. Yield: 48%. ^1^H NMR (400 MHz, DMSO) δ 11.87 (s, 1H), 9.38 – 9.27 (m, 2H), 9.14 (t, *J* = 6.2 Hz, 1H), 8.62 (s, 1H), 8.41 (s, 1H), 7.65 (dd, *J* = 6.2, 3.0 Hz, 2H), 7.48–7.42 (m, 5H), 7.36 (d, *J* = 4.7 Hz, 2H), 5.76–5.46 (m, 2H), 4.75 (d, *J* = 6.1 Hz, 2H), 4.34 (s, 2H), 4.05 (t, *J* = 5.8 Hz, 2H), 3.72–3.66 (m, 2H), 3.48–3.37 (m, 2H), 3.26–3.14 (m, 2H), 2.48 (m, *J* = 5.6 Hz, 3H). ^13^C NMR (50 MHz, DMSO-d6): δ 158.7, 157.8, 152.9, 152.6, 139.3, 133.9, 132.5, 132.1, 131.5, 129.5, 129.4, 128.8, 128.7, 128.6, 128.4, 127.6, 58.6, 51.1, 50.3, 43.6, 43.4, 31.9. HR-MS m/z: 455.26670 [M + H]^+^(meas.); m/z: 455.26662 [M + H]^+^(calc.). HPLC t_ret_ = 1.355 min (method B).

#### 8-(4-benzylpiperazin-1-yl)-N-(3-((dimethylamino)methyl)benzyl)pyrimido[5,4-d]pyrimidin-4-amine (**4**)

Intermediate **9** (0.25 mmol, 1.0 eq., 50 mg) was suspended in THF (dry, 20 mL) and triethylamine (0.50 mmol, 2.0 eq., 50 mg) was added. Subsequently, **15** (0.27 mmol, 1.1 eq., 44 mg), dissolved in THF (dry, 5 mL), was added slowly at −10 °C and the initial reaction mixture stirred for 1 h at the same temperature. After complete consumption of the starting material, it was warmed up to RT and 1-benzylpiperazine (0.55 mmol, 2.0 eq., 97 mg), dissolved in THF (dry, 5 mL), added. The mixture was stirred at RT for another hour, until HPLC indicated complete conversion. Afterwards, the mixture was diluted with ethyl acetate and washed 3 x with saturated sodium hydrogen carbonate solution. The solvent was removed under reduced pressure and the crude product purified by flash column chromatography on silica gel, using a gradient of DCM/MeOH + 2 N NH_3_ (3.5–8.5% and 5–8.5%). The free base was then taken into MeOH and precipitated, using HCl in EtOH to afford 50 mg of product **4** (x 2 HCl) as white solid. Yield: 43%. ^1^H NMR (400 MHz, DMSO) δ 11.67 (s, 1H), 10.71 – 10.60 (m, 1H), 9.06 (t, *J* = 6.3 Hz, 1H), 8.61 (s, 1H), 8.41 (s, 1H), 7.63 (dd, *J* = 6.7, 3.0 Hz, 2H), 7.47 (dd, *J* = 7.8, 4.6 Hz, 5H), 7.40 (d, *J* = 6.5 Hz, 2H), 6.10–5.25 (m, 2H), 4.75 (d, *J* = 6.3 Hz, 2H), 4.33 (d, *J* = 4.8 Hz, 2H), 4.23 (d, *J* = 5.4 Hz, 2H), 3.70 (t, *J* = 13.1 Hz, 2H), 3.47–3.39 (m, 2H), 3.25–3.12 (m, 2H), 2.65 (d, *J* = 4.9 Hz, 6H). ^13^C NMR (101 MHz, DMSO-d6): δ 158.7, 157.7, 152.8, 152.5, 139.3, 133.9, 132.5, 131.4, 130.3, 129.9, 129.5, 129.4, 129.3, 128.7, 128.7, 128.1, 59.2, 58.6, 50.2, 43.5, 43.3, 41.4. HR-MS m/z: 469.28265 [M + H]^+^(meas.); m/z: 469.28227 [M + H]^+^(calc.). HPLC t_ret_ = 1.328 min (method B).

#### N-(3-(aminomethyl)benzyl)−8-(4-methylpiperazin-1-yl)pyrimido[5,4-d]pyrimidin-4-amine (**5**)

Boc-protected intermediate **17** (0.15 mmol, 1.0 eq., 72 mg) was dissolved in HCl in EtOH (2.5 N, 3 mL) and the reaction mixture stirred at RT for 65 h, until HPLC indicated complete conversion. Afterwards, the solvent was removed under reduced pressure and the resulting precipitate free-based and purified by flash column chromatography on silica gel, using a gradient of DCM/MeOH + 2 N NH_3_ (0–10%). The free base was then taken into MeOH and precipitated using HCl in EtOH to afford 28 mg of product **5** (x 2 HCl) as white solid. Yield: 38%. ^1^H NMR (400 MHz, DMSO) δ 11.69 (s, 1H), 9.17 (t, *J* = 6.3 Hz, 1H), 8.64 (s, 1H), 8.57–8.45 (m, 3H), 8.43 (s, 1H), 7.46–7.30 (m, 4H), 5.74–5.48 (m, 2H), 4.75 (d, *J* = 6.2 Hz, 2H), 3.96 (q, *J* = 5.8 Hz, 2H), 3.68 (t, *J* = 12.4 Hz, 2H), 3.57–3.48 (m, 2H), 3.26–3.10 (m, 2H), 2.75 (d, *J* = 4.4 Hz, 3H). ^13^C NMR (101 MHz, DMSO-d6): δ 159.2, 158.3, 153.3, 153.2, 139.7, 134.6, 133.1, 129.1, 128.2, 127.9, 127.7, 127.7, 52.5, 44.2, 43.9, 42.6, 42.4. HR-MS m/z: 365.21989 [M + H]^+^(meas.); m/z: 365.21967 [M + H]^+^(calc.). HPLC t_ret_ = 1.040 min (method B).

#### N-(3-(aminomethyl)benzyl)−8-(4-(pent-4-yn-1-yl)piperazin-1-yl)pyrimido[5,4-d]pyrimidin-4-amine (**6**)

Boc-protected intermediate **19** (0.12 mmol, 1.0 eq., 60 mg) was dissolved in HCl in EtOH (2.5 N, 2 mL) and the reaction mixture stirred at RT for 16 h, until HPLC indicated complete conversion. Afterwards, the solvent was removed under reduced pressure and the resulting precipitate was purified via flash column chromatography on silica gel using a gradient of DCM/MeOH + 0.5% TEA (5–10%). The free base was then taken into MeOH and precipitated using HCl in EtOH to afford 49 mg of product **6** (x 2 HCl) as a yellow solid. Yield: 80%. ^1^H NMR (400 MHz, DMSO) δ 11.67 (s, 1H), 9.07 (t, *J* = 6.3 Hz, 1H), 8.62 (s, 1H), 8.43 (d, *J* = 5.4 Hz, 4H), 7.43 (s, 1H), 7.39–7.29 (m, 3H), 5.80–5.49 (m, 2H), 4.74 (d, *J* = 6.2 Hz, 2H), 4.00–3.92 (m, 2H), 3.73 (t, *J* = 13.2 Hz, 2H), 3.62 (d, *J* = 11.9 Hz, 2H), 3.21–3.05 (m, 4H), 2.91 (s, 1H), 2.34–2.26 (m, 2H), 1.96 (t, *J* = 8.5 Hz, 2H). ^13^C NMR (101 MHz, DMSO) δ 158.61, 157.36, 152.68, 152.50, 139.00, 133.79, 133.73, 132.33, 128.27, 127.29, 127.05, 126.82, 82.49, 71.96, 54.43, 50.39, 43.22, 42.96, 41.81, 21.80, 15.05. HR-MS m/z: 417.25178 [M + H]^+^(meas.); m/z: 417.25097 [M + H]^+^(calc.). HPLC t_ret_ = 1.187 min (method A).

#### N-(3-(aminomethyl)benzyl)−8-(4-(prop-2-yn-1-yl)piperazin-1-yl)pyrimido[5,4-d]pyrimidin-4-amine (**8**)

Boc-protected intermediate **20** (0.13 mmol, 1.0 eq., 65 mg) was dissolved in HCl in EtOH (2.5 N, 2 mL) and the reaction mixture was stirred at RT for 16 h, until HPLC indicated complete conversion. Afterwards, the solvent was removed under reduced pressure and the resulting precipitate was purified via flash column chromatography on silica gel using a gradient of DCM/MeOH + 0.5% TEA (5–10%) to afford 36 mg of **8** (x 2 HCl) as yellow solid. Yield: 47%. ^1^H NMR (400 MHz, DMSO) δ 12.43 (s, 1H), 9.17 (d, *J* = 6.4 Hz, 1H), 8.65 (s, 1H), 8.52 (s, 3H), 8.44 (s, 1H), 7.44 (s, 1H), 7.42–7.29 (m, 3H), 5.96–5.21 (m, 2H), 4.79–4.68 (m, 2H), 4.13 (s, 2H), 3.96 (t, *J* = 5.6 Hz, 2H), 3.86 (s, 1H), 3.81–3.64 (m, 2H), 3.60–3.39 (m, 2H), 3.36–3.00 (m, 2H).^13^C NMR (101 MHz, DMSO) δ 158.56, 157.36, 152.58, 152.51, 138.88, 133.77, 133.67, 132.24, 128.14, 127.18, 126.96, 126.66, 81.05, 72.62, 49.60, 43.86, 43.09, 42.83, 41.68. HR-MS m/z: 389.22037 [M + H]^+^(meas.); m/z: 389.21967 [M + H]^+^(calc.). HPLC t_ret_ = 3.325 min (method A).

#### 4,8-dichloropyrimido[5,4-d]pyrimidine (**9**)

Intermediate **9** was synthesized according to patent literature, WO2010026262 A1.

#### tert-butyl (4-(((8-(piperazin-1-yl)pyrimido[5,4-d]pyrimidin-4-yl)amino)methyl)benzyl) carbamate (**10**)

Dichloride **9** (0.40 mmol, 1.0 eq., 80 mg) was weighed into a round bottom flask which was put under nitrogen atmosphere. It was then suspended in THF (dry, 25 mL) and DIPEA (0.80 mmol, 2.0 eq., 103 mg) was added. Subsequently, *tert*-butyl (4-(aminomethyl)benzyl)carbamate (0.44 mmol, 1.1 eq., 103 mg), dissolved in THF, was added slowly at −10 °C and the initial reaction mixture was stirred for 1 h at the same temperature. After complete consumption of the starting material, the reaction mixture was warmed up to RT and 1-benzylpiperazine (1.20 mmol, 3.0 eq., 212 mg), dissolved in THF (dry, 5 mL), added. The reaction mixture was stirred at RT for another 2 h, until HPLC indicated complete conversion. Afterwards, it was diluted with ethyl acetate and washed three times with saturated sodium hydrogen carbonate solution. The solvent was removed under reduced pressure and the crude product purified via flash column chromatography on silica gel using a gradient of DCM/MeOH (0–5%) to afford 199 mg of product **10**. Yield: 93%. ^1^H NMR (400 MHz, CDCl_3_) δ 8.38 (s, 1H), 8.33 (s, 1H), 7.27–7.13 (m, 10H), 4.83 (s, 1H), 4.67 (d, *J* = 6.0 Hz, 2H), 4.45–4.28 (m, 4H), 4.21 (d, *J* = 6.0 Hz, 2H), 3.47 (s, 2H), 2.51 (t, *J* = 5.0 Hz, 4H), 1.37 (s, 9H). ^13^C NMR (101 MHz, CDCl_3_) δ 159.38, 158.30, 155.99, 153.64, 152.49, 138.52, 137.79, 137.08, 134.75, 133.22, 129.37, 128.43, 128.15, 127.98, 127.35, 79.65, 77.36, 63.14, 53.50, 47.34, 44.47, 28.53. TLC-MS (ESI) m/z: 541.7 [M + H]^+^. HPLC t_ret_ = 9.947 min (method A).

#### 3-((methylamino)methyl)benzonitrile (**11**)

To a solution of methylamine (40%, aq., 102 mmol, 20.0 eq., 9 mL) in ACN (HPLC-grade, 10 mL), 3-(bromomethyl)benzonitrile (5.1 mmol, 1.0 eq., 1.0 g), dissolved in ACN (HPLC-grade, 10 mL), was added dropwise over 20 min at RT, stirring. The reaction mixture was stirred for 30 min. Afterwards, the solvent was removed under reduced pressure to obtain 1.2 g of crude **11** as a white solid. Yield: quant. ^1^H NMR (200 MHz, DMSO): δ 8.02 (s, 1H), 7.88 (d, *J* = 7.7 Hz, 2H), 7.63 (d, *J* = 8.1 Hz, 1H), 4.20 (s, 2H), 2.53 (s, 3H), 2.35 (s, 1H). ^13^C NMR (50 MHz, DMSO): δ 134.88, 134.08, 133.61, 132.50, 129.89, 118.43, 111.44, 50.36, 32.28. GC-MS (EI): m/z [M]: 145. HPLC t_ret_ = 1.324 min (method B).

#### tert-butyl (3-cyanobenzyl)(methyl)carbamate (**12**)

The crude material of intermediate **11** (4.40 mmol, 1.0 eq., 1.0 g) was suspended in DCM (dry, 7 mL) in a round bottom flask which was put under nitrogen atmosphere. Then, the suspension was cooled to 0 °C at which triethylamine (8.81 mmol, 2.0 eq., 0.9 g) was added followed by di-*tert*-butyl dicarbonate (4.84 mmol, 1.1 eq., 1.1 g), dissolved in DCM (dry, 4 mL). The reaction mixture was warmed up to RT and stirred for 30 min. Afterwards, the solvent was removed under reduced pressure and the resulting precipitate taken into DCM and washed with saturated sodium hydrogen carbonate solution. The aqueous phase was subsequently extracted with DCM three times. After the removal of the solvent, the crude material was purified via flash column chromatography on silica gel using a gradient of PE/EtOAc (20–33%) to afford 0.8 g of **12** as yellow oil. Yield: 77%. ^1^H NMR (200 MHz, DMSO): δ 7.80–7.70 (m, 1H), 7.68–7.63 (m, 1H), 7.61–7.53 (m, 2H), 4.43 (s, 2H), 2.81 (s, 3H), 1.50–1.27 (m, 9H). ^13^C NMR (50 MHz, DMSO) δ 140.08, 132.02, 130.91, 130.70, 129.77, 118.70, 111.43, 79.04, 34.15, 28.20, 27.95. TLC-MS (ESI) m/z: 191.8 [M + H]^+^ without *t*Bu. HPLC t_ret_ = 7.783 min (method B).

#### tert-butyl (3-(aminomethyl)benzyl)(methyl)carbamate (**13**)

To a flask containing MeOH (HPLC-grade, 20 mL) and acetic acid (3.25 mmol, 2.0 eq., 195 mg), intermediate **12** (1.62 mmol, 1.0 eq., 400 mg) and palladium on carbon (10 wt.%, 0.32 mmol, 0.2 eq., 80 mg) were added. The flask was then sealed with a septum and stirred under hydrogen atmosphere at RT for 6 h, until TLC indicated complete conversion. Afterwards, the reaction mixture was filtered using a Celite® pad and washed with MeOH. The solvent was removed from the resulting filtrate under reduced pressure and the precipitate was purified via flash column chromatography on silica gel using a gradient of DCM/MeOH + 2 N NH_3_ (5–10%) to afford 231 m g of product **13** as yellow resin. Yield: 57%. ^1^H NMR (200 MHz, DMSO) δ 7.34–7.15 (m, 3H), 7.08–6.99 (m, 1H), 4.35 (s, 2H), 3.70 (s, 2H), 2.74 (s, 3H), 1.99 (s, 2H), 1.42 (s, 9H). ^13^C NMR (50 MHz, DMSO) δ 155.06 (broad), 143.95, 137.87, 128.24, 126.06, 125.90, 125.13, 78.74, 51.73 + 51.07 (rotameric), 45.44, 33.75, 28.05. GC-MS: m/z [M]: 194; t_ret_ = 4.389 min (method B).

#### tert-butyl (3-(((8-(4-benzylpiperazin-1-yl)pyrimido[5,4-d]pyrimidin-4-yl)amino)methyl)benzyl)(methyl)carbamate (**14**)

Dichloride **9** (0.30 mmol, 1.0 eq., 60 mg) was suspended in THF (dry, 20 mL) and triethylamine (0.60 mmol, 2.0 eq., 61 mg) was added. Subsequently, compound **13** (0.33 mmol, 1.1 eq., 82 mg), dissolved in THF (dry, 5 mL), was added slowly at − 10 °C and the initial reaction mixture was stirred at the same temperature for 1 h. After complete consumption of the starting material, it was warmed to RT and 1-benzylpiperazine (0.60 mmol, 2.0 eq., 106 mg), dissolved in THF (dry, 5 mL), was added. The reaction mixture was stirred at RT for 4 h, until HPLC indicated complete conversion. Afterwards, it was diluted with ethyl acetate and washed three times with saturated sodium hydrogen carbonate solution. The solvent was removed under reduced pressure and the crude product purified via flash column chromatography on silica gel using DCM/MeOH (2.5%) to afford 84 mg of product **14** as yellow oil. Yield: 50%. ^1^H NMR (200 MHz, CDCl_3_) δ 8.40 (s, 1H), 8.35 (s, 1H), 7.28–6.90 (m, 10H), 4.71 (d, *J* = 6.0 Hz, 2H), 4.39 (s, 4H), 4.34 (s, 2H), 3.49 (s, 2H), 2.73 (s, 3H), 2.60–2.43 (m, 4H), 1.38 (s, 9H). ^13^C NMR (50 MHz, CDCl_3_) δ 159.34, 158.20, 153.57, 152.42, 138.76, 138.28, 137.66, 134.68, 133.16, 129.29, 128.99, 128.35, 127.29, 126.63, 79.70, 63.04, 53.41, 47.22, 44.57, 34.03, 29.69, 28.48. TLC-MS (ESI) m/z: 553.4 [M-H]^-^. HPLC t_ret_ = 6.965 min (method B).

#### 1-(3-(aminomethyl)phenyl)-N,N-dimethylmethanamine (**15**)

LiAlH_4_ solution (2 M in THF, 1.50 mmol, 0.97 eq., 2.3 mL) was added dropwise to a flask containing 3-((dimethylamino)methyl)benzonitrile (1.55 mmol, 1.0 eq., 250 mg) in THF (dry, 5 mL) at 0 °C under nitrogen atmosphere. After 15 min of stirring at 0 °C, the reaction mixture was warmed to RT and stirred for another 40 min. Afterwards, the reaction was quenched using water and NaOH solution (2 N, aq.). The resulting suspension was diluted with ethyl acetate, filtered and washed with ethyl acetate. The solvent of the filtrate was then removed under reduced pressure and the precipitate purified via flash column chromatography on silica gel using DCM/MeOH + 2 N NH_3_ (10%) to afford 222 mg of **15** as yellow oil. Yield: 87%. ^1^H NMR (200 MHz, CDCl_3_) δ 7.38–7.11 (m, 4H), 3.86 (s, 2H), 3.42 (s, 2H), 2.25 (s, 6H), 1.78 (s, 2H). ^13^C NMR (50 MHz, CDCl_3_) δ 142.66, 138.50, 127.72, 127.04, 126.88, 125.12, 63.68, 45.72, 44.73. GC-MS: m/z [M]: 164; t_ret_ = 2.314 min (method B).

#### tert-butyl (3-(aminomethyl)benzyl)carbamate (**16**)

To a solution of *m*-xylyl-diamine (90.30 mmol, 4.0 eq., 12.3 g) in dioxane (dry, 60 mL) di-*tert*-butyl decarbonate (22.60 mmol, 1.0 eq., 4.9 g) in dioxane (dry, 60 mL) was added through a dripping funnel over 4 h at 105 °C (reflux). After complete addition, the reaction refluxing was continued for 18 h at 105 °C. The solvent was then evaporated. the resulting precipitate was taken into water (150 mL), sonicated for 10 min and filtered. Subsequently, the aqueous filtrate was extracted with DCM (100 mL) and the organic layer was washed with water (6 × 100 mL). Following the removal of the solvent under reduced pressure, the crude product was dried over in an oil pump vacuum to yield 4.7 g of product **16** as yellow oil. Yield: 87%. ^1^H NMR (200 MHz, DMSO) δ 7.35 (s, 1H), 7.29–7.12 (m, 3H), 7.06 (d, *J* = 7.0 Hz, 1H), 4.10 (d, *J* = 6.2 Hz, 2H), 3.69 (s, 2H), 3.30 (s, 2H), 1.39 (s, 9H). ^13^C NMR (50 MHz, DMSO) δ 155.81, 144.11, 139.90, 127.97, 125.66, 125.35, 124.77, 77.72, 45.68, 43.46, 28.25. HPLC t_ret_ = 10.153 min (method B).

#### tert-butyl (3-(((8-(4-methylpiperazin-1-yl)pyrimido[5,4-d]pyrimidin-4-yl)amino)methyl)benzyl) carbamate (**17**)

Dichloride **9** (0.20 mmol, 1.0 eq., 40 mg) was suspended in THF (dry, 15 mL) and DIPEA (0.50 mmol, 2.0 eq., 52 mg) was added. Subsequently, intermediate **16** (0.22 mmol, 1.1 eq., 52 mg), dissolved in THF (dry, 5 mL), was added slowly at −10 °C and the initial reaction mixture was stirred for 1 h at the same temperature. After complete consumption of the starting material, it was warmed to RT and 1-methylpiperazine (0.40 mmol, 2.0 eq., 40 mg), dissolved in THF (dry, 5 mL), added. The reaction mixture was stirred at RT for 3 h, until HPLC indicated complete conversion. Afterwards, it was diluted with ethyl acetate and washed three times with saturated sodium hydrogen carbonate solution. The solvent was removed under reduced pressure and the crude product purified via flash column chromatography on silica gel using a gradient of DCM/MeOH (3.5 − 8.5%) to afford 90 mg of **17**. Yield: 98%. ^1^H NMR (400 MHz, CDCl_3_) δ 8.48 (s, 1H), 8.44 (s, 1H), 7.34–7.27 (m, 4H), 7.21 (d, *J* = 7.2 Hz, 1H), 4.91–4.83 (m, 1H), 4.77 (d, *J* = 5.9 Hz, 2H), 4.62–4.43 (m, 4H), 4.29 (d, *J* = 5.9 Hz, 2H), 2.64 (t, *J* = 5.1 Hz, 4H), 2.39 (s, 3H), 1.44 (s, 9H). ^13^C NMR (50 MHz, CDCl_3_) δ 159.33, 158.28, 155.98, 153.54, 152.51, 139.66, 138.31, 134.69, 133.17, 129.06, 126.73, 79.54, 55.25, 53.49, 50.52, 46.97, 45.88, 44.55, 28.45. TLC-MS (ESI) m/z: 463.5 [M-H]^-^; m/z: 465.6 [M + H]^+^. HPLC t_ret_ = 5.199 min (method B).

#### tert-butyl (3-(((8-(piperazin-1-yl)pyrimido[5,4-d]pyrimidin-4-yl)amino)methyl)benzyl) carbamate (**18**)

Dichloride **9** (1.50 mmol, 1.0 eq., 302 mg) was suspended in THF (dry, 100 mL) and DIPEA (3.00 mmol, 2.0 eq., 338 mg) was added. Subsequently, intermediate **16** (1.65 mmol, 1.1 eq., 390 mg), dissolved in THF (dry, 50 mL), was added slowly at −10 °C and the initial reaction mixture was stirred for 1 h at the same temperature. After complete consumption of the starting material, it was warmed to RT and piperazine (4.51 mmol, 3.0 eq., 388 mg), dissolved in THF (dry, 20 mL), added. The reaction mixture was stirred at RT for 20 min, until HPLC indicated complete conversion. Afterwards, it was diluted with ethyl acetate and washed three times with saturated sodium hydrogen carbonate solution. The solvent was removed under reduced pressure and the crude product purified via flash column chromatography on silica gel using a gradient of DCM/MeOH + 2 N NH_3_ (0 − 10%) to afford 486 mg of **18** as yellow oil. Yield: 72%. ^1^H NMR (200 MHz, CDCl_3_) δ 8.47 (s, 1H), 8.41 (s, 1H), 7.29 (s, 1H), 7.24 (d, *J* = 9.5 Hz, 4H), 4.96 (s, 1H), 4.76 (d, *J* = 5.9 Hz, 2H), 4.68 – 4.10 (m, 6H), 3.03 (d, *J* = 5.0 Hz, 2H), 2.35 (s, 1H), 1.43 (s, 9H), 1.34 – 1.00 (m, 2H).^13^C NMR (50 MHz, CDCl_3_) δ 159.39, 158.38, 155.98, 153.62, 152.51, 139.69, 138.36, 134.78, 133.23, 129.13, 126.86, 126.81, 79.63, 65.93, 48.52, 46.48, 46.36, 44.62, 28.50. TLC-MS (ESI) m/z: 449.3 [M-H]^-^. HPLC t_ret_ = 5.259 min (method B).

#### tert-butyl (3-(((8-(4-(pent-4-yn-1-yl)piperazin-1-yl)pyrimido[5,4-d]pyrimidin-4-yl)amino)methyl)benzyl) carbamate (**19**)

5-Chloropent-1-yne (0.22 mmol, 2.0 eq., 23 mg) and sodium iodide (0.22 mmol, 2.0 eq., 33 mg) were dissolved in acetone (dry, 2 mL) and stirred for 16 h. Afterwards, the suspension was added to a solution of intermediate **18** (0.11 mmol, 1.0 eq., 50 mg) and DIPEA (0.28 mmol, 2.5 eq., 36 mg) in DMF (dry, 1 mL). The reaction mixture was stirred for 8 h at 60 °C. After cooling down to RT, it was taken into ethyl acetate and washed three times with saturated sodium chloride solution. Then, the organic solvent was removed under reduced pressure and the crude product purified via flash column chromatography on silica gel using a gradient of DCM/MeOH (1–3%) to afford 42 mg of **19** as yellow oil. Yield: 74%. ^1^H NMR (400 MHz, CDCl_3_) δ 8.50–8.43 (m, 1H), 8.44–8.36 (m, 1H), 7.28 (dd, *J* = 10.1, 5.1 Hz, 4H), 7.19 (t, *J* = 8.2 Hz, 1H), 4.86 (s, 1H), 4.76 (t, *J* = 7.4 Hz, 2H), 4.45 (s, 4H), 4.28 (s, 2H), 2.58 (p, *J* = 4.8 Hz, 4H), 2.52 – 2.42 (m, 2H), 2.25 (tt, *J* = 7.0, 3.6 Hz, 2H), 1.95 (dd, *J* = 6.9, 2.7 Hz, 1H), 1.74 (q, *J* = 8.0 Hz, 2H), 1.42 (d, *J* = 7.6 Hz, 9H). ^13^C NMR (101 MHz, CDCl_3_) δ 159.09, 157.99, 155.66, 153.35, 152.23, 139.37, 138.08, 134.46, 132.94, 128.87, 126.55, 83.86, 79.35, 77.05, 68.39, 56.98, 53.32, 46.97, 44.34, 29.51, 28.21, 25.47, 16.20. TLC-MS (ESI) m/z: 517.5 [M + H]^+^. HPLC t_ret_ = 9.280 min (method A).

#### tert-butyl (3-(((8-(4-(prop-2-yn-1-yl)piperazin-1-yl)pyrimido[5,4-d]pyrimidin-4-yl)amino)methyl)benzyl) carbamate (**20**)

To a solution of intermediate **18** (0.13 mmol, 1.0 eq., 60 mg) and DIPEA (0.27 mmol, 2.0 eq., 34 mg) in THF (dry, 2 mL) propargyl bromide (80% in toluene, 0.53 mmol, 4.0 eq., 70 mg) was added dropwise whilst stirring. The reaction mixture was stirred at RT for 3 h, then taken into ethyl acetate and washed with saturated sodium carbonate solution. Afterwards, the solvent of the organic layer was removed under reduced pressure and the crude product purified via flash column chromatography on silica gel using a gradient of DCM/MeOH + 0.5% TEA (0 − 5%) to afford 63 mg of **20** as yellow solid. Yield: 97%. ^1^H NMR (400 MHz, CDCl_3_) δ 8.48 (s, 1H), 8.43 (s, 1H), 7.32–7.27 (m, 4H), 7.21 (d, *J* = 7.4 Hz, 1H), 4.87 (s, 1H), 4.77 (d, *J* = 6.0 Hz, 2H), 4.51 (s, 4H), 4.30 (d, *J* = 5.9 Hz, 2H), 3.37 (d, *J* = 2.5 Hz, 2H), 2.72 (t, *J* = 5.1 Hz, 4H), 2.25 (s, 1H), 1.44 (s, 9H). ^13^C NMR (101 MHz, CDCl_3_) δ 159.42, 158.34, 156.00, 153.66, 152.59, 139.72, 138.38, 134.81, 133.25, 129.19, 126.92, 126.87, 79.66, 78.47, 77.36, 76.97, 73.71, 52.23, 47.20, 46.95, 44.68, 28.53. TLC-MS (ESI) m/z: 511.5 [M+Na]^+^; m/z: 487.4. [M-H]^-^. HPLC t_ret_ = 10.717 min (method A).

### Statistics and reproducibility

Statistical significance was determined by unpaired, two-tailed *t*-tests using Prism v.9.3.1 (GraphPad) with *p* > 0.05 = ns (not significant), *p ≤ 0.05, **p ≤ 0.01, ***p ≤ 0.001, ****p ≤ 0.0001. Representative results of at least three independent experiments are shown, except for the comparative SEC analyses of BI8626 and BI8622 ubiquitination by different HECT domains, which represent two independent replicates (Fig. 3e; Supplementary Fig. 12d).

### Reporting summary

Further information on research design is available in the Nature Portfolio Reporting Summary linked to this article.

## Supplementary information

Supplementary Information

Description of Additional Supplementary Files

Supplementary Data 1

Supplementary Data 2

Reporting Summary

Transparent Peer Review file

## Source data

Source Data

## Data Availability

The HDX-MS data are available in Supplementary Data 1, global and diGly proteomic data in Supplementary Data 2. Additionally, the HDX-MS data (accession code: PXD066563), the global and diGly proteomic data (accession code: PXD067088), and other MS data (accession code: PXD063550 [http://proteomecentral.proteomexchange.org/cgi/GetDataset?ID=PXD066550]) have been uploaded to the ProteomeXchange Consortium via the PRIDE partner repository^70^. The MD force field parameters for BI8626, MD-derived models and the AF3-based dataset have been deposited to Zenodo (DOI: 10.5281/zenodo.15772692). Source data are provided with this paper.

## References

[1] Hershko, A. & Ciechanover, A. The ubiquitin system. *Annu. Rev. Biochem.* **67**, 425–479 (1998). 9759494 10.1146/annurev.biochem.67.1.425

[2] Dikic, I. & Schulman, B. A. An expanded lexicon for the ubiquitin code. *Nat. Rev. Mol. Cell Biol.* **24**, 273–287 (2023). 36284179 10.1038/s41580-022-00543-1 PMC9595094

[3] Dearlove, E. L. & Huang, D. T. Insights into non-proteinaceous ubiquitination. *Biochem. Soc. Trans*. **53**, 399–407 (2025). 10.1042/BST20253029 PMC12203941 40181599

[4] Wang, X. S. et al. The RBR E3 ubiquitin ligase HOIL-1 can ubiquitinate diverse non-protein substrates in vitro. *Life Sci. Alliance* **8**, e202503243 (2025). 40169258 10.26508/lsa.202503243 PMC11962058

[5] Kelsall, I. R. et al. HOIL-1 ubiquitin ligase activity targets unbranched glucosaccharides and is required to prevent polyglucosan accumulation. *EMBO J.* **41**, e109700 (2022). 35274759 10.15252/embj.2021109700 PMC9016349

[6] Yoshida, Y. et al. Sugar-mediated non-canonical ubiquitination impairs Nrf1/NFE2L1 activation. *Mol. Cell* **84**, 3115–3127.e11 (2024). 39116872 10.1016/j.molcel.2024.07.013

[7] Rehman, S. A. A. et al. Discovery and characterization of noncanonical E2-conjugating enzymes. *Sci. Adv.* **10**, eadh0123 (2024). 38536929 10.1126/sciadv.adh0123 PMC10971424

[8] Zhu, K. et al. Ubiquitylation of nucleic acids by DELTEX ubiquitin E3 ligase DTX3L. *EMBO Rep.* **25**, 4172–4189 (2024). 39242775 10.1038/s44319-024-00235-1 PMC11467253

[9] Dearlove, E. L. et al. DTX3L ubiquitin ligase ubiquitinates single-stranded nucleic acids. *eLife* **13**, P98070 (2024). 10.7554/eLife.98070 PMC11460948 39377462

[10] Yang, C.-S. et al. Ubiquitin modification by the E3 ligase/ADP-ribosyltransferase Dtx3L/Parp9. *Mol. Cell* **66**, 503–516.e5 (2017). 28525742 10.1016/j.molcel.2017.04.028 PMC5556935

[11] Ahmed, S. F. et al. DELTEX2 C-terminal domain recognizes and recruits ADP-ribosylated proteins for ubiquitination. *Sci. Adv.* **6**, eabc0629 (2020). 32937373 10.1126/sciadv.abc0629 PMC7442474

[12] Chatrin, C. et al. Structural insights into ADP-ribosylation of ubiquitin by Deltex family E3 ubiquitin ligases. *Sci. Adv.* **6**, eabc0418 (2020). 32948590 10.1126/sciadv.abc0418 PMC7500938

[13] Zhu, K. et al. DELTEX E3 ligases ubiquitylate ADP-ribosyl modification on protein substrates. *Sci. Adv.* **8**, eadd4253 (2022). 36197986 10.1126/sciadv.add4253 PMC7615817

[14] Zhu, K. et al. Ubiquitylation of nucleic acids by DELTEX ubiquitin E3 ligase DTX3L. *Nucleic Acids Res.* **2**, 801–815 (2024). 10.1038/s44319-024-00235-1 PMC11467253 39242775

[15] Pao, K.-C. et al. Activity-based E3 ligase profiling uncovers an E3 ligase with esterification activity. *Nature* **556**, 381–385 (2018). 29643511 10.1038/s41586-018-0026-1

[16] Swarnkar, A. et al. Determinants of chemoselectivity in ubiquitination by the J2 family of ubiquitin-conjugating enzymes. *EMBO J.* **43**, 6705–6739 (2024). 39533056 10.1038/s44318-024-00301-3 PMC11649903

[17] Wenzel, D. M., Lissounov, A., Brzovic, P. S. & Klevit, R. E. UBCH7 reactivity profile reveals parkin and HHARI to be RING/HECT hybrids. *Nature* **474**, 105–108 (2011). 21532592 10.1038/nature09966 PMC3444301

[18] Sakamaki, J. et al. Ubiquitination of phosphatidylethanolamine in organellar membranes. *Mol. Cell* **82**, 3677–3692.e11 (2022). 36044902 10.1016/j.molcel.2022.08.008

[19] Otten, E. G. et al. Ubiquitylation of lipopolysaccharide by RNF213 during bacterial infection. *Nature* **594**, 111–116 (2021). 34012115 10.1038/s41586-021-03566-4 PMC7610904

[20] Bejan, D. S., Lacoursiere, R. E., Pruneda, J. N. & Cohen, M. S. Ubiquitin is directly linked via an ester to protein-conjugated mono-ADP-ribose. *EMBO J.* **44**, 2211–2231 (2025). 40000907 10.1038/s44318-025-00391-7 PMC12000418

[21] Li, W. et al. Genome-wide and functional annotation of human E3 ubiquitin ligases identifies MULAN, a mitochondrial E3 that regulates the organelle’s dynamics and signaling. *PLoS One* **3**, e1487 (2008). 18213395 10.1371/journal.pone.0001487 PMC2198940

[22] Qiu, J. et al. Ubiquitination independent of E1 and E2 enzymes by bacterial effectors. *Nature* **533**, 120–124 (2016). 27049943 10.1038/nature17657 PMC4905768

[23] Bhogaraju, S. et al. Phosphoribosylation of ubiquitin promotes serine ubiquitination and impairs conventional ubiquitination. *Cell* **167**, 1636–1649.e13 (2016). 27912065 10.1016/j.cell.2016.11.019

[24] Akturk, A. et al. Mechanism of phosphoribosyl-ubiquitination mediated by a single Legionella effector. *Nature* **557**, 729–733 (2018). 29795346 10.1038/s41586-018-0147-6 PMC5980775

[25] Kalayil, S. et al. Insights into catalysis and function of phosphoribosyl-linked serine ubiquitination. *Nature* **557**, 734–738 (2018). 29795347 10.1038/s41586-018-0145-8 PMC5980784

[26] Kao, S.-H., Wu, H.-T. & Wu, K.-J. Ubiquitination by HUWE1 in tumorigenesis and beyond. *J. Biomed. Sci.* **25**, 67–15 (2018). 30176860 10.1186/s12929-018-0470-0 PMC6122628

[27] Chen, D., Gehringer, M. & Lorenz, S. Developing small-molecule inhibitors of HECT-Type ubiquitin ligases for therapeutic applications: challenges and opportunities. *ChemBioChem* **19**, 2123–2135 (2018). 30088849 10.1002/cbic.201800321 PMC6471174

[28] Kathman, S. G. et al. A small molecule that switches a ubiquitin ligase from a processive to a distributive enzymatic mechanism. *J. Am. Chem. Soc.* **137**, 12442–12445 (2015). 26371805 10.1021/jacs.5b06839 PMC4669213

[29] Maspero, E. et al. Structure-based design of potent and selective inhibitors of the HECT ligase NEDD4. *Commun Chem* **8**, 164 (2025). 40437006 10.1038/s42004-025-01557-4 PMC12119865

[30] Rothman, A. M. K. et al. Therapeutic potential of allosteric HECT E3 ligase inhibition. *Cell* **188**, 2603–2620 (2025). 40179885 10.1016/j.cell.2025.03.001 PMC12087876

[31] Peter, S. et al. Tumor cell-specific inhibition of MYC function using small molecule inhibitors of the HUWE1 ubiquitin ligase. *EMBO Mol. Med.* **12**, 1525–1541 (2014). 10.15252/emmm.201403927 PMC4287973 25253726

[32] Nair, R. M. et al. Reconstitution and structural analysis of a HECT ligase-ubiquitin complex via an activity-based probe. *ACS Chem. Biol.* **16**, 1615–1621 (2021). 34403242 10.1021/acschembio.1c00433 PMC8453484

[33] Hehl, L. A. et al. Structural snapshots along K48-linked ubiquitin chain formation by the HECT E3 UBR5. *Nat. Chem. Biol.* **20**, 190–200 (2023). 37620400 10.1038/s41589-023-01414-2 PMC10830417

[34] Maiwald, S. A. et al. TRIP12 structures reveal HECT E3 formation of K29 linkages and branched ubiquitin chains. *Nat. Struct. Mol. Biol*. Online ahead of print, 10.1038/s41594-025-01561-1 (2025). 10.1038/s41594-025-01561-1 PMC12440805 40419785

[35] Kamadurai, H. B. et al. Mechanism of ubiquitin ligation and lysine prioritization by a HECT E3. *eLife* **2**, e00828–e00828 (2013). 23936628 10.7554/eLife.00828 PMC3738095

[36] Zhong, Q., Gao, W., Du, F. & Wang, X. Mule/ARF-BP1, a BH3-only E3 ubiquitin ligase, catalyzes the polyubiquitination of Mcl-1 and regulates apoptosis. *Cell* **121**, 1085–1095 (2005). 15989957 10.1016/j.cell.2005.06.009

[37] Liu, Z., Oughtred, R. & Wing, S. S. Characterization of E3Histone, a novel testis ubiquitin protein ligase which ubiquitinates histones. *Mol. Cell. Biol.* **25**, 2819–2831 (2005). 15767685 10.1128/MCB.25.7.2819-2831.2005 PMC1061639

[38] Hao, Z. et al. K48-linked KLF4 ubiquitination by E3 ligase Mule controls T-cell proliferation and cell cycle progression. *Nat. Commun.* **8**, 14003 (2017). 28084302 10.1038/ncomms14003 PMC5241832

[39] Soucy, T. A. et al. An inhibitor of NEDD8-activating enzyme as a new approach to treat cancer. *Nature* **458**, 732–736 (2009). 19360080 10.1038/nature07884

[40] Monda, J. K. et al. HAPSTR1 localizes HUWE1 to the nucleus to limit stress signaling pathways. *Cell Rep.* **42**, 112496–112496 (2023). 37167062 10.1016/j.celrep.2023.112496 PMC10279472

[41] Mandemaker, I. K. et al. DNA damage-induced histone H1 ubiquitylation is mediated by HUWE1 and stimulates the RNF8-RNF168 pathway. *Sci. Rep.* **7**, 15353 (2017). 29127375 10.1038/s41598-017-15194-y PMC5681673

[42] Wertz, I. E. & Wang, X. From discovery to bedside: targeting the ubiquitin system. *Cell Chem. Biol.* **26**, 156–177 (2019). 30554913 10.1016/j.chembiol.2018.10.022

[43] Raza, S., Safyan, R. A. & Lentzsch, S. Immunomodulatory drugs (IMiDs) in multiple myeloma. *Curr. Cancer Drug Targets* **17**, 846–857 (2015). 10.2174/1568009617666170214104426 28201976

[44] Chirnomas, D., Hornberger, K. R. & Crews, C. M. Protein degraders enter the clinic—a new approach to cancer therapy. *Nat. Rev. Clin. Oncol.* **20**, 265–278 (2023). 36781982 10.1038/s41571-023-00736-3 PMC11698446

[45] Li, W. et al. Highly specific intracellular ubiquitination of a small molecule. *bioRxiv* 2024.01.26.577493, 10.1101/2024.01.26.577493 (2024).

[46] Sander, B., Xu, W., Eilers, M., Popov, N. & Lorenz, S. A conformational switch regulates the ubiquitin ligase HUWE1. *eLife* **6**, e21036 (2017). 28193319 10.7554/eLife.21036 PMC5308896

[47] Ries, L. K. et al. Analysis of ubiquitin recognition by the HECT ligase E6AP provides insight into its linkage specificity. *J. Biol. Chem.* **294**, 6113–6129 (2019). 30737286 10.1074/jbc.RA118.007014 PMC6463701

[48] Düring, J. et al. Structural mechanisms of autoinhibition and substrate recognition by the ubiquitin ligase HACE1. *Nat. Struct. Mol. Biol.* **31**, 364–377 (2024). 38332367 10.1038/s41594-023-01203-4 PMC10873202

[49] Gersch, M. et al. Distinct USP25 and USP28 Oligomerization States Regulate Deubiquitinating Activity. *Mol. Cell* **74**, 436–451.e7 (2019). 30926242 10.1016/j.molcel.2019.02.030 PMC6509359

[50] Wickliffe, K. E., Lorenz, S., Wemmer, D. E., Kuriyan, J. & Rape, M. The mechanism of linkage-specific ubiquitin chain elongation by a single-subunit E2. *Cell* **144**, 769–781 (2011). 21376237 10.1016/j.cell.2011.01.035 PMC3072108

[51] Hunkeler, M. et al. Solenoid architecture of HUWE1 contributes to ligase activity and substrate recognition. *Mol. Cell* **81**, 3468–3480.e7 (2020). 10.1016/j.molcel.2021.06.032 PMC8476073 34314700

[52] Kurokawa, M. et al. A network of substrates of the E3 ubiquitin ligases MDM2 and HUWE1 control apoptosis independently of p53. *Sci. Signal* **6**, ra32–ra32 (2013). 23652204 10.1126/scisignal.2003741 PMC3770270

[53] Kokic, G. et al. Structural basis for RNA polymerase II ubiquitylation and inactivation in transcription-coupled repair. *Nat. Struct. Mol. Biol.* **31**, 536–547 (2024). 38316879 10.1038/s41594-023-01207-0 PMC10948364

[54] Ye, Y. et al. Polyubiquitin binding and cross-reactivity in the USP domain deubiquitinase USP21. *EMBO Rep.* **12**, 350–357 (2011). 21399617 10.1038/embor.2011.17 PMC3077245

[55] Liess, A. K. L. et al. Dimerization regulates the human APC/C-associated ubiquitin-conjugating enzyme UBE2S. *Sci. Signal*. **13**, eaba8208 (2020). 10.1126/scisignal.aba8208 PMC7613103 33082289

[56] Cox, J. & Mann, M. MaxQuant enables high peptide identification rates, individualized p.p.b.-range mass accuracies and proteome-wide protein quantification. *Nat. Biotechnol.* **26**, 1367–1372 (2008). 19029910 10.1038/nbt.1511

[57] Cox, J. et al. Andromeda: a peptide search engine integrated into the MaxQuant environment. *J. Proteome Res.* **10**, 1794–1805 (2011). 21254760 10.1021/pr101065j

[58] Wales, T. E., Fadgen, K. E., Gerhardt, G. C. & Engen, J. R. High-speed and high-resolution UPLC separation at zero degrees celsius. *Anal. Chem.* **80**, 6815–6820 (2008). 18672890 10.1021/ac8008862 PMC2562353

[59] Geromanos, S. J. et al. The detection, correlation, and comparison of peptide precursor and product ions from data independent LC-MS with data dependant LC-MS/MS. *Proteomics* **9**, 1683–1695 (2009). 19294628 10.1002/pmic.200800562

[60] Li, G. et al. Database searching and accounting of multiplexed precursor and product ion spectra from the data independent analysis of simple and complex peptide mixtures. *Proteomics* **9**, 1696–1719 (2009). 19294629 10.1002/pmic.200800564

[61] Joiner, J. D. et al. HilE represses the activity of the Salmonella virulence regulator HilD via a mechanism distinct from that of intestinal long-chain fatty acids. *J. Biol. Chem.* **299**, 105387 (2023). 37890783 10.1016/j.jbc.2023.105387 PMC10696396

[62] Fiskin, E., Bionda, T., Dikic, I. & Behrends, C. Global analysis of host and bacterial ubiquitinome in response to salmonella typhimurium infection. *Mol. Cell* **62**, 967–981 (2016). 27211868 10.1016/j.molcel.2016.04.015

[63] Villén, J. & Gygi, S. P. The SCX/IMAC enrichment approach for global phosphorylation analysis by mass spectrometry. *Nat. Protoc.* **3**, 1630–1638 (2008). 18833199 10.1038/nprot.2008.150 PMC2728452

[64] Rappsilber, J., Ishihama, Y. & Mann, M. Stop and go extraction tips for matrix-assisted laser desorption/ionization, nanoelectrospray, and LC/MS sample pretreatment in proteomics. *Anal. Chem.* **75**, 663–670 (2003). 12585499 10.1021/ac026117i

[65] Tyanova, S. et al. The Perseus computational platform for comprehensive analysis of (prote)omics data. *Nat. Methods* **13**, 731–740 (2016). 27348712 10.1038/nmeth.3901

[66] Abramson, J. et al. Accurate structure prediction of biomolecular interactions with AlphaFold 3. *Nature* **630**, 493–500 (2024). 38718835 10.1038/s41586-024-07487-w PMC11168924

[67] Xu, Y., Anderson, D. E. & Ye, Y. The HECT domain ubiquitin ligase HUWE1 targets unassembled soluble proteins for degradation. *Cell Discov.* **2**, 16040 (2016). 27867533 10.1038/celldisc.2016.40 PMC5102030

[68] Cong, L. et al. Multiplex genome engineering using CRISPR/Cas systems. *Science* **339**, 819–823 (2013). 23287718 10.1126/science.1231143 PMC3795411

[69] Ran, F. A. et al. Genome engineering using the CRISPR-Cas9 system. *Nat. Protoc.* **8**, 2281–2308 (2013). 24157548 10.1038/nprot.2013.143 PMC3969860

[70] Perez-Riverol, Y. et al. The PRIDE database resources in 2022: a hub for mass spectrometry-based proteomics evidences. *Nucleic Acids Res.* **50**, D543–D552 (2021). 10.1093/nar/gkab1038 PMC8728295 34723319

