# Supplementary material for: Selective ubiquitination of drug-like small molecules by the ubiquitin ligase HUWE1
Source: Nat Commun. 2025 Sep 2;16:8182. doi: 10.1038/s41467-025-63442-x (PMC12405493; doi:10.1038/s41467-025-63442-x)
Supplement: Supplementary file 1 — Supplementary Information [file 41467_2025_63442_MOESM1_ESM.pdf]

SUPPLEMENTARY FIGURES

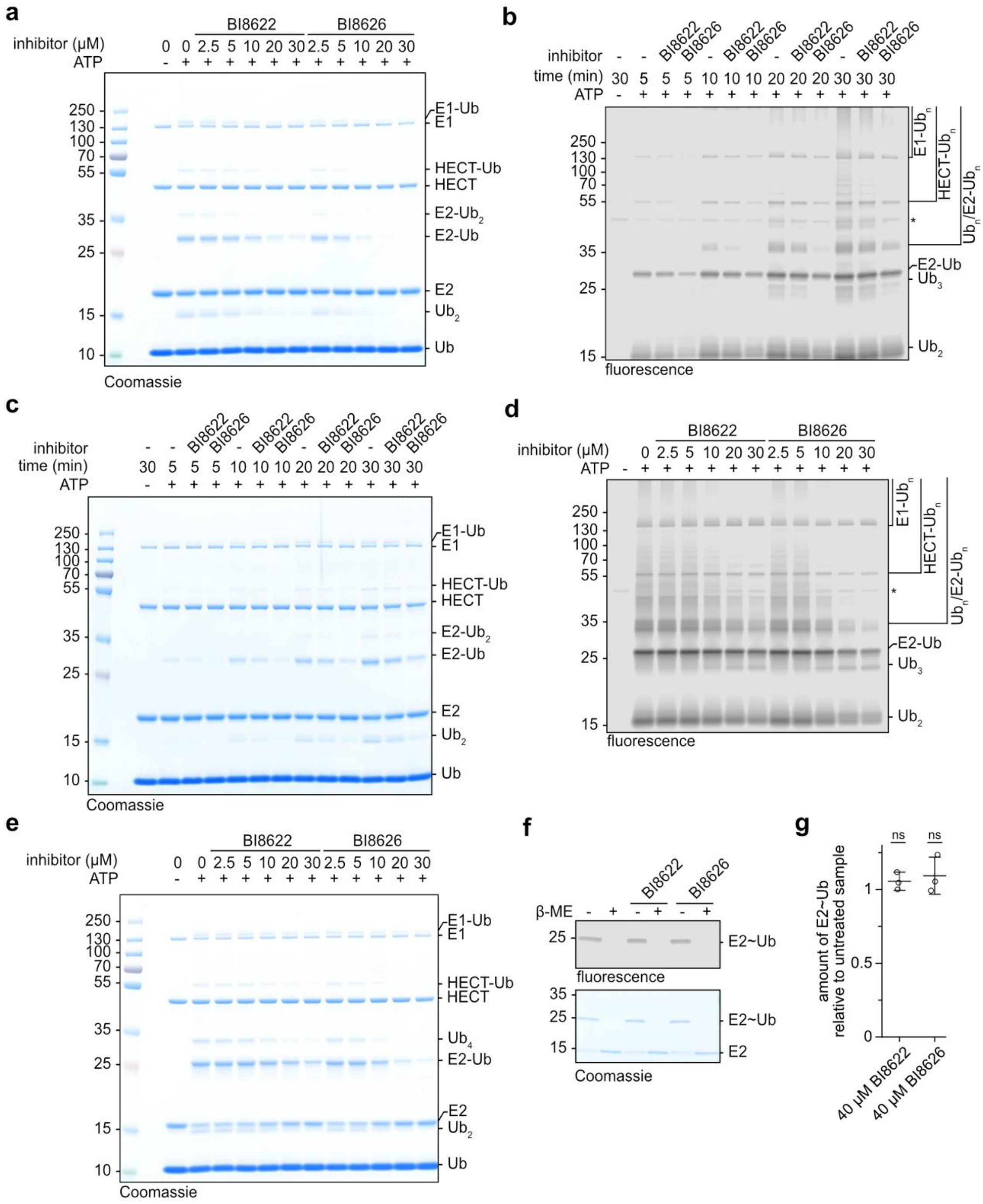

Supplementary Figure 1. Analyses of HUWE1^{HECT} inhibition by BI8622/BI8626
(a) Coomassie-stained gel from Fig. 1a. **(b)** Multi-turnover HUWE1^{HECT}-driven ubiquitination reactions, containing 20 µM BI8622 and BI8626, respectively, monitored over time via a fluorescent Ub tracer. **(c)** Protein input from (b), monitored by Coomassie staining. **(d)** Multi-turnover HUWE1^{HECT}-driven ubiquitination reactions with varying concentrations of BI8622 and BI8626, respectively, followed by a fluorescent Ub tracer. In contrast to Fig. 1a, in which UBE2L3 was used, the reactions here contain UBE2D3. **(e)** Protein input from (d), monitored by Coomassie staining. **(f)** Assay monitoring E1-driven Ub transfer to UBE2L3 in the presence of 40 µM BI8622 and BI8626, respectively, followed by a fluorescent Ub tracer (top); protein input (bottom). '~' indicates a thioester-linkage, verified by sensitivity to reducing agent (β–ME). **(g)** Quantification of assays as shown in (f). The amount of E2~Ub formed was normalized to that formed in inhibitor-free ('untreated') conditions. Data are represented as mean ± SD (n = 3 biological replicates). Statistical analyses with an unpaired, two-sided *t*-test; ns = not significant (compared to untreated conditions; p-value > 0.05). Source data are provided as a Source Data file.

a

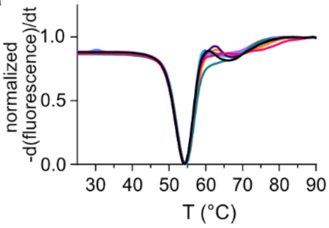

c

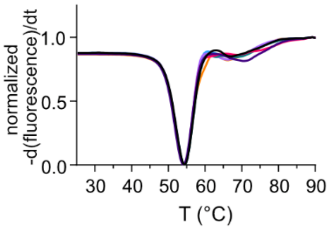

e

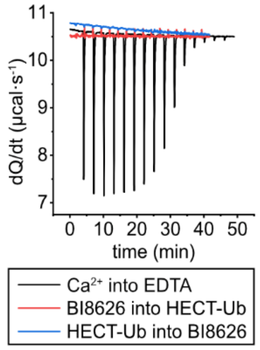

b

BI8626 (µM)	2.5	5	10	20	40	buffer	DMSO
T_m (HECT-Ub)	54.2	54.0	54.1	54.1	54.2	54.0	54.1
SD	0.1	0.2	0.1	0.3	0.6	0.2	0.2

d

BI8622 (µM)	2.5	5	10	20	40	buffer	DMSO
T_m (HECT-Ub)	54.1	54.2	54.0	54.1	54.0	54.0	54.1
SD	0.3	0.1	0.2	0.2	0.2	0.2	0.2

f

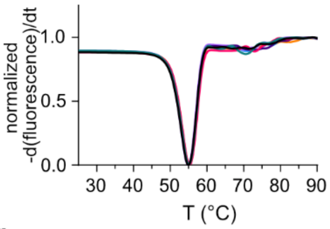

h

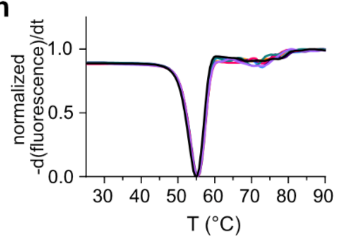

j

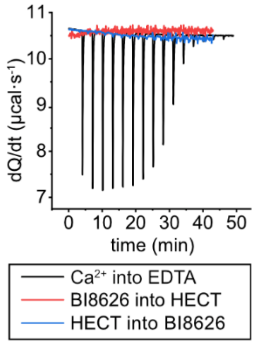

g

BI8626 (µM)	2.5	5	10	20	40	buffer	DMSO
T_m (HECT)	54.9	54.9	54.8	54.9	54.8	54.6	54.9
SD	0.2	0.2	0.2	0.1	0.2	0.1	0.2

i

BI8622 (µM)	2.5	5	10	20	40	buffer	DMSO
T_m (HECT)	54.9	54.9	54.8	54.9	54.7	54.6	54.9
SD	0.1	0.2	0.2	0.1	0.2	0.1	0.2

Supplementary Figure 2. Biophysical analyses of HUWE1^{HECT}-inhibitor interactions
(a) DSF analysis of the effect of the indicated concentrations of BI8626 on the stability of a vinylthioether-linked HUWE1^{HECT}~Ub proxy (10 µM). **(b)** Protein melting temperatures (T_m), derived from (a), represented as mean ± SD (n = 3 biological replicates). **(c, d)** Analogous analyses to (a, b) with BI8622. **(e)** ITC analysis of the interaction of BI8626 with the HUWE1^{HECT}~Ub proxy. No binding was detected. The interaction between Ca^{2+} and EDTA is shown as an instrument control. **(f)** DSF analysis of the effect of the indicated concentrations of BI8626 on the stability of HUWE1^{HECT} (10 µM). **(g)** Protein melting temperatures, derived from (f), represented as mean ± SD (n = 3 biological replicates). **(h, i)** Analogous analyses to (f, g) with BI8622. **(j)** ITC analysis of the interaction of BI8626 with HUWE1^{HECT}. No binding was detected. The interaction between Ca^{2+} and EDTA is shown as an instrument control. Source data are provided as a Source Data file.

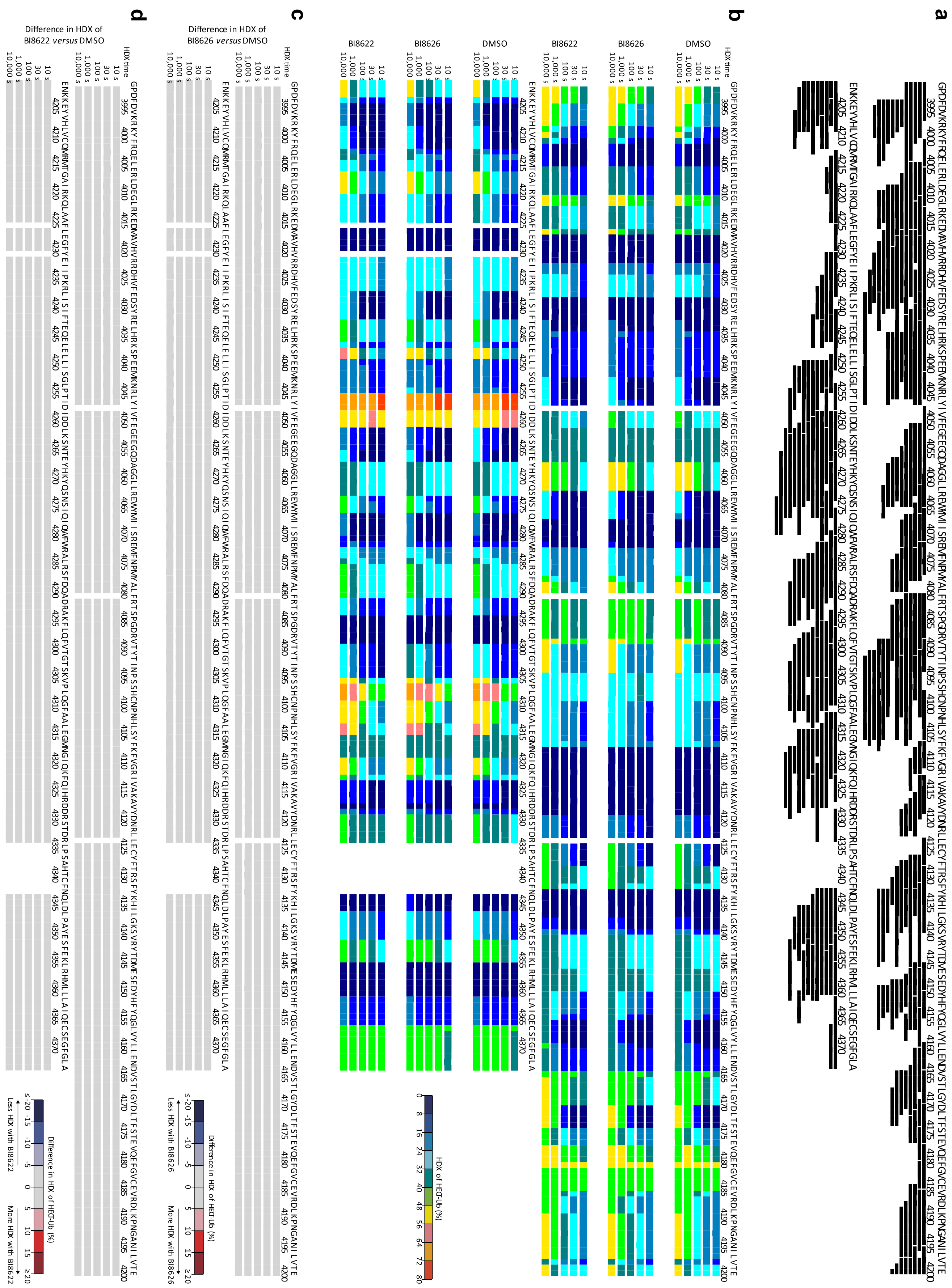

Supplementary Figure 3. HDX-MS analyses of inhibitor interactions with a HUWE1^{HECT}~Ub proxy

(a) Amino acid sequence of HUWE1^{HECT}, along with bars denoting proteolytic peptides identified in HDX-MS in all states and analyzed for deuterium incorporation. **(b)** Residue-specific HDX of the HUWE1^{HECT}~Ub proxy in the presence of a 10-fold molar excess of BI8626 and BI8622, respectively, or an equivalent volume of DMSO, at the indicated time points, shown along the HUWE1 amino acid sequence. **(c, d)** Comparative HDX-MS analysis of HUWE1^{HECT}~Ub ± BI8626 and BI8622, respectively, versus a DMSO control, plotted over the HUWE1 amino acid number. Note that the Ub-derived peptides are not displayed. For raw data, see Supplementary Data 1.

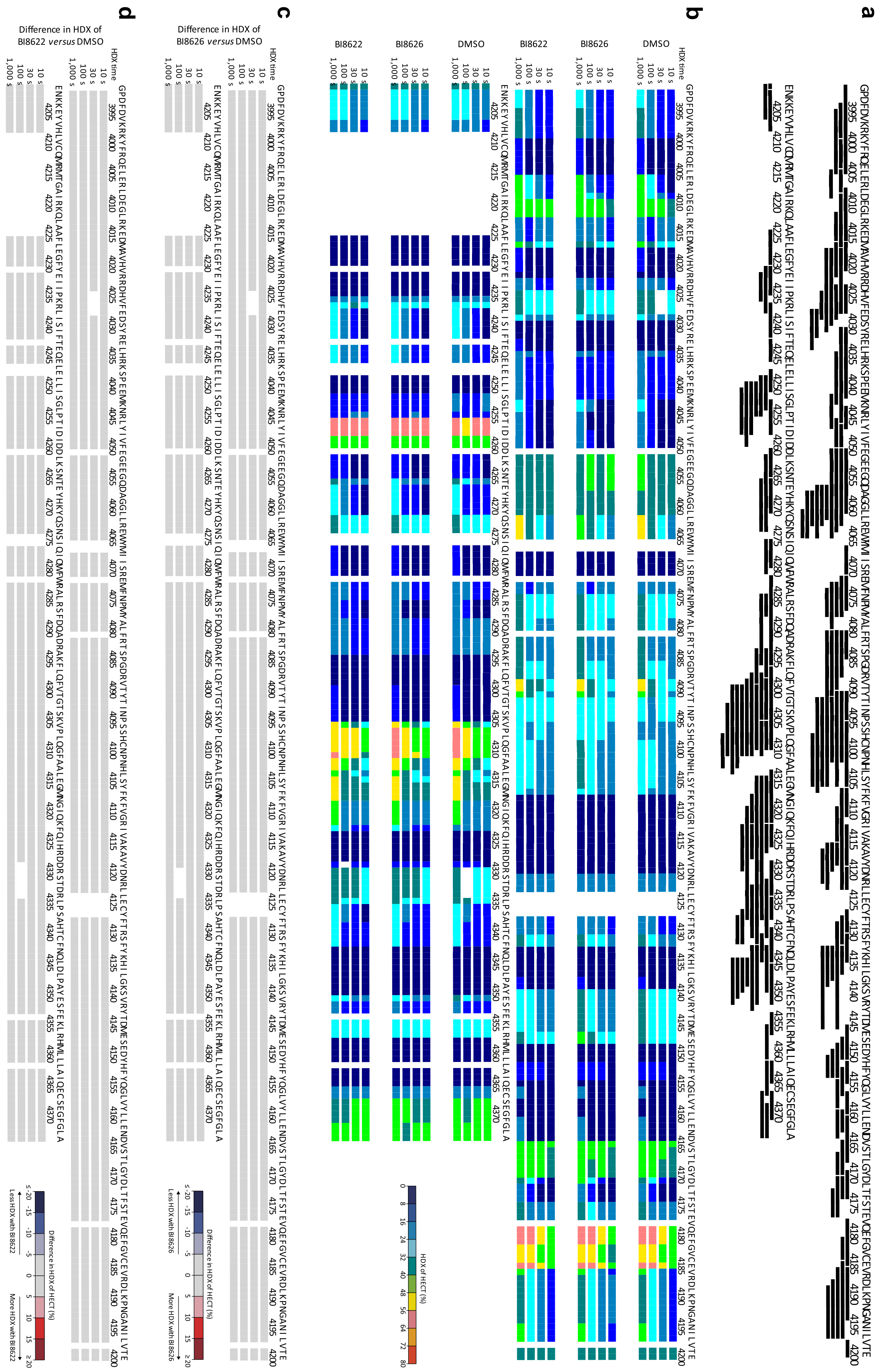

Supplementary Figure 4. HDX-MS analyses of inhibitor interactions with HUWE1^{HECT}

(a) Amino acid sequence of HUWE1^{HECT}, along with bars denoting proteolytic peptides identified by HDX-MS in all states and analyzed for deuterium incorporation. **(b)** Residue-specific HDX of HUWE1^{HECT} in the presence of a 10-fold molar excess of BI8626 and BI8622, respectively, or an equivalent volume of DMSO, at the indicated time points, shown along the amino acid sequence. **(c, d)** Comparative HDX-MS analysis of HUWE1^{HECT} in the presence of BI8626 and BI8622, respectively, versus a DMSO control, plotted over the HUWE1 amino acid number. No differences are observed. For raw data, see Supplementary Data 1.

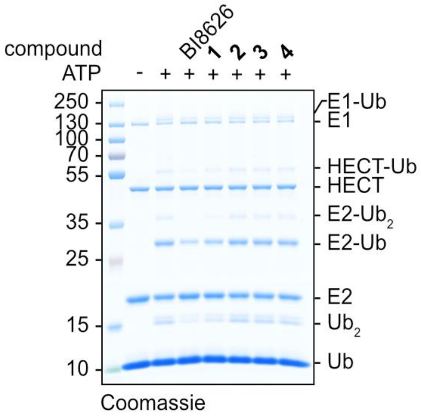

Supplementary Figure 5. Analysis of HUWE1^{HECT} inhibition by BI8626 derivatives
Coomassie-stained gel from Fig. 1d. Source data are provided as a Source Data file.

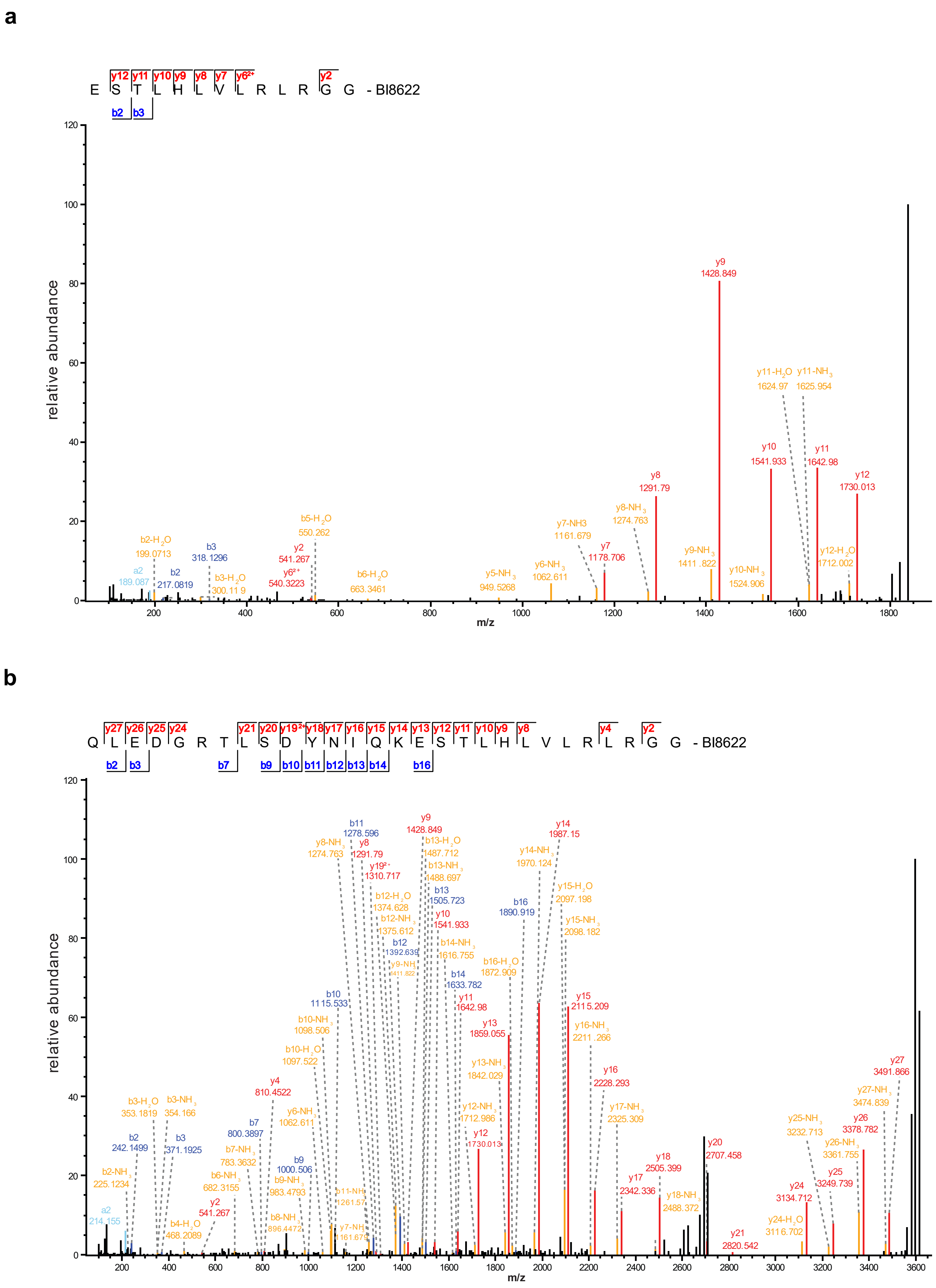

Supplementary Figure 6. MS/MS analyses of HUWE1^{HECT}-driven BI8622 ubiquitination

(a) Representative MS^2 spectrum of a BI8622-modified C-terminal Ub peptide, generated by LysC digestion (no missed cleavage) of HUWE1^{HECT}-driven *in vitro* ubiquitination reactions in the presence of BI8622. **(b)** Analogous MS^2 spectrum to (a) for a LysC-generated Ub peptide with one missed cleavage. In both panels, the observed fragment ions (b- and y-series) are annotated, confirming sequence coverage and site-specific BI8622 modification of the Ub C-terminus.

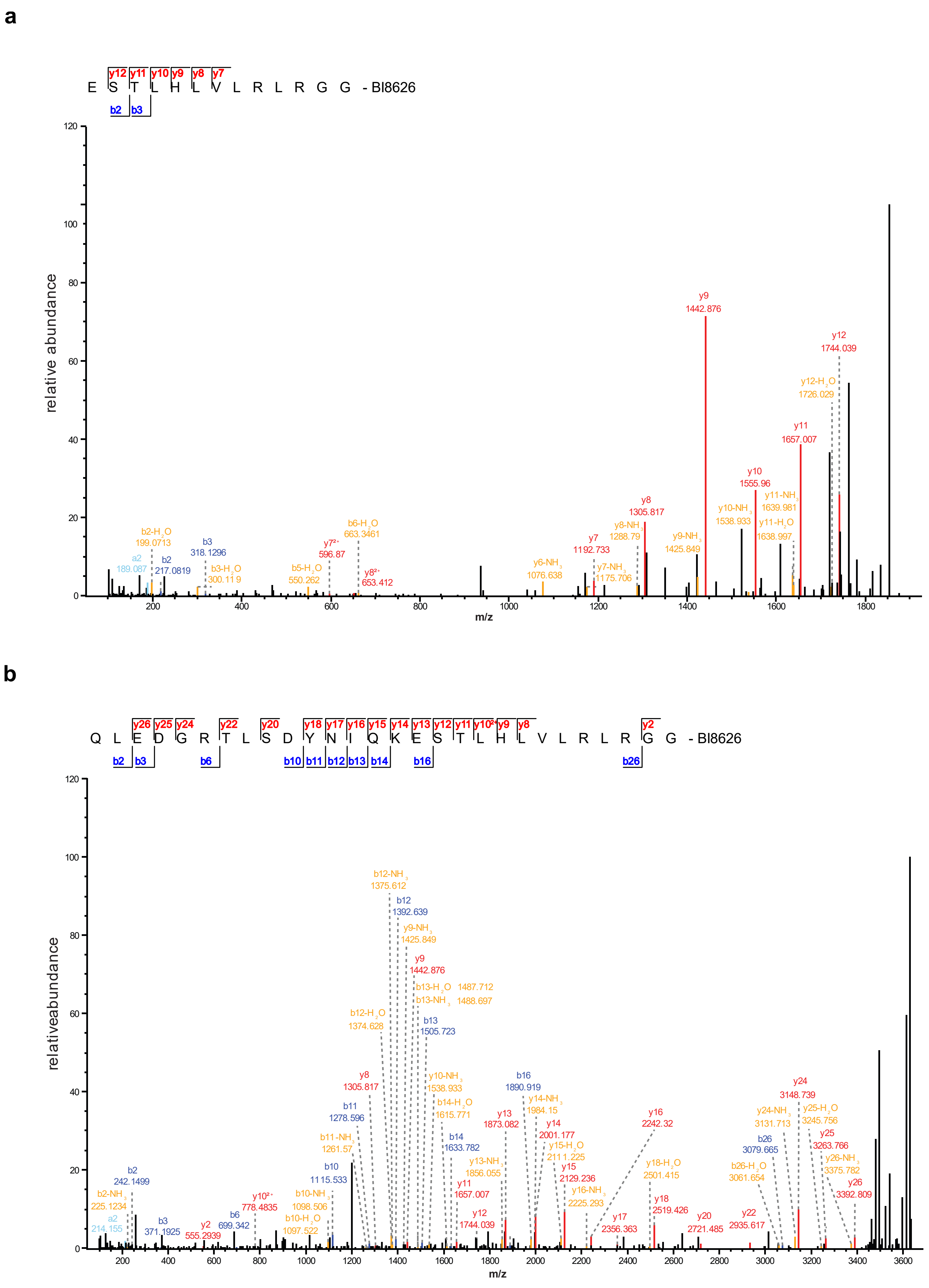

Supplementary Figure 7. MS/MS analyses of HUWE1^HECT^-driven BI8626 ubiquitination

(a) Representative MS^2 spectrum of a BI8626-modified C-terminal Ub peptide, generated by LysC digestion (no missed cleavage) of HUWE1^HECT^-driven *in vitro* ubiquitination reactions in the presence of BI8626. **(b)** Analogous MS^2 spectrum to (a) for a LysC-generated Ub peptide with one missed cleavage. In both panels, the observed fragment ions (b- and y-series) are annotated, confirming sequence coverage and site-specific BI8626 modification of the Ub C-terminus.

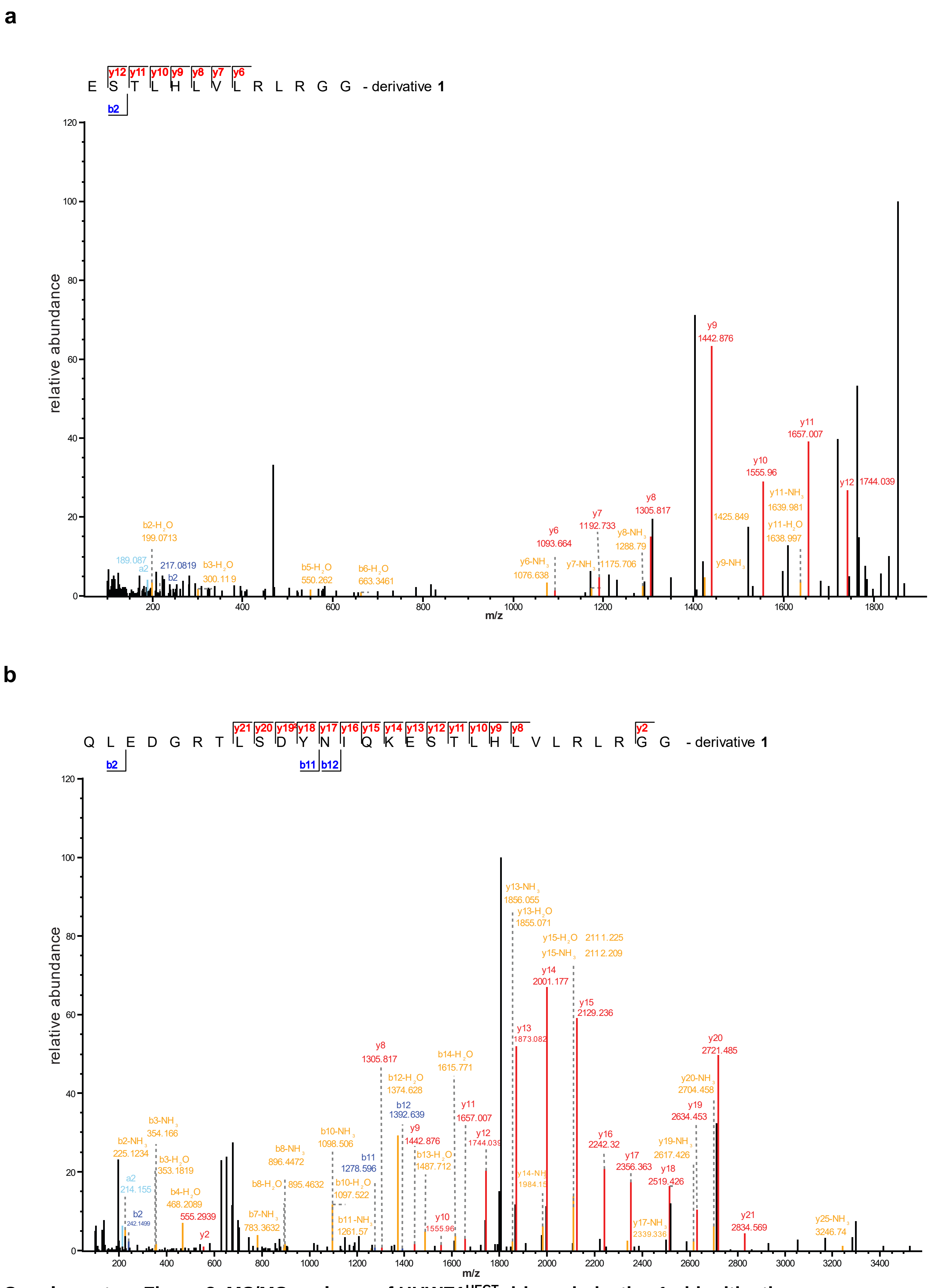

Supplementary Figure 8. MS/MS analyses of HUWE1^{HECT}-driven derivative 1 ubiquitination
(a) Representative MS^2 spectrum of a derivative **1** -modified C-terminal Ub peptide, generated by LysC digestion (no missed cleavage) of HUWE1^{HECT}-driven *in vitro* ubiquitination reactions in the presence of derivative **1**. **(b)** Analogous MS^2 spectrum to (a) for a LysC-generated Ub peptide with one missed cleavage. In both panels, the observed fragment ions (b- and y-series) are annotated, confirming sequence coverage and site-specific derivative **1** modification of the Ub C-terminus.

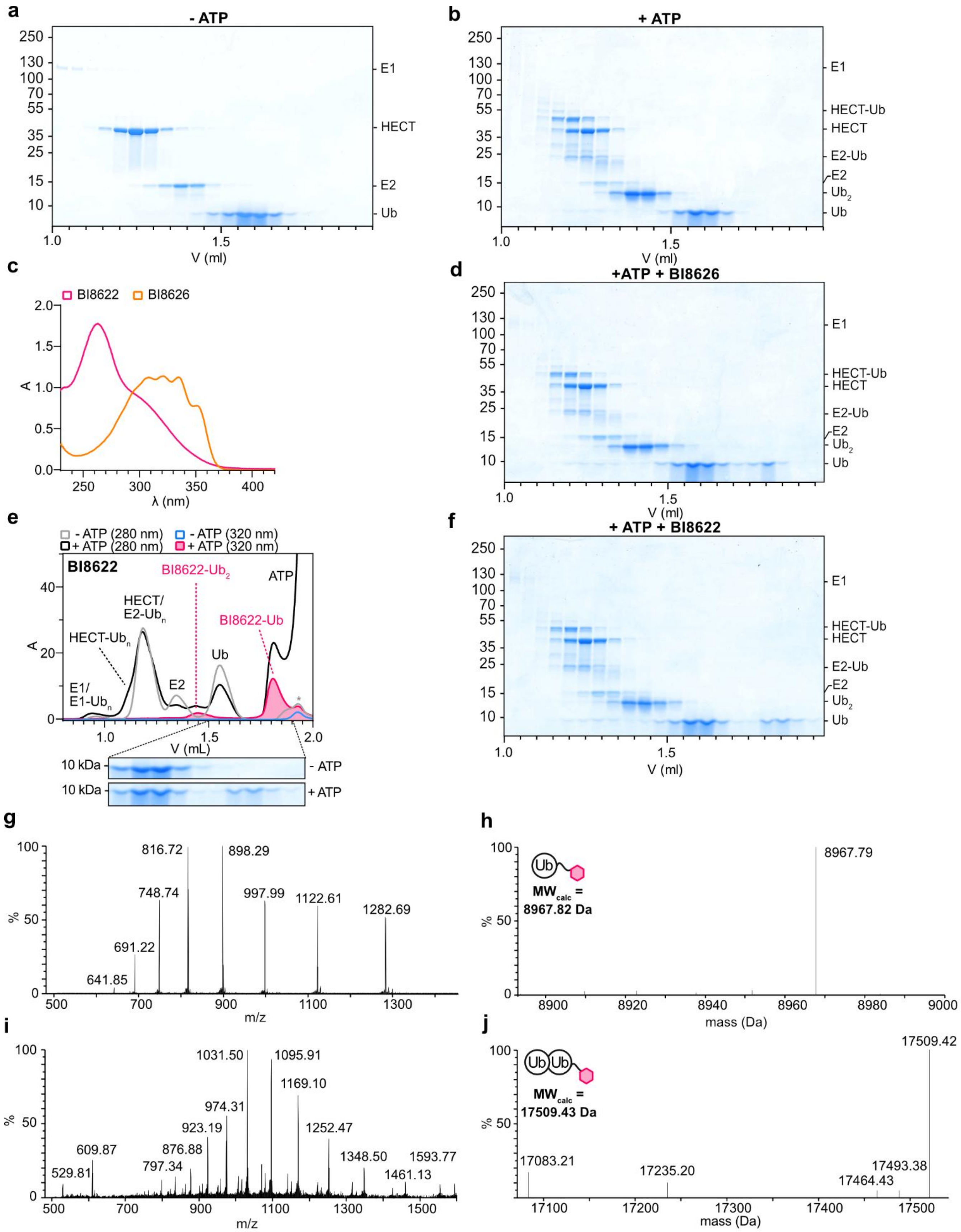

Supplementary Figure 9. Biochemical and intact MS analyses of BI8622 and BI8626 ubiquitination

(a) Full SDS-PAGE analysis of the SEC elution fractions from Fig. 2a, for the ATP-free mixture. **(b)** Full SDS-PAGE analysis of the SEC elution fractions from Fig. 2a, for the ATP-containing reaction. **(c)** Superposition of the UV-absorbance spectra of BI8622 and BI8626 in water **(d)** Full SDS-PAGE analysis of the SEC elution fractions from Fig. 2b, for the BI8626-containing reaction. **(e)** SEC-based fractionation of HUWE1^{HECT}-driven ubiquitination reactions in the presence of 20 μM BI8622 (top). SDS-PAGE analyses for the indicated elution fractions, focusing on Ub-containing fractions (bottom). Note that additional, delayed Ub-containing fractions are observed compared to ATP-free or inhibitor-free (Fig. 2a) conditions. **(f)** Full SDS-PAGE analysis of the SEC elution fractions from (e), for the BI8622-containing reaction (full gel). **(g, h)** Intact protein MS (g) and deconvoluted neutral MW spectrum (h) of the major BI8622-containing elution peak (pink; V ~1.85 mL) from (e). The experimentally determined and calculated molecular weights (MWs) are specified. **(i, j)** Intact protein MS (i) and deconvoluted neutral MW spectrum (j) of the minor BI8622-containing elution peak (pink; V ~1.44 mL) from (e). Source data are provided as a Source Data file.

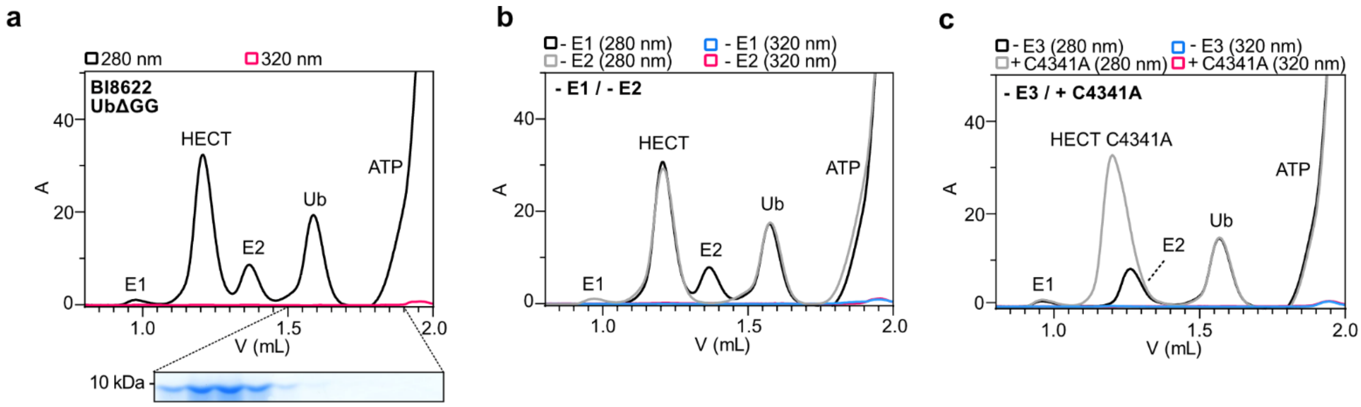

Supplementary Figure 10. Mechanistic analyses of BI8622 ubiquitination
(a) SEC-based fractionation (top) and SDS-PAGE analysis (bottom) of a HUWE1^{HECT}-driven ubiquitination reaction in the presence of 20 μM BI8622, analogous to Supplementary Fig. 9e, but employing UbΔGG instead of WT Ub. **(b)** SEC-based fractionation of HUWE1^{HECT}-driven ubiquitination reactions in the presence of 20 μM BI8622, analogous to Supplementary Fig. 9e, but devoid of E1 and E2, respectively. **(c)** SEC-based fractionation of ubiquitination reactions in the presence of 20 μM BI8622, analogous to Supplementary Fig. 9e, but devoid of HUWE1^{HECT} or employing a catalytically inactive HUWE1^{HECT} variant (C4341A) instead of the WT. Source data are provided as a Source Data file.

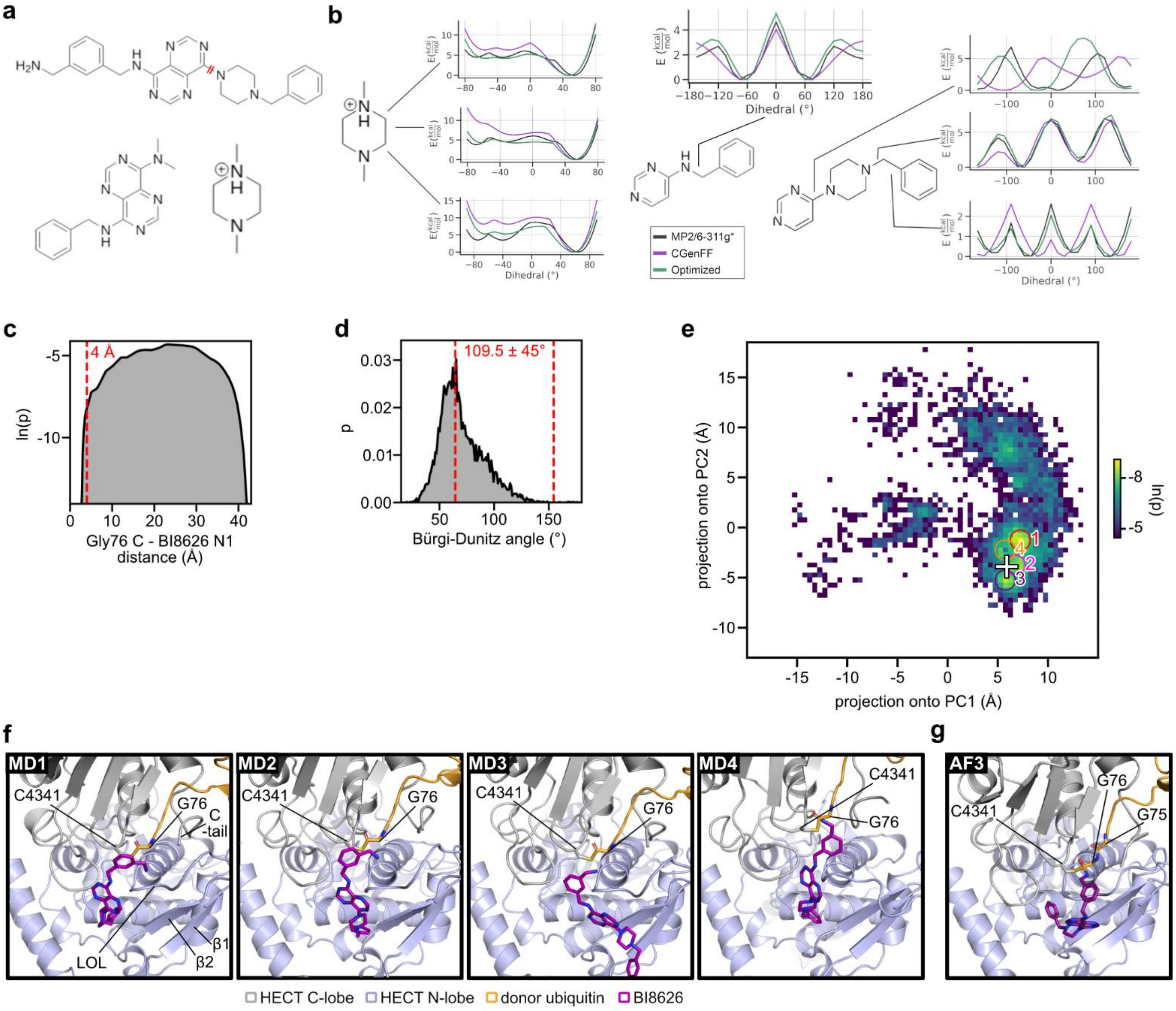

Supplementary Figure 11. Structural predictions of BI8626 attacking a HUWE1^{HECT}-Ub complex

(a) Fragmentation scheme used to split BI8626 into smaller fragments for partial charge optimization. **(b)** Comparison of the dihedral potential calculated with: MP2/6-311g* (black line), initial parameters from the charm general force field (purple line) and the optimized force field (green line). **(c)** Distribution *p* of distances between the C atom of Gly76 of the HUWE1^{HECT}-linked donor Ub and the primary amino nitrogen atom of BI8626 sampled during 100 2-μs MD simulations. The red line indicates the chosen threshold distance. **(d)** Distribution of Bürgi-Dunitz angles for all conformations with a distance < 4 Å, as defined in (c). The red lines indicate the chosen threshold angles (accounting for rotations of the amino group of BI8626). **(e)** Trajectories projected onto the two dominant conformational modes (principal components 1 and 2) of BI8626 relative to the active site. The frequency of observing a conformation is indicated by colors, and maxima in this landscape are indicated by circles. For comparison, a top-scoring AF3 model (predicted when restraining the primary amino nitrogen atom of BI8626 at Gly76 of the donor Ub, for details, see Supplementary Methods) was projected onto PC1 and PC2, indicated by a white cross. **(f)** Detail views of BI8626 in the conformations corresponding to the 4 probability maxima from (e), all shown in the HECT domain orientation. The active-site cysteine of HUWE1 and the C-terminal residues of the donor Ub are labeled in all poses. In pose 1, we additionally labeled critical elements for aminolysis, including the C-terminal extension ('C-tail') of the HECT C-lobe, the LOL (ligase-organizing loop) and the β1/ β2-sheet of the HECT N-lobe **(g)** Detail view of BI8626 in a top-scoring AF3 prediction (white cross in (e)), shown in the same HECT domain orientation as (f).

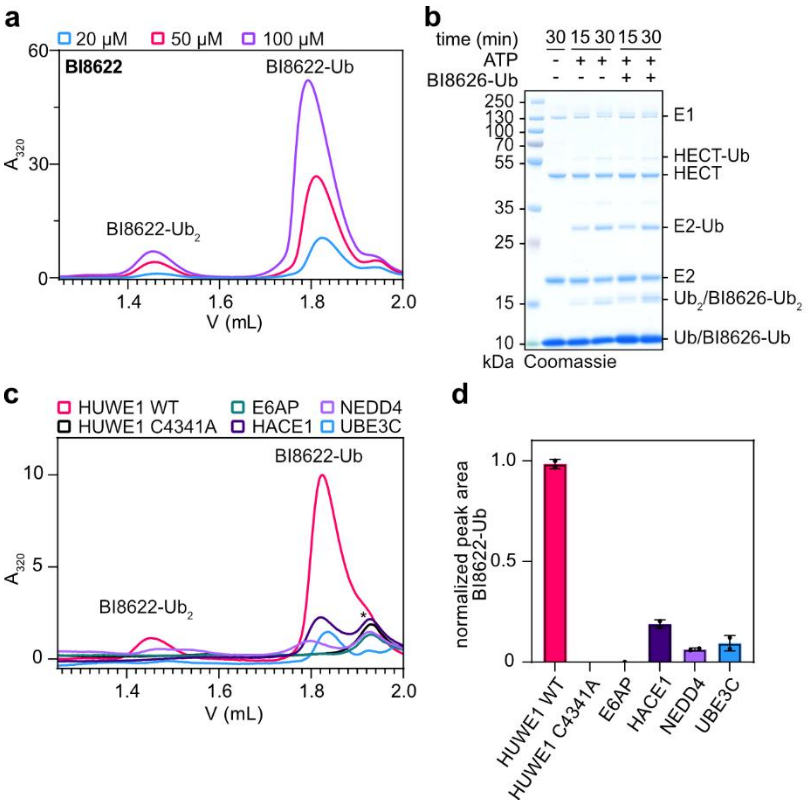

Supplementary Figure 12. BI8622 provides a selective substrate for HUWE1^{HECT}.
(a) SEC-based analyses of HUWE1^{HECT}-driven ubiquitination reactions at different BI8622 concentrations, focusing on the mono- and diubiquitinated BI8622 products, monitored by absorbance at 320 nm. **(b)** Coomassie-stained version of the gel from Fig. 3b. **(c)** SEC-based analyses of BI8622-ubiquitination, driven by the indicated HECT domains, focusing on the mono- and diubiquitinated BI8622 products, monitored by absorbance at 320 nm. **(d)** Quantification of BI8622 monoubiquitination by the indicated HECT domains across 2 SEC analyses as shown in (c). The signal produced by HUWE1^{HECT} was normalized to 1. Data are represented as mean ± SD (n = 2 biological replicates). Source data are provided as a Source Data file.

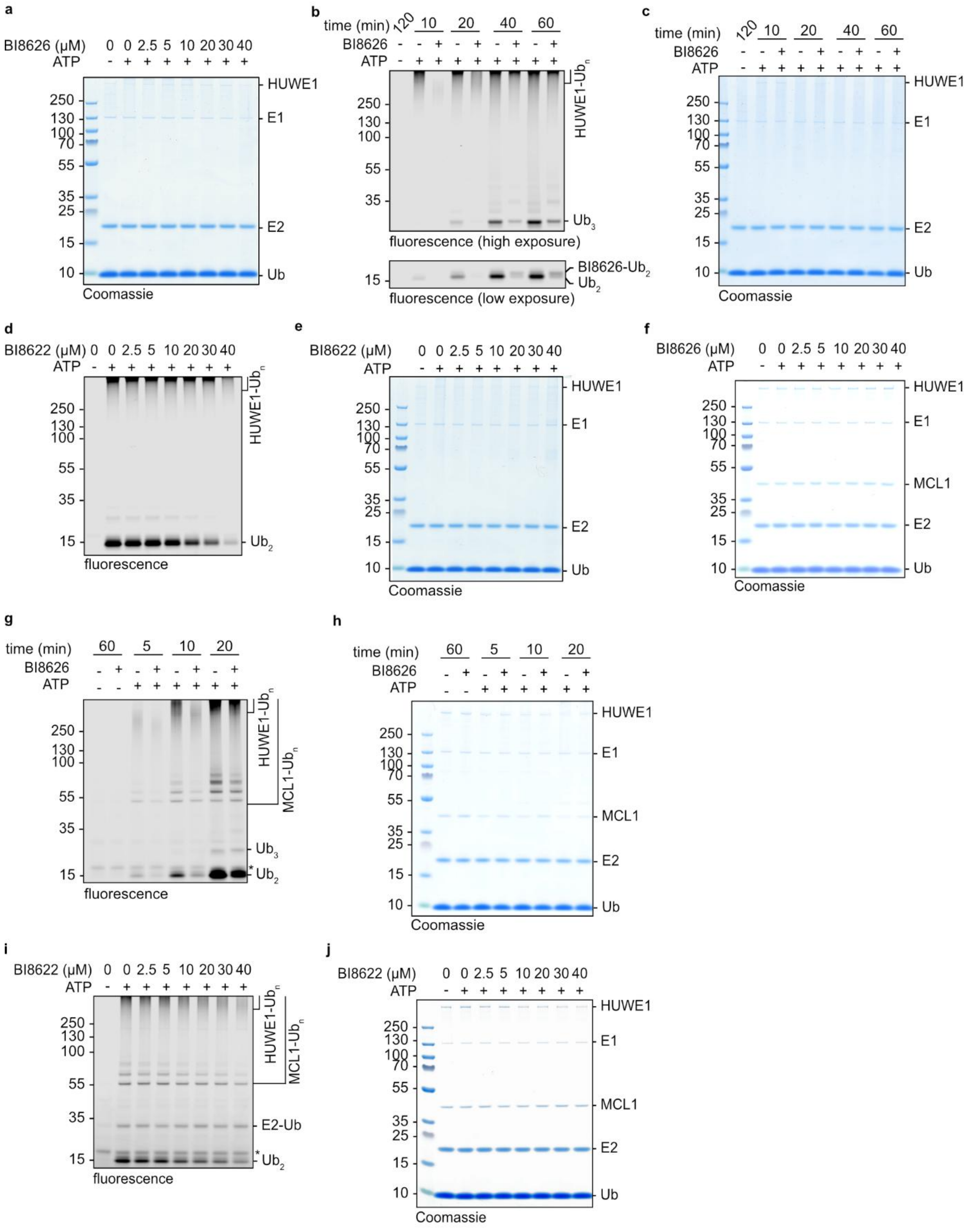

Supplementary Figure 13. Mechanistic analyses of compound-mediated $HUWE1^{FL}$ inhibition

(a) Coomassie-stained full gel for the sections from Fig. 4a. **(b)** Multi-turnover $HUWE1^{FL}$-driven autoubiquitination reactions in the presence of 20 μM BI8626, without protein substrate, monitored over time via a fluorescent Ub tracer. **(c)** Protein input from (b), shown by Coomassie staining. **(d)** Multi-turnover $HUWE1^{FL}$-driven ubiquitination reactions in the presence of the indicated BI8622 concentrations, without protein substrate, monitored via a fluorescent Ub tracer. **(e)** Protein input from (d), shown by Coomassie staining. **(f)** Coomassie-stained version of the gel from Fig. 4b. **(g)** Multi-turnover $HUWE1^{FL}$-driven MCL1 ubiquitination reactions in the presence of 20 μM BI8626, monitored over time via a fluorescent Ub tracer. **(h)** Protein input from (g), shown by Coomassie staining. **(i)** Multi-turnover $HUWE1^{FL}$-driven MCL1 ubiquitination reactions in the presence of the indicated concentrations of BI8622, monitored via a fluorescent Ub tracer. **(j)** Protein input from (i), shown by Coomassie staining. Source data are provided as a Source Data file.

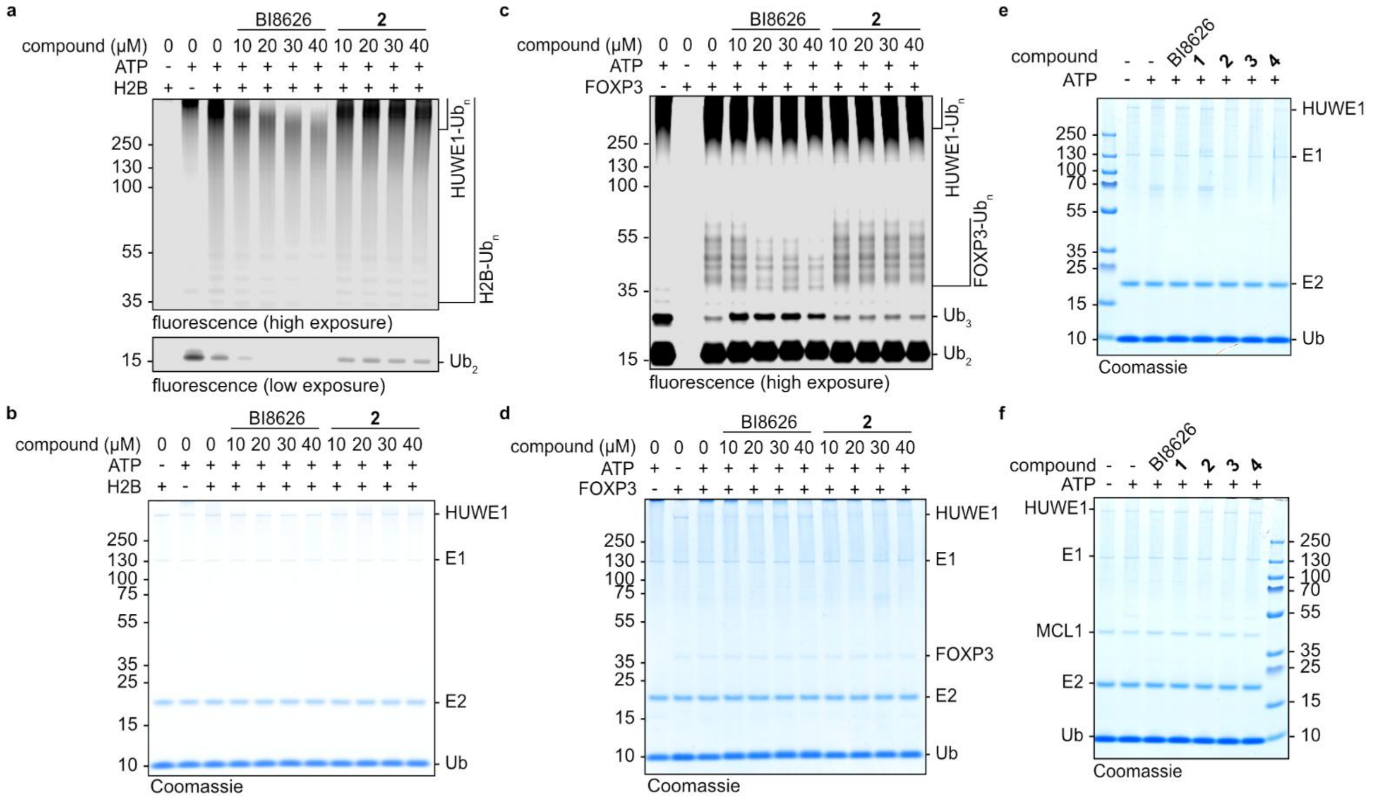

Supplementary Figure 14. Analyses of HUWE1^{FL}-driven substrate ubiquitination

(A) Multi-turnover HUWE1^{FL}-driven H2B ubiquitination reactions in the presence of the indicated BI8626 and compound **2** concentrations, respectively, followed by a fluorescent Ub tracer. **(b)** Protein input from (a), shown by Coomassie staining. **(c)** Multi-turnover HUWE1^{FL}-driven FOXP3 ubiquitination reactions in the presence of the indicated BI8626 and compound **2** concentrations, respectively, followed by a fluorescent Ub tracer. **(d)** Protein input from (c), shown by Coomassie staining. **(e)** Coomassie-stained version of the gel from Fig. 4c. **(f)** Coomassie-stained version of the gel from Fig. 4d. Source data are provided as a Source Data file.

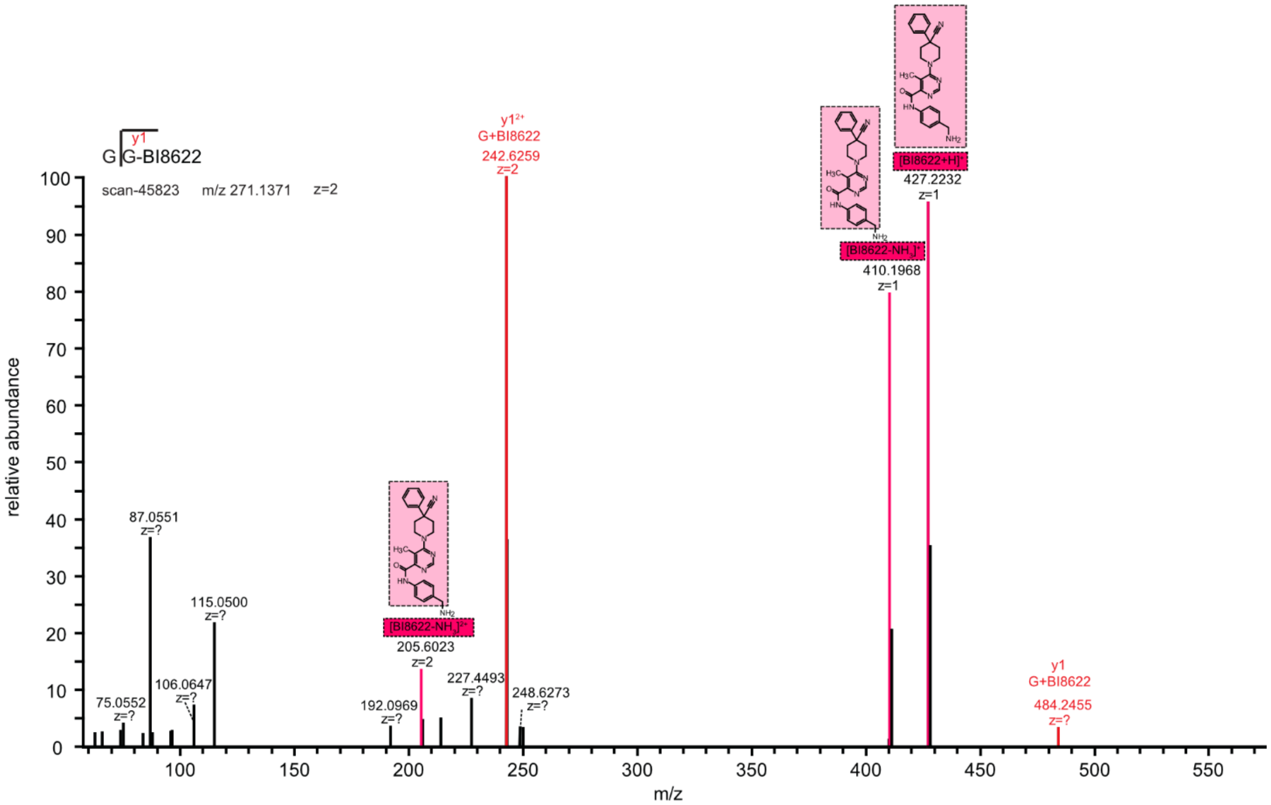

Supplementary Figure 15. Targeted MS/MS analyses of HUWE1^{FL}-driven BI8622 ubiquitination

Representative targeted MS² spectrum of a BI8622-modified C-terminal Ub peptide (precursor m/z 271.1371, z = 2), generated by tryptic digestion of HUWE1^{FL}-driven *in vitro* ubiquitination reactions and analyzed by targeted LC-MS/MS. Annotated fragment ions at m/z 242.6259 (z = 2) and 484.2455 (z = 1) correspond to the glycine–BI8622 (G–BI8622) conjugate. Additional fragment ions at m/z 205.6023 (z = 2), 410.1968 (z = 1), and 427.2232 (z = 1) are characteristic of BI8622. Structural representations of key fragmentation events are indicated, with boxed parts corresponding to the respective fragment ions.

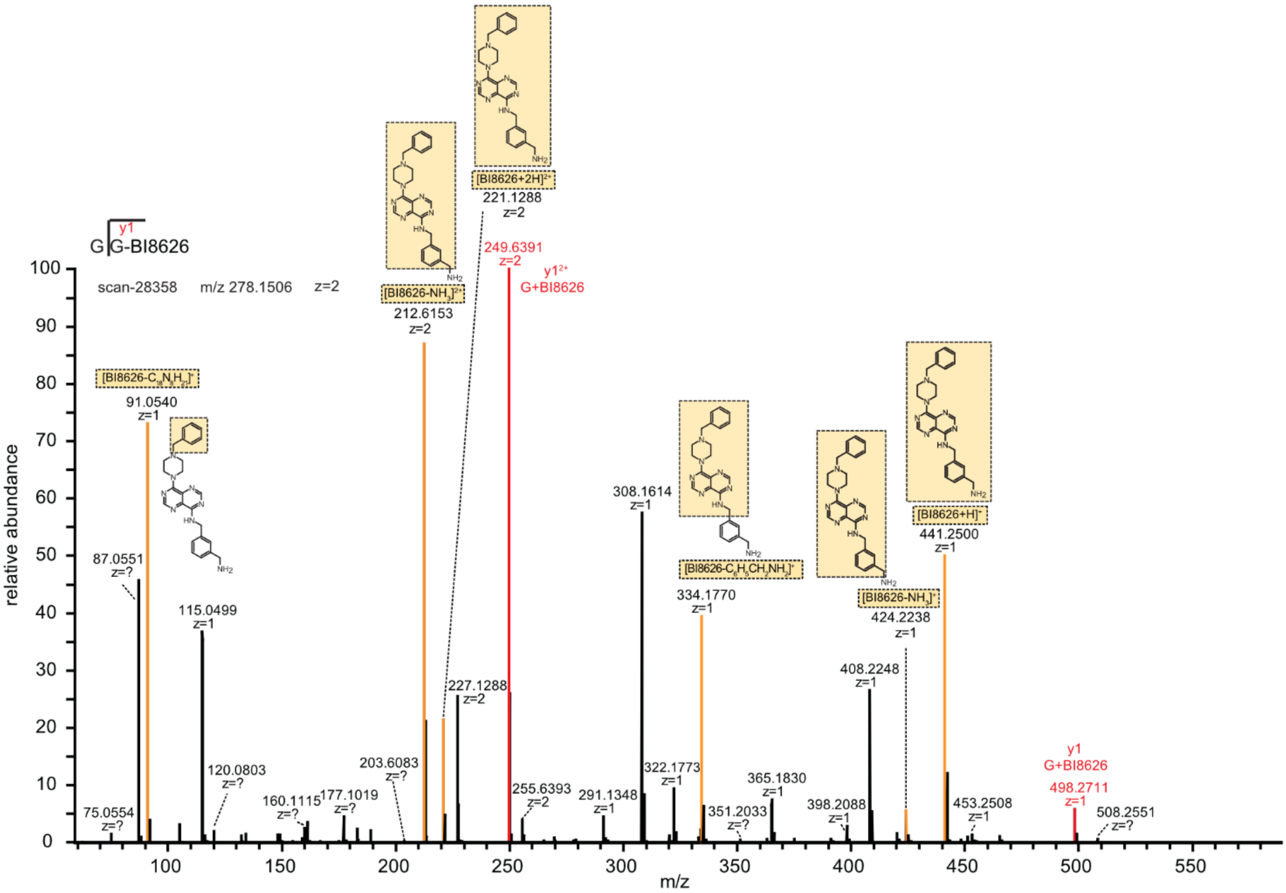

Supplementary Figure 16. Targeted MS/MS analyses of HUWE1^{FL}-driven BI8626 ubiquitination
Representative targeted MS² spectrum of a BI8626-modified C-terminal Ub peptide (precursor m/z 278.1506, z = 2), generated by tryptic digestion of HUWE1^{FL}-driven *in vitro* ubiquitination reactions and analyzed by targeted LC-MS/MS. Annotated fragment ions at m/z 249.6391 (z = 2) and 498.2711 (z = 1) correspond to the glycine–BI8626 conjugate. Additional fragment ions derived from BI8626 itself are observed at m/z 91.0540 (z = 1), 212.6153 (z = 2), 221.1288 (z = 2), 334.1770 (z = 1), 424.2238 (z = 1), and 441.2500 (z = 1). Structural representations of key fragmentation events are indicated, with boxed parts corresponding to the respective fragment ions.

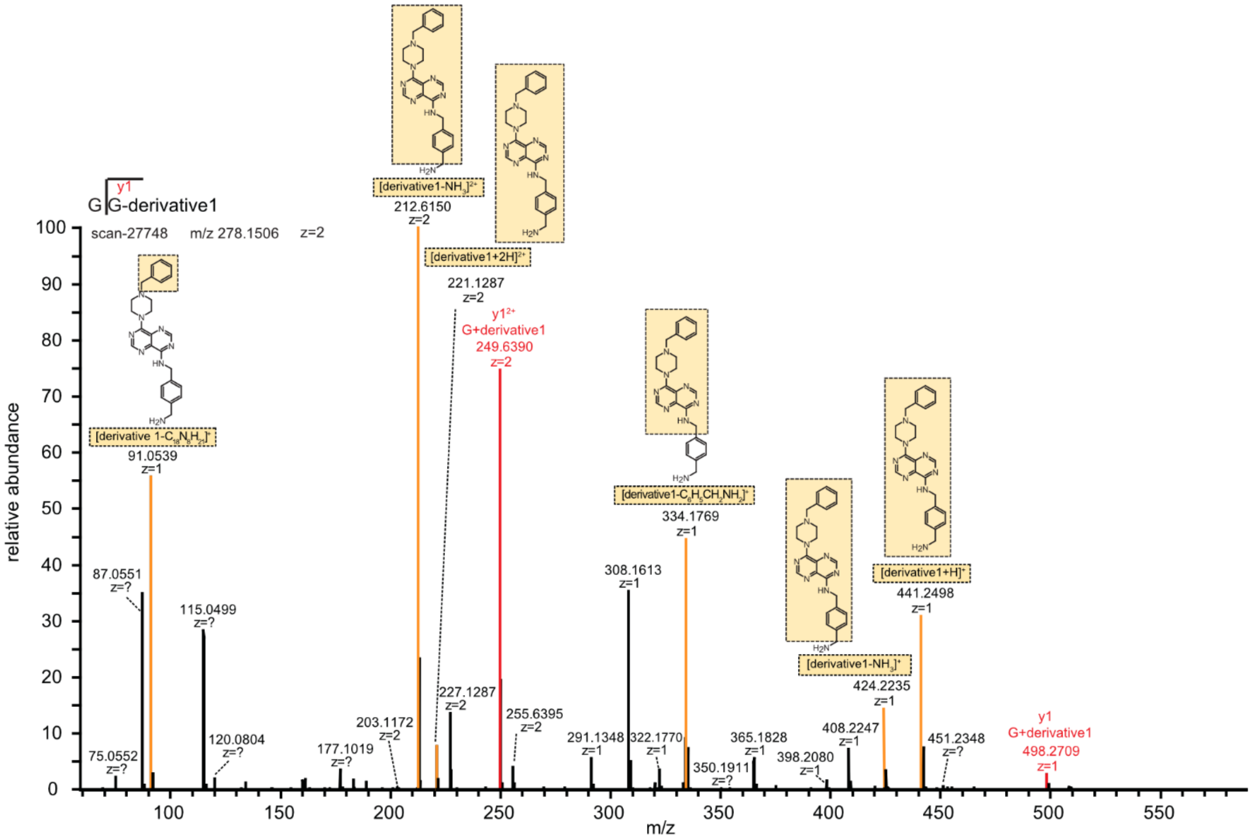

Supplementary Figure 17. Targeted MS/MS analyses of HUWE1^{FL}-driven derivative 1 ubiquitination
Representative targeted MS² spectrum of a derivative **1**–modified C-terminal Ub peptide (precursor m/z 278.1506, z = 2), generated by tryptic digestion of HUWE1^{FL}-driven *in vitro* ubiquitination reactions and analyzed by targeted LC-MS/MS. Annotated fragment ions at m/z 249.6390 (z = 2) and 498.2709 (z = 1) correspond to the glycine–derivative **1** (G–derivative **1**) conjugate. Additional fragment ions resulting from derivative **1** itself are observed at m/z 91.0539 (z = 1), 212.6150 (z = 2), 221.1287 (z = 2), 334.1769 (z = 1), 424.2235 (z = 1), and 441.2498 (z = 1), corresponding to characteristic cleavages of the compound. Structural representations of key fragmentation events are indicated, with boxed parts corresponding to the respective fragment ions.

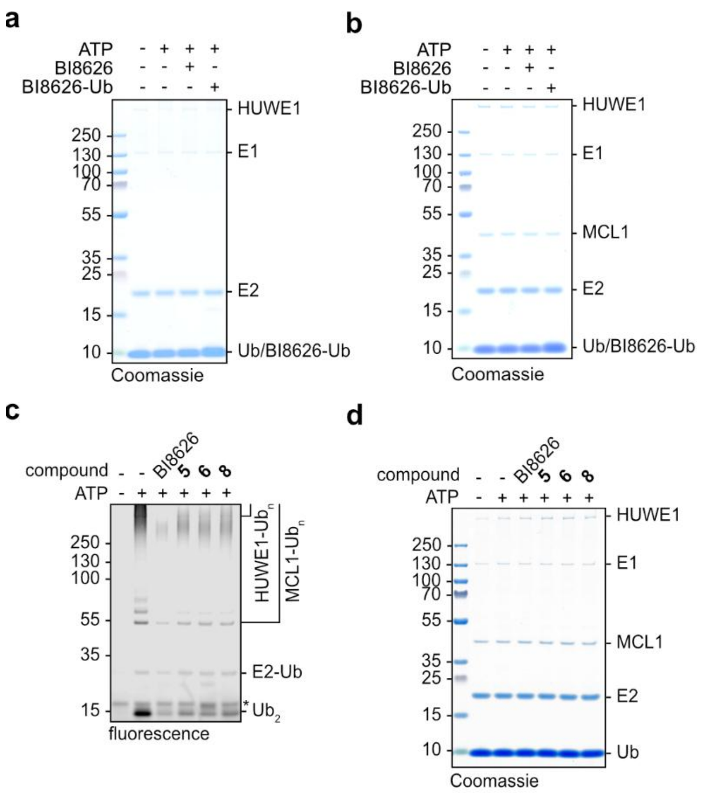

Supplementary Figure 18. Mechanistic analyses of HUWE1^{FL}-driven BI8626 ubiquitination
(a) Coomassie-stained full gel for the sections shown in Fig. 4e. **(b)** Coomassie-stained full gel for the sections shown in Fig. 4f. **(c)** Multi-turnover HUWE1^{FL}-driven MCL1 ubiquitination reactions, containing 20 μM of the indicated compounds (Table 1), monitored via a fluorescent Ub tracer. **(d)** Protein input from (c), shown by Coomassie staining. Source data are provided as a Source Data file.

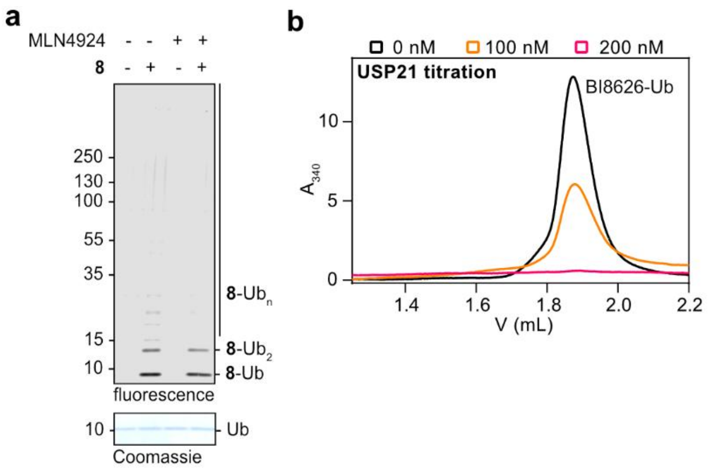

Supplementary Figure 19. Cell-based and biochemical analyses of compound ubiquitination
(a) Click-chemistry-based detection of cellular derivative **8** ubiquitination in the absence and presence of the NAE1 inhibitor MLN4924. Note that compound **8** is used here instead of **6**, for it provided enhanced signal (see Methods). **(b)** SEC analysis of purified BI8626-Ub upon incubation with the indicated USP21 concentrations. Source data are provided as Source Data file.

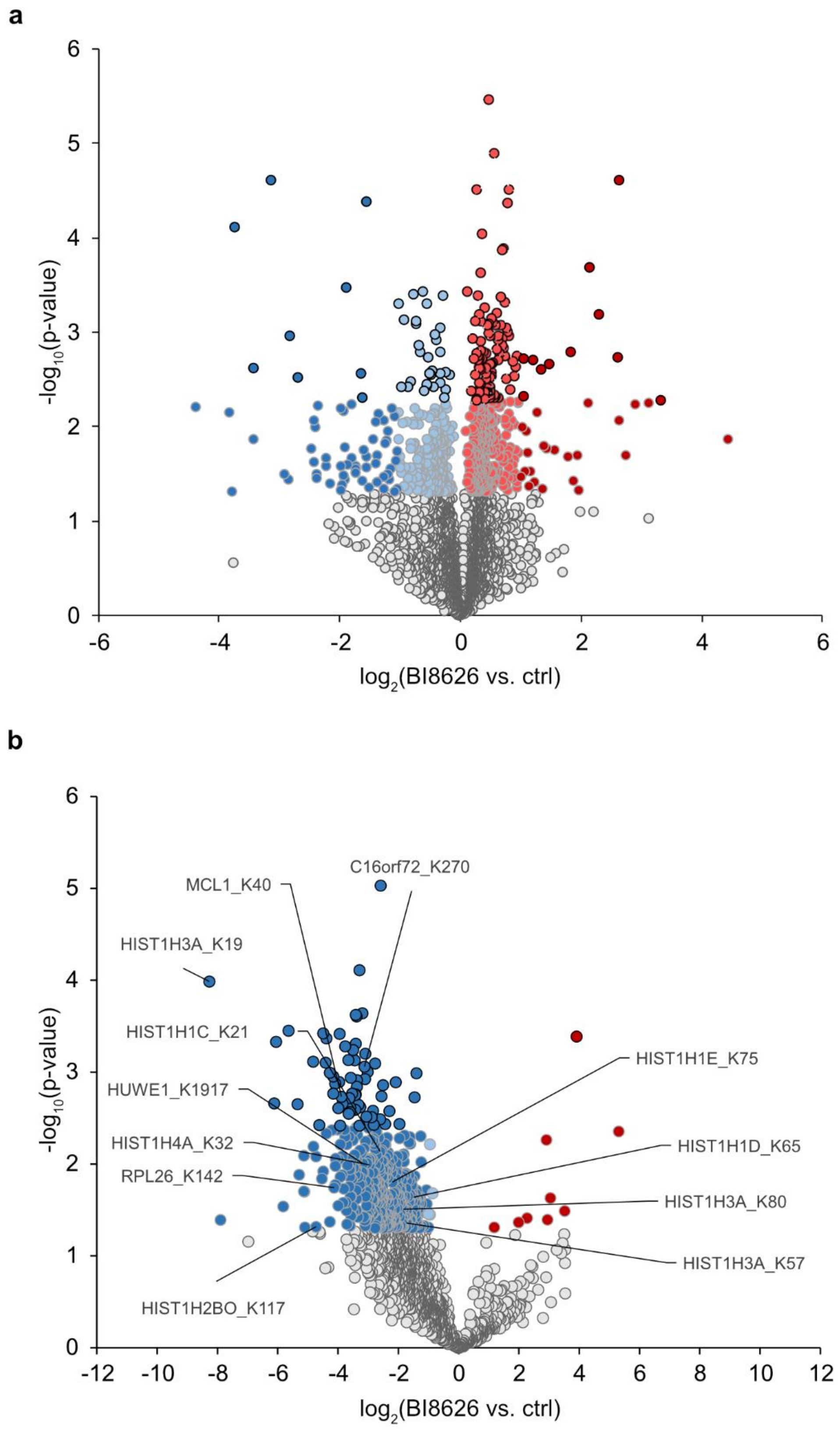

Supplementary Figure 20. Global quantitative and diGly proteomic analyses

(a) Volcano plot depicting changes in total protein abundance of HEK293T cells upon BI8626 treatment (15 μM for 24 h), compared to DMSO-treated controls. Experiments were performed in biological quadruplicates and statistical analysis using two-sided two sample Student's t-test (p-value<0.05) with permutations-based correction (FDR<0.05). Significantly altered proteins are labeled in dark ($log_2FC \geq |1|$) and light color ($log_2|0<FC<1|$), respectively. Black circles represent a significance of FDR<0.05, whereas grey circles reach threshold of p<0.05. **(b)** Volcano plot depicting changes in the ubiquitinome, analogous to (a). Experiments were performed in biological quadruplicates and statistical analysis using two-sided two sample Student's t-test (p-value<0.05) with permutations-based correction (FDR<0.05). Significantly altered proteins are labeled in dark ($log_2FC \geq |1|$) and light color ($log_2|0<FC<1|$), respectively. Black circles represent a significance of FDR<0.05, whereas grey circles reach threshold of p<0.05. Ubiquitination sites detected on HUWE1 and selected HUWE1 substrates are labeled.

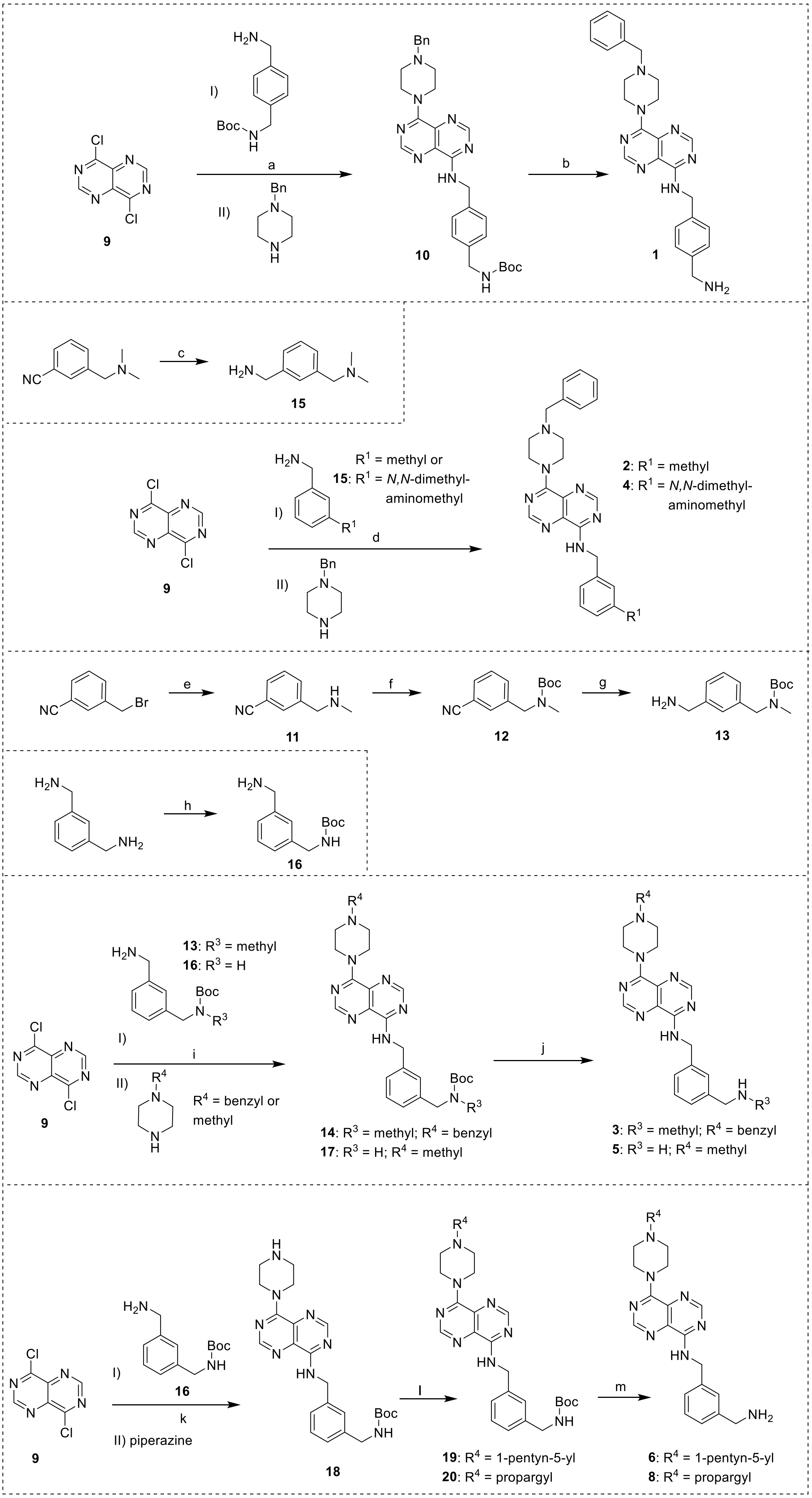

Supplementary Figure 21. Overview of compound syntheses

a

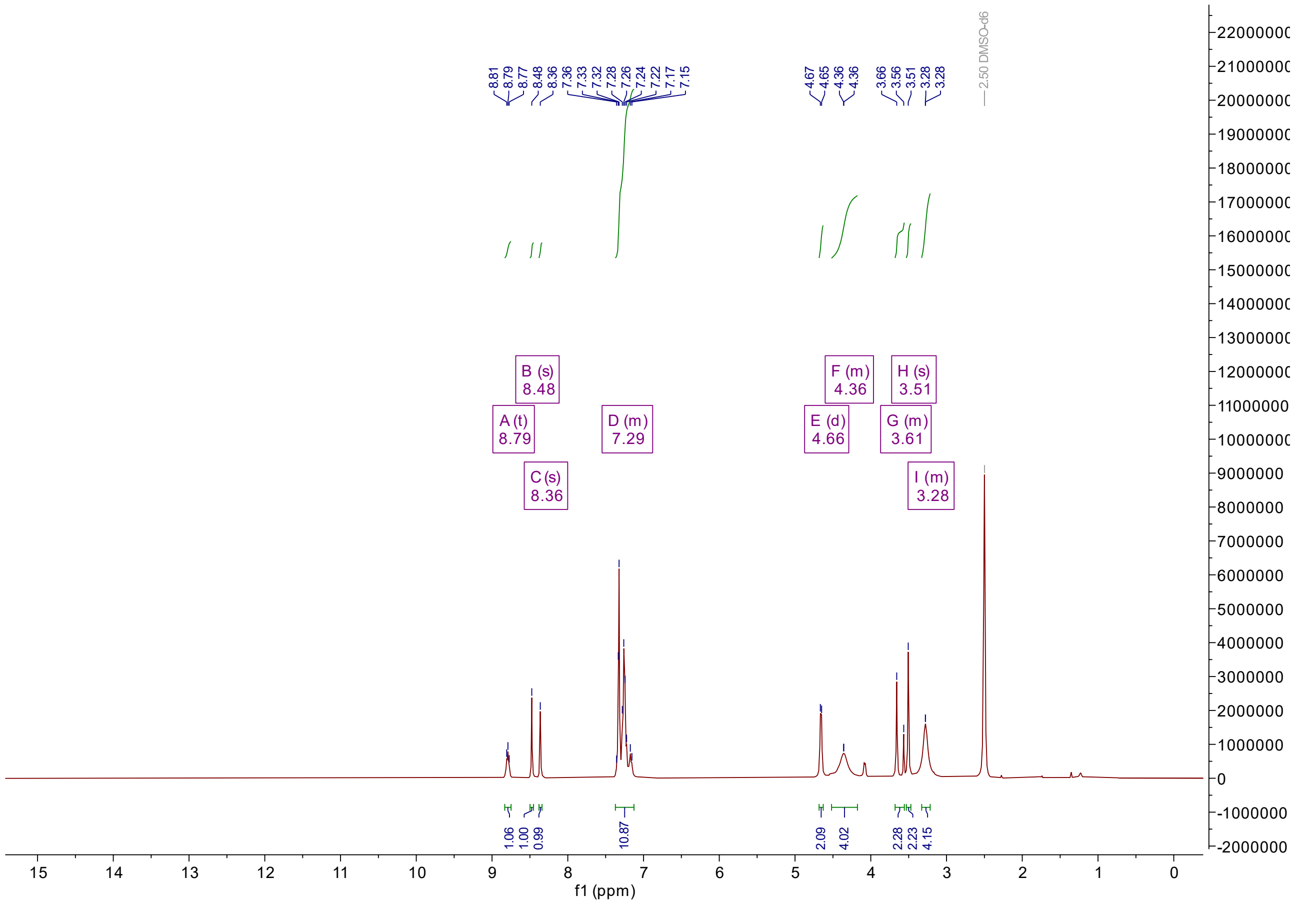

b

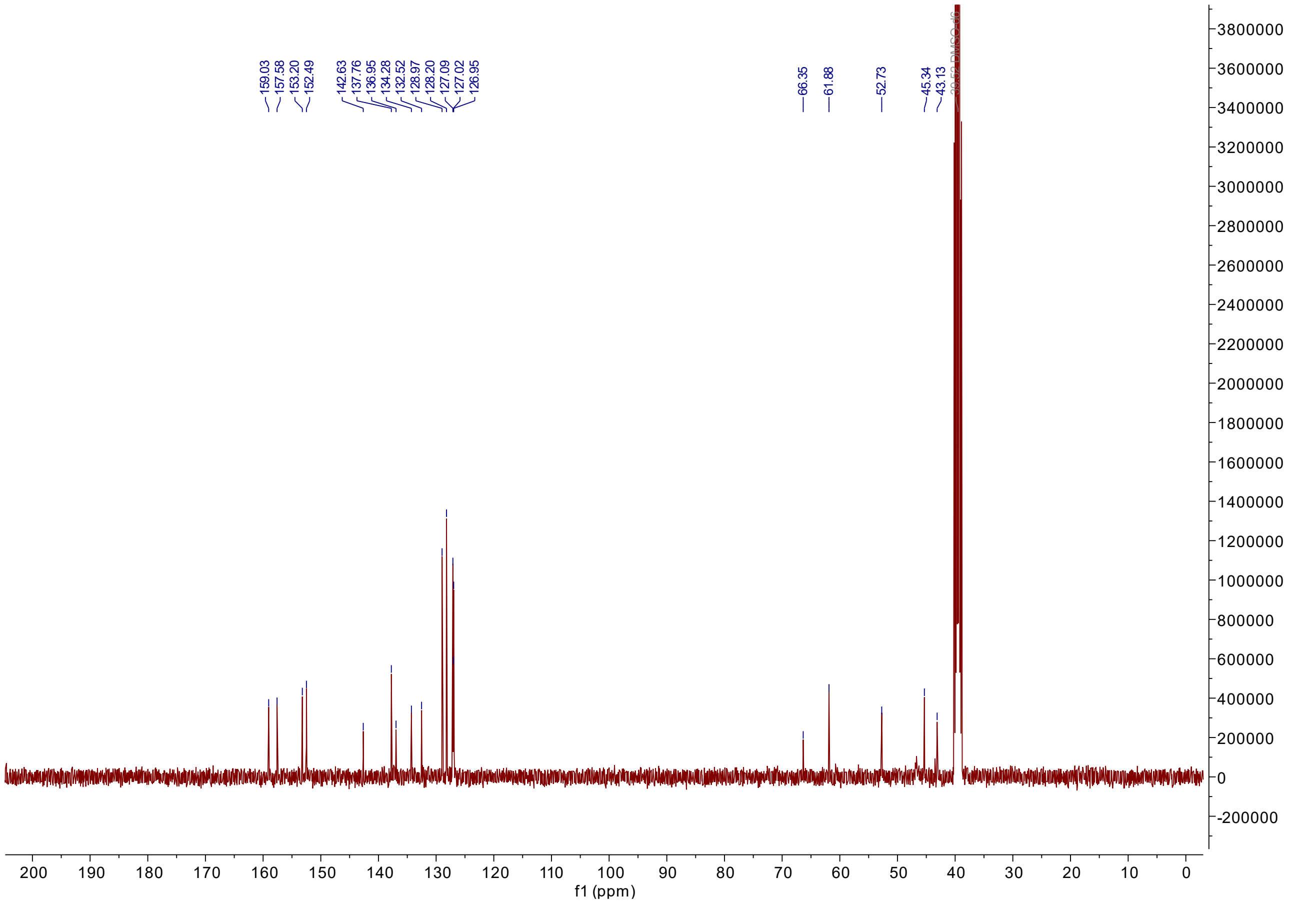

Supplementary Figure 22. NMR analyses of BI8626 derivative 1
(a) ^{1}H and **(b)** ^{13}C NMR spectrum of the synthesized derivative 1 (see Table 1).

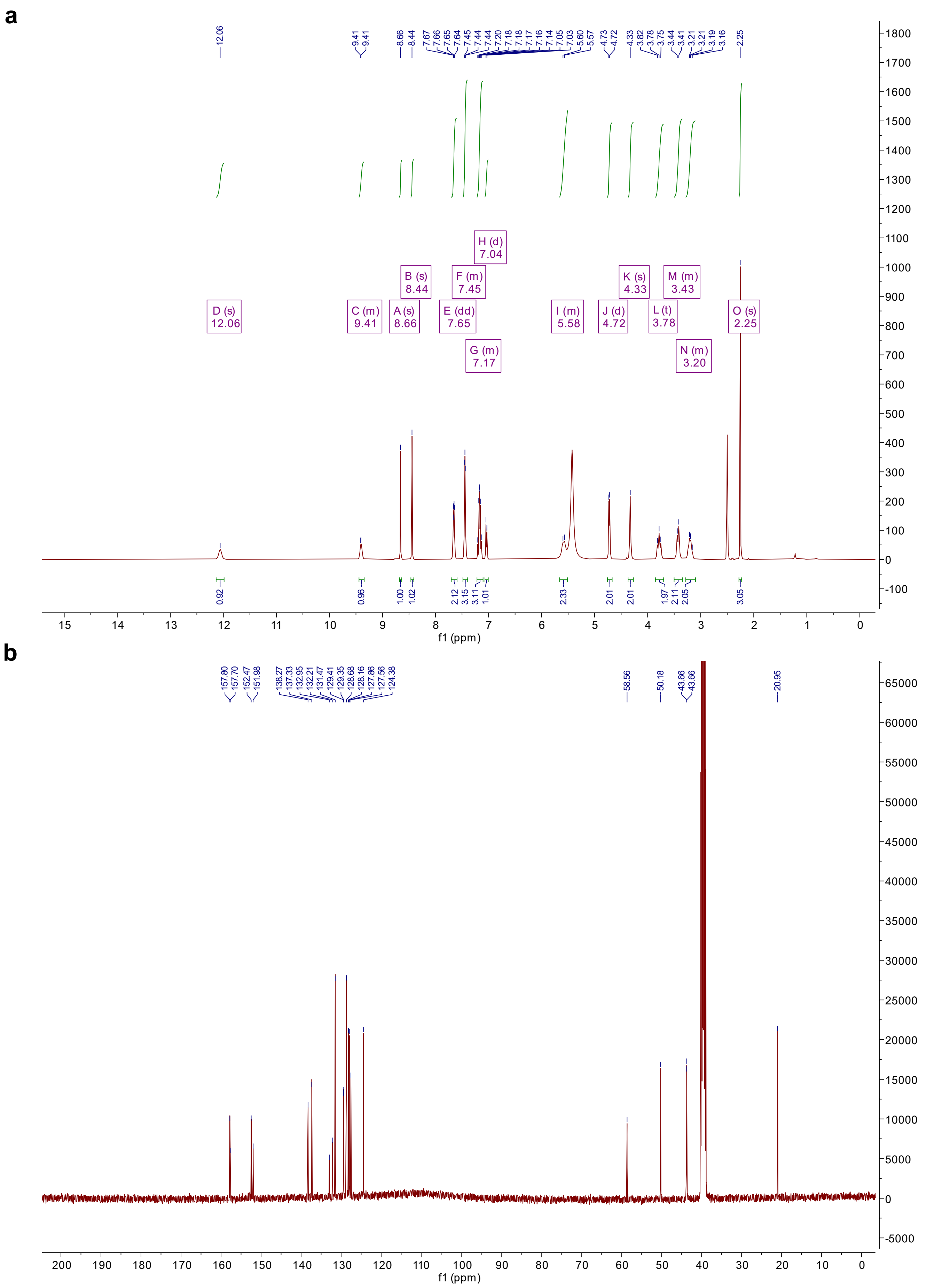

Supplementary Figure 23. NMR analyses of BI8626 derivative 2
(a) ^{1}H and **(b)** ^{13}C NMR spectrum of the synthesized derivative 2 as hydrochloride (see Table 1).

a

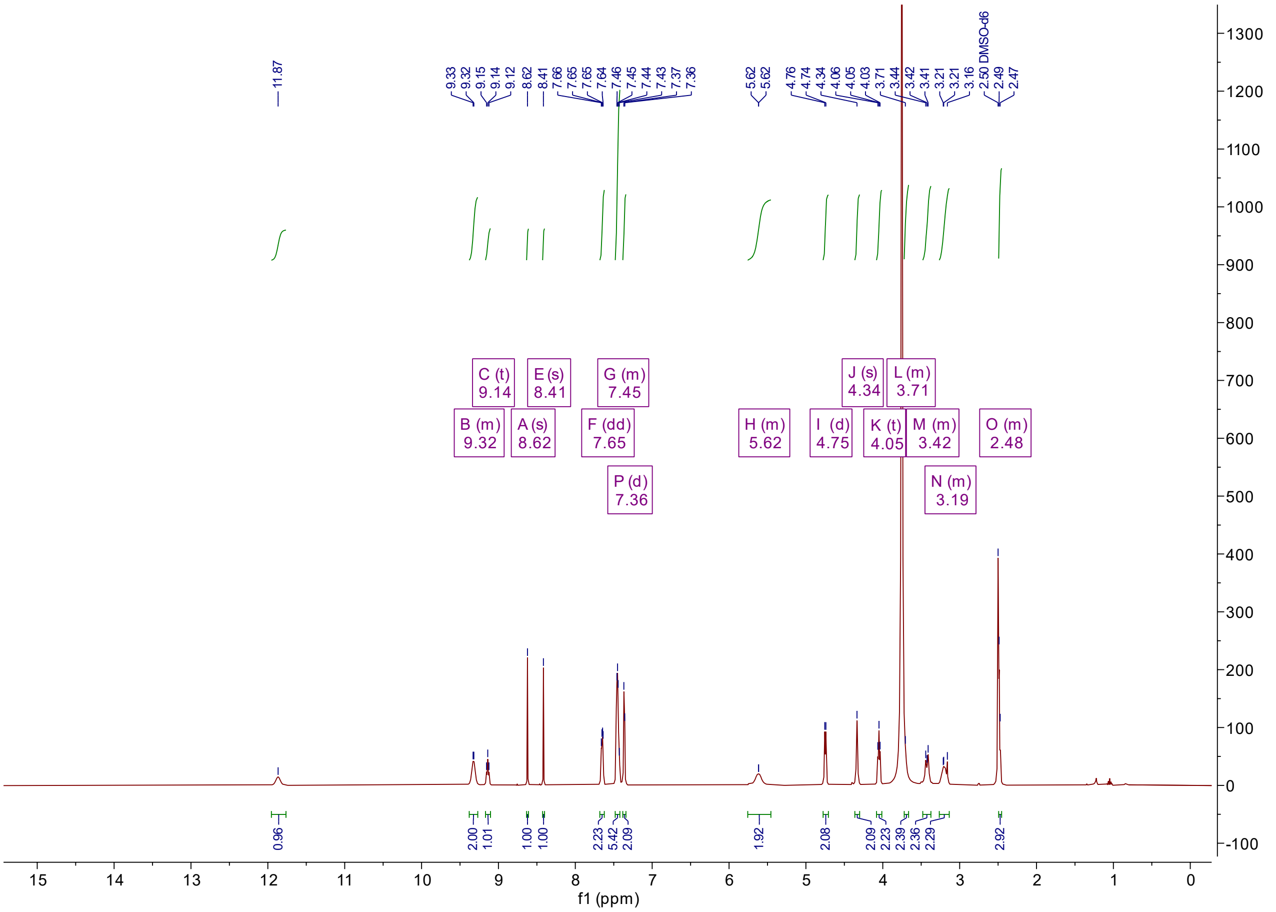

b

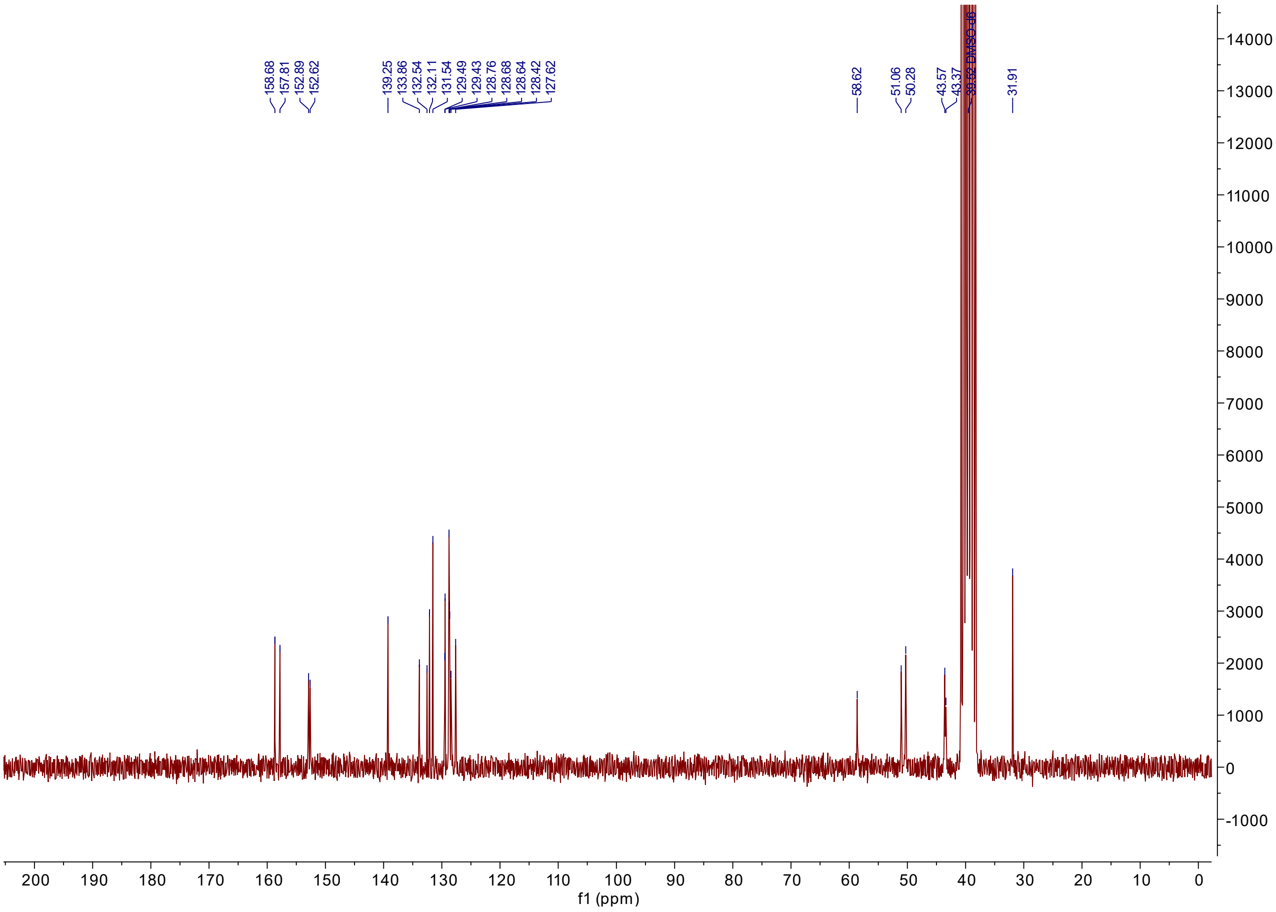

Supplementary Figure 24. NMR analyses of BI8626 derivative 3

(a) ^{1}H and **(b)** ^{13}C NMR spectrum of the synthesized derivative 3 as dihydrochloride (see Table 1).

a

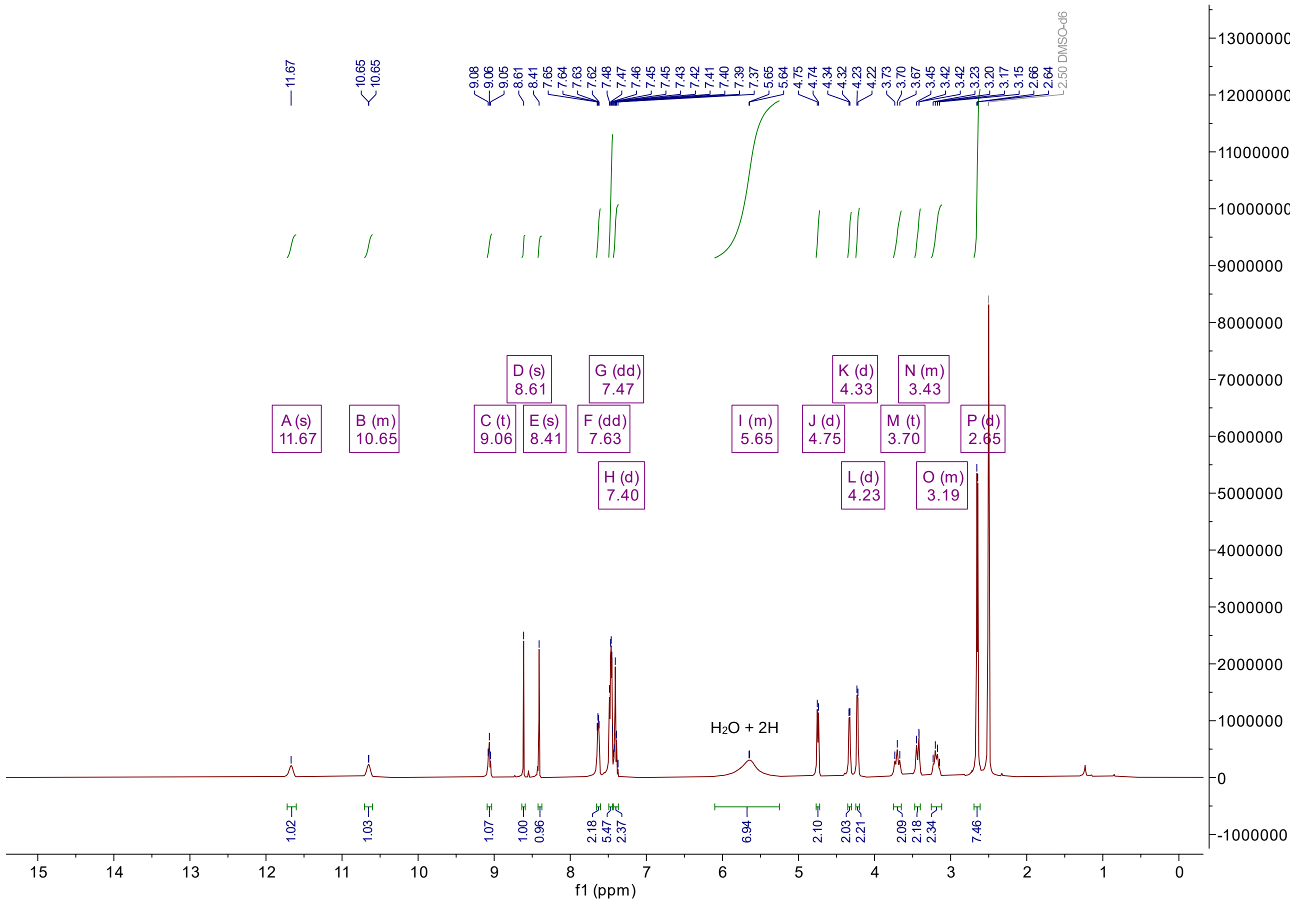

b

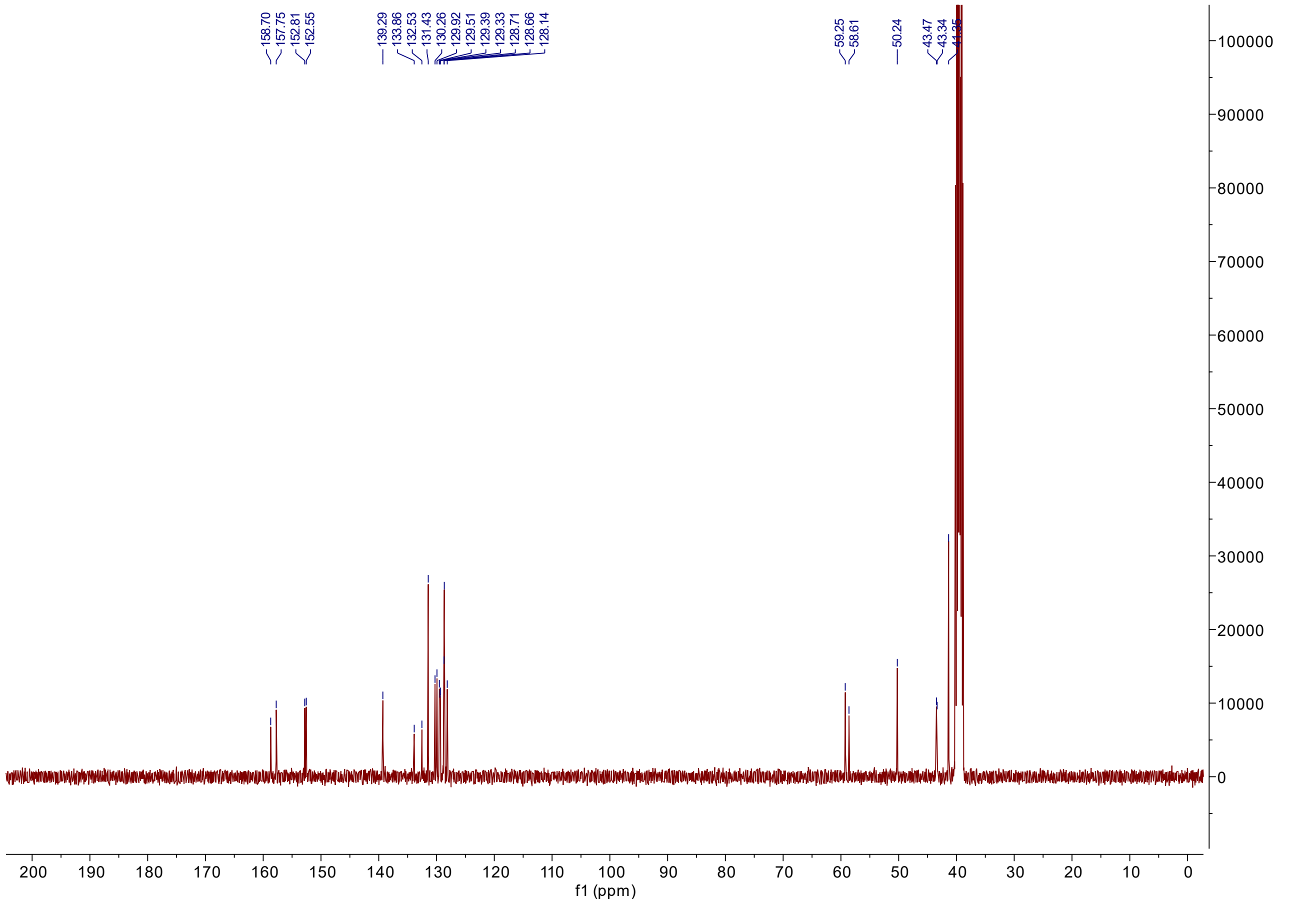

Supplementary Figure 25. NMR analyses of BI8626 derivative 4
(a) ^{1}H and **(b)** ^{13}C NMR spectrum of the synthesized derivative 4 as dihydrochloride (see Table 1).

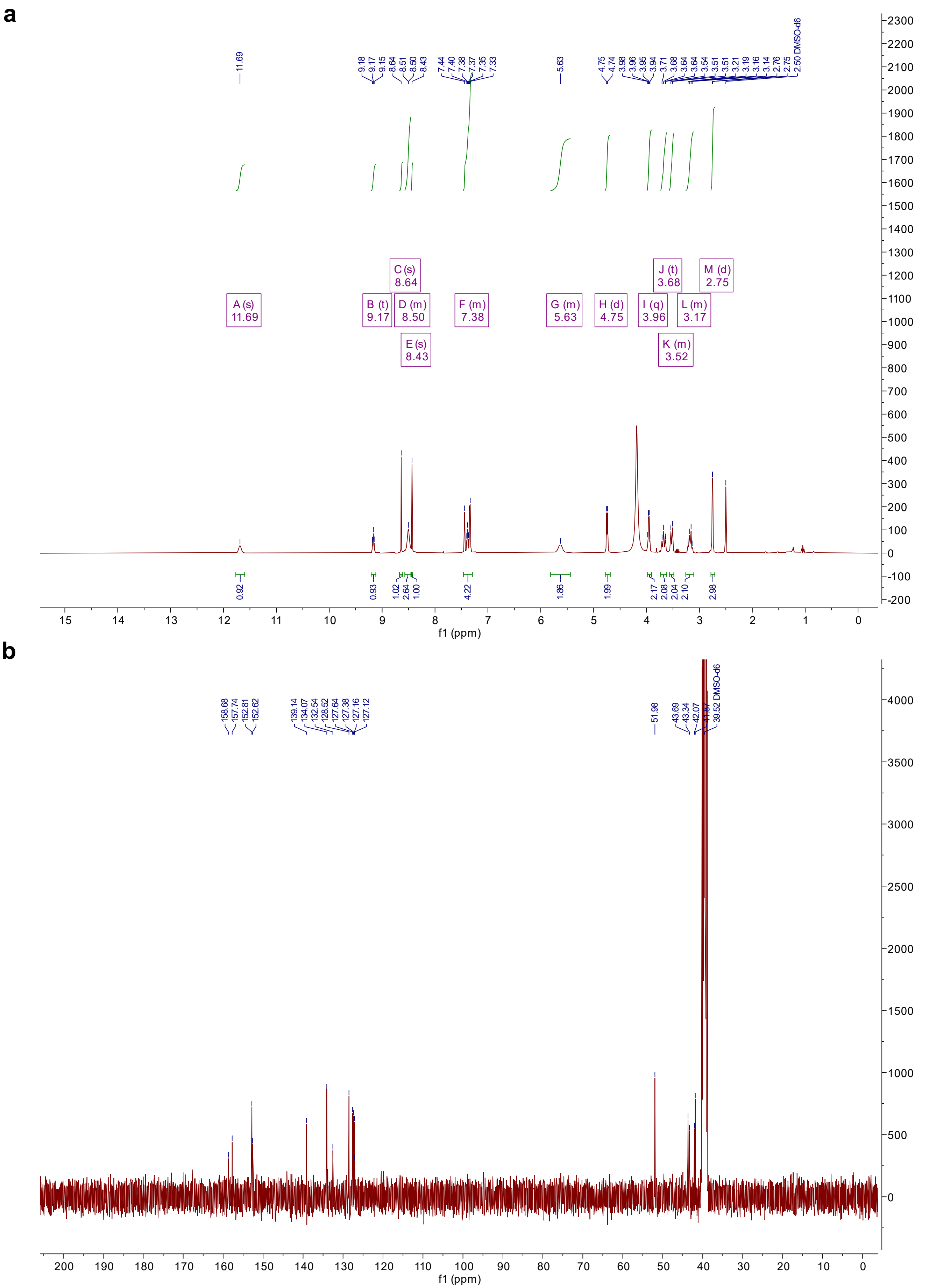

Supplementary Figure 26. NMR analyses of BI8626 derivative 5

(a) ^{1}H and **(b)** ^{13}C NMR spectrum of the synthesized derivative 5 as dihydrochloride (see Table 1).

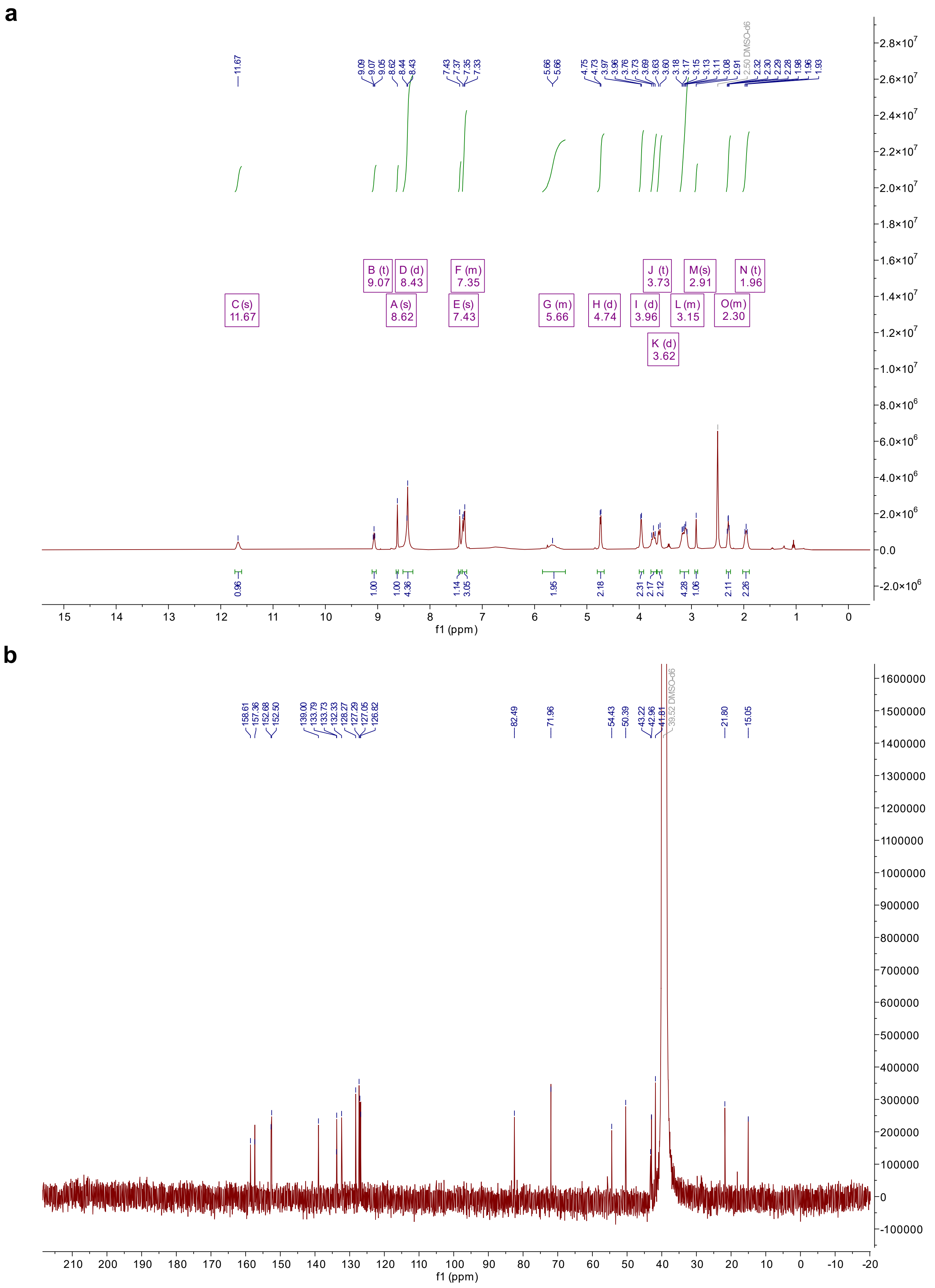

Supplementary Figure 27. NMR analyses of BI8626 derivative 6
(a) ^{1}H and **(b)** ^{13}C NMR spectrum of the synthesized derivative 6 as dihydrochloride (see Table 1).

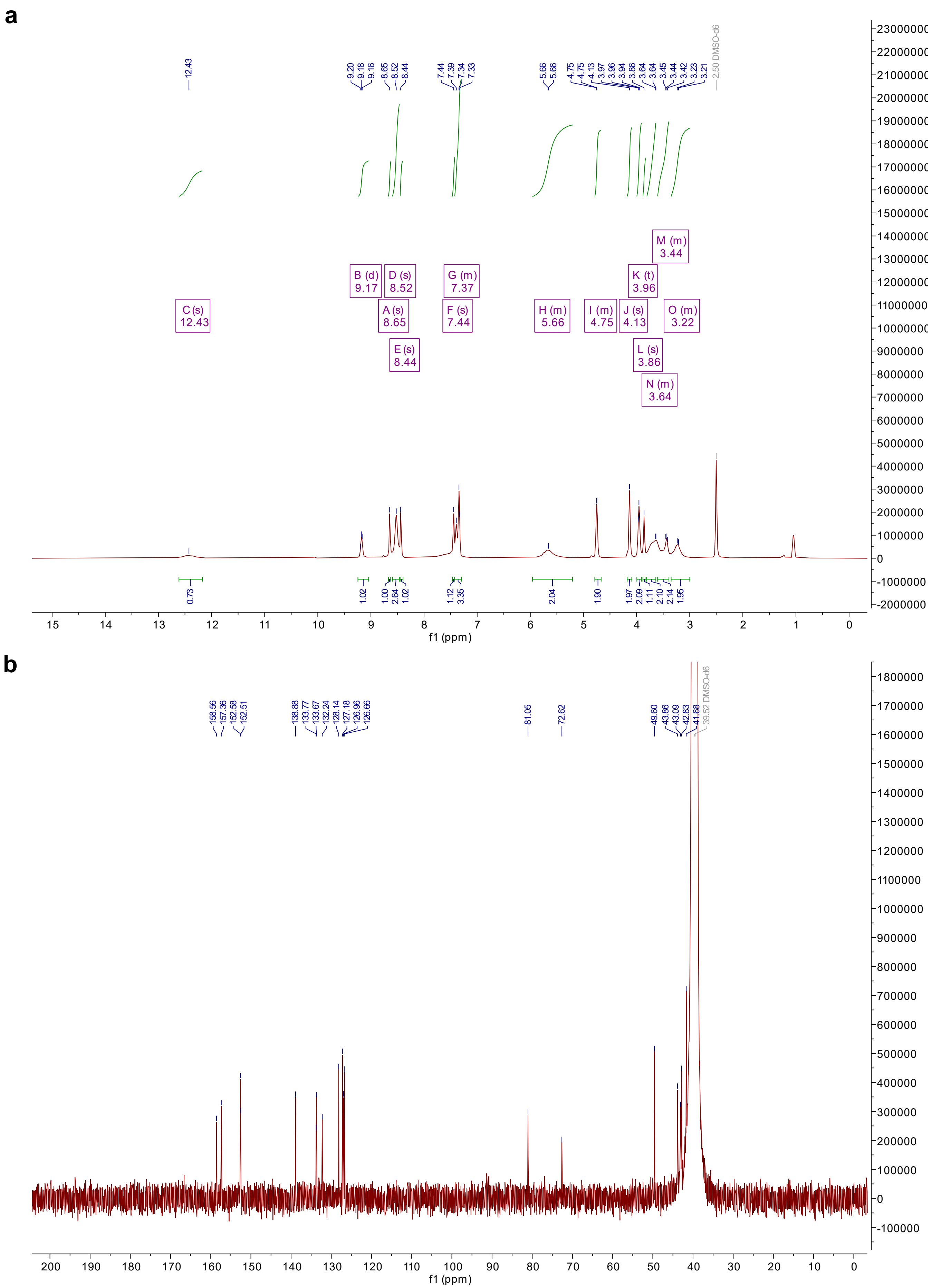

Supplementary Figure 28. NMR analyses of BI8626 derivative 8

(a) ^{1}H and **(b)** ^{13}C NMR spectrum of the synthesized derivative 8 as dihydrochloride (see Table 1).

SUPPLEMENTARY TABLES

		Monoisotopic mass						
	Composition+H	$[M+H]^+$	$[M+2H]^{2+}$	$[M+3H]^{3+}$	$[M+4H]^{4+}$	$[M+5H]^{5+}$	$[M+6H]^{6+}$	$[M+7H]^{7+}$
**Pep1 = GG**	$C_4H_9N_2O_3$		67.034571					
**Pep2 = LRGG**	$C_{16}H_{32}N_7O_5$		201.627159	134.754047	101.317492	81.255558		
**Pep3 = ESTLHLVLRLRGG**	$C_{63}H_{112}N_{21}O_{18}$			484.288357	363.468224			
**Pep4 = QLEDGRTLSDYNIQKESTLHLVLRLRGG**	$C_{138}H_{232}N_{43}O_{45}$				803.685556	643.15001	536.126312	
		Monoisotopic mass						
	[Composition-H_2O]+H	$[M+H]^+$	$[M+2H]^{2+}$	$[M+3H]^{3+}$	$[M+4H]^{4+}$	$[M+5H]^{5+}$	$[M+6H]^{6+}$	$[M+7H]^{7+}$
**Pep1+BI8622**	$C_{29}H_{33}N_8O_3$	541.267562	271.1376935	181.094404	136.0727593			
**Pep1+BI8626 or derivative 1**	$C_{29}H_{35}N_{10}O_2$	555.2944454	278.1511352	185.7700318	139.5794801			
**Pep2+BI8622**	$C_{41}H_{56}N_{13}O_5$	810.452737	405.730281	270.8227957	202.8651405	162.8968074		
**Pep2+BI8626 or derivative 1**	$C_{41}H_{58}N_{15}O_4$	824.4796204	412.7437227	275.4984235	206.8757739	165.7021841		
**Pep3+BI8622**	$C_{88}H_{136}O_{18}N_{27}$		930.031746	620.357105	465.519785	372.617393		
**Pep3+BI8626 or derivative 1**	$C_{88}H_{138}O_{17}N_{29}$		937.045188	625.032733	469.026506	375.42277		
**Pep4+BI8622**	$C_{163}H_{256}O_{45}N_{49}$			1207.313548	905.737117	724.791259	604.160686	517.995992
**Pep4+BI8626 or derivative 1**	$C_{163}H_{258}O_{44}N_{51}$			1211.989176	909.243838	727.596635	606.4985	519.999832

Supplementary Table 1. Targeted masses in MS/MS analyses

SUPPLEMENTARY METHODS

Molecular Dynamics (MD) simulations

Force field parameters: we used the Charmm-GUI to assign initial parameters from the CHARMM General Force Field.[1,2] All parameters not included in CGEFF were assigned analogously to existing parameters. The parameters were further optimized to reproduce energies calculated using quantum mechanics methods. Overall we followed the recommended protocol for optimizing small molecule parameters for the CHARMM force field;[1] all QM calculations were carried out in Psi4 v.1.9.1.[3] First, partial charges were optimized to reproduce interaction energies between the model compound and probe water molecules, calculated with HF/6-31g*. To this end the compound was split into two smaller fragments (Supplementary Fig. 11a), and a total of 12 interaction energies were calculated between the fragments and water molecules. Note that the 1,4-dimethylpiperazine fragment was treated as protonated. Subsequently we optimized the partial charges of the small molecules to reproduce the QM energies, using the monte carlo algorithm implemented in FFParam v.1.1 (MacKerell Lab, University of Maryland).[4] Following optimization, the root mean square error between the force field and quantum mechanics interaction energies was 0.66 kcal mol^{-1}. Thereafter, we optimized the bonded parameters. As in the previous step, the molecule was broken down into smaller fragments (Supplementary Fig. 11b). To fit the missing dihedral parameters, we first generated a series of conformers by incrementing the dihedral angle by 5 ° between -180° and +180° for freely rotatable groups and -80° to +80° for dihedral angles in non-aromatic ring systems. Subsequently, we optimized the geometry of each conformer, freezing the dihedral angle that was incremented at its current value. Geometry was optimized using second-order Møller–Plesset perturbation theory (MP2) with the 6-311g* basis set. The energy of the optimized conformer was used as a reference for subsequent parameter optimization. Because this approach can be sensitive to the starting configuration, we used the wavefront propagation method implemented in torsiondrive to generate the reference energies.[5] Subsequently we used force balance to fit the force field parameters to the reference energies.[6] The optimized, force field-based energies showed good agreement with the reference energies calculated by quantum mechanics (Supplementary Fig. 11c). Finally the fragment parameters were combined and the final parameters of BI8626 were compiled in gromacs format.

Simulation setup**:** to investigate the possible binding sites of BI8626 in the vicinity of Gly76 of the $HUWE1^{HECT}$-linked donor Ub, we performed explicit-solvent all-atom MD simulations, starting from a crystal structure (PDB: 6XZ1[7]). The vinyl thioether linkage between Gly76 of Ub and Cys4341 of HUWE1 was replaced by the native thioester. All crystal water positions were maintained for the starting structure, which was placed into a dodecahedron box with a minimum distance of 1.5 nm between the atoms and the box boundaries. The system was solvated with tip3p water[8], using the 'solvate' module of the GROMACS package[9]. GENION[9] was used to add 150 mM NaCl and neutralize with Na^+ ions, resulting in a system size of 113,443 atoms. All simulations were performed using GROMACS v.2022.6 (GROMACS Development Team)[9] with the charmm36 force field[10]. Lennard–Jones and short-range electrostatic interactions were computed within a cut-off of 1.2 nm. Long-range electrostatic interactions were computed for distances larger than 1.2 nm, using the particle-mesh Ewald summation[11] with 0.12 nm grid spacing.

Bond lengths were constrained using the LINCS algorithm[12] and a 2 fs integration time step was used. Temperature coupling was performed using v-rescale[13] with a 0.1 ps coupling time constant. Solute and solvent atoms were coupled independently to a heat bath of 300 K. The pressure was coupled to a stochastic cell rescaling barostat[14] with a time constant of 5 ps and scaling the box every 10 steps.

To obtain BI8626 starting structures for the simulations, we first selected a random position located on a 20 Å sphere centered at the position of the carbonyl C atom of Gly76 of ubiquitin. Next, we used the program insert-molecules to place BI8626 with a random orientation at that position. If the BI8626 atoms did not overlap with HUWE1^{HECT} and Ub atoms, the starting structure was accepted and a new position was selected. Otherwise, the orientation was discarded and a new orientation tested. After 100 unsuccessful orientation trials, the position was discarded and a new random position was selected. This process was repeated until 100 starting structures were obtained. For each of these, the system was first energy minimized with harmonic position restraints ($k = 1{,}000$ kJ mol^{-1} nm^{-1}) applied to the solute heavy atoms. To allow for solvent equilibration, the system was simulated with position restraints for 20 ns, followed by 2 µs of production simulations without position restraints. To focus the sampling of BI8626 on a region close to Gly76, an additional flat-bottom potential was applied which varied with the distance between the carbonyl-C of Gly76 and the center of mass of BI8626 as follows. The potential is 0 kJ between 0 nm and 3 nm distance and then rises harmonically with 1,000 kJ mol^{-1} nm^{-1}. Coordinates were recorded every 10 ps and only the production trajectories were used for further analyses.

Analyses: To characterize possible binding poses of BI8626, we first filtered the trajectories by extracting frames with a Gly76 (C) - BI8626 (primary amino nitrogen) distance below 4 Å (Supplementary Fig. 11d). As a second filter, we calculated the Bürgi-Dunitz angle of the nucleophilic attack on the carbonyl of Gly76 of Ub and accepted poses within a range of 109.5°±45° (Supplementary Fig. 11e). For all of the accepted poses, we calculated contact occupancies and carried out a principal component analysis (PCA; Supplementary Fig. 11f). Here, a contact between BI8626 and a HUWE1^{HECT} or Ub residue was defined as a distance within 5 Å (minimal pair-wise distance between ligand and residue atoms). The occupancy is the number of frames with a contact divided by the number of frames in a trajectory. For the PCA, the accepted poses were first aligned by rigid-body fitting using the coordinates of the backbone atoms of all residues that are located within 8 Å of the C atom of Gly76 of the donor Ub in the crystal structure. Then the coordinates of all heavy atoms of BI8626, except for the phenyl group, were concatenated and the atomic displacement covariance matrix was calculated. A histogram of the projections of all poses onto the first to principal components (PCs, i.e. eigenvectors of the covariance matrix) was generated (Supplementary Fig. 11f). To extract conformations from the maxima of the histogram, we calculated for each bin position the number of frames within 1 Å on the plane spanned by PC1 and PC2. The bin position with the highest number of frames was considered as the first maximum. After removing the frames attributed to the first maximum, we repeated this procedure, until 4 maxima were obtained. For each maximum, we extracted the frame of which the Bürgi-Dunitz angle was closest to 109.5° (Supplementary Fig. 11g).

AlphaFold3 (AF3)-based structure predictions

The tetrahedral intermediate of the HUWE1^{HECT}-Ub-BI8626 complex was predicted with AlphaFold v.3.0.1 (https://github.com/google-deepmind/alphafold3)[15]. Ub was linked to the active-site cysteine, Cys4341, of HUWE1^{HECT} by truncating Gly76 of Ub and providing it as a free ligand, subsequently defining bonds between the g-S of Cys4341 and the carbonyl-C of Gly76 of Ub and between the primary amino nitrogen of Gly76 and the carbonyl-C of Gly75 of Ub, and between the primary amino nitrogen of BI8626 and the carbonyl-C of Gly76 of Ub.

SUPPLEMENTARY REFERENCES

1. Vanommeslaeghe, K. *et al.* CHARMM general force field: A force field for drug-like molecules compatible with the CHARMM all-atom additive biological force fields. *J. Comput. Chem.* **31**, 671–690 (2010).

2. Jo, S., Kim, T., Iyer, V. G. & Im, W. CHARMM-GUI: A web-based graphical user interface for CHARMM. *J. Comput. Chem.* **29**, 1859–1865 (2008).

3. Smith, D. G. A. *et al.* PSI4 1.4: Open-source software for high-throughput quantum chemistry. *J. Chem. Phys.* **152**, 184108 (2020).

4. Kumar, A., Yoluk, O. & MacKerell, A. D. FFParam: Standalone package for CHARMM additive and Drude polarizable force field parametrization of small molecules. *J. Comput. Chem.* **41**, 958–970 (2020).

5. Qiu, Y. *et al.* Driving torsion scans with wavefront propagation. *J. Chem. Phys.* **152**, 244116 (2020).

6. Wang, L.-P., Martinez, T. J. & Pande, V. S. Building Force Fields: An Automatic, Systematic, and Reproducible Approach. *J. Phys. Chem. Lett.* **5**, 1885–1891 (2014).

7. Nair, R. M. *et al.* Reconstitution and Structural Analysis of a HECT Ligase-Ubiquitin Complex via an Activity-Based Probe. *ACS Chem. Biol.* **16**, 1615–1621 (2021).

8. Jorgensen, W. L., Chandrasekhar, J., Madura, J. D., Impey, R. W. & Klein, M. L. Comparison of simple potential functions for simulating liquid water. *The Journal of Chemical Physics* **79**, 926–935 (1998).

9. Abraham, M. J. *et al.* GROMACS: High performance molecular simulations through multi-level parallelism from laptops to supercomputers. *SoftwareX* **1**, 19–25 (2015).

10. Huang, J. & MacKerell, A. D. CHARMM36 all-atom additive protein force field: Validation based on comparison to NMR data. *J. Comput. Chem.* **34**, 2135–2145 (2013).

11. Essmann, U. *et al.* A smooth particle mesh Ewald method. *J. Chem. Phys.* **103**, 8577–8593 (1995).

12. Hess, B. P-LINCS: A Parallel Linear Constraint Solver for Molecular Simulation. *J. Chem. Theory Comput.* **4**, 116–122 (2008).

13. Bussi, G., Donadio, D. & Parrinello, M. Canonical sampling through velocity rescaling. *J. Chem. Phys.* **126**, 014101 (2007).

14. Bernetti, M. & Bussi, G. Pressure control using stochastic cell rescaling. *J. Chem. Phys.* **153**, 114107 (2020).

15. Abramson, J. *et al.* Accurate structure prediction of biomolecular interactions with AlphaFold 3. *Nature* **630**, 493–500 (2024).
