# Supplementary material for: Selective ubiquitination of drug-like small molecules by the ubiquitin ligase HUWE1
Source: Nat Commun. 2025 Sep 2;16:8182. doi: 10.1038/s41467-025-63442-x (PMC12405493; doi:10.1038/s41467-025-63442-x)
Supplement: Supplementary file 2 — Description of Additional Supplementary Files [file 41467_2025_63442_MOESM2_ESM.pdf]

Description of Additional Supplementary Files

File Name: Supplementary Data 1

Description: HDX-MS data for the HUWE1^{HECT}~Ub proxy and HUWE1^{HECT} in the presence of BI8626, BI8622, and DMSO (as a control), respectively.

File Name: Supplementary Data 2

Description: global quantitative and diGly proteomic data from HEK293 cells upon BI8626 treatment (15 µM; 24h) compared to a DMSO control.
