# Supplementary material for: Selective ubiquitination of drug-like small molecules by the ubiquitin ligase HUWE1
Source: Nat Commun. 2025 Sep 2;16:8182. doi: 10.1038/s41467-025-63442-x (PMC12405493; doi:10.1038/s41467-025-63442-x)
Supplement: Supplementary file 5 — Reporting Summary [file 41467_2025_63442_MOESM5_ESM.pdf]

nature portfolio

Corresponding author(s): Sonja Lorenz

Last updated by author(s): 19/8/25

Reporting Summary

Nature Portfolio wishes to improve the reproducibility of the work that we publish. This form provides structure for consistency and transparency in reporting. For further information on Nature Portfolio policies, see our Editorial Policies and the Editorial Policy Checklist.

Statistics

For all statistical analyses, confirm that the following items are present in the figure legend, table legend, main text, or Methods section.

n/a	Confirmed	
☐	☒	The exact sample size (*n*) for each experimental group/condition, given as a discrete number and unit of measurement
☐	☒	A statement on whether measurements were taken from distinct samples or whether the same sample was measured repeatedly
☐	☒	The statistical test(s) used AND whether they are one- or two-sided<br>*Only common tests should be described solely by name; describe more complex techniques in the Methods section.*
☒	☐	A description of all covariates tested
☒	☐	A description of any assumptions or corrections, such as tests of normality and adjustment for multiple comparisons
☐	☒	A full description of the statistical parameters including central tendency (e.g. means) or other basic estimates (e.g. regression coefficient) AND variation (e.g. standard deviation) or associated estimates of uncertainty (e.g. confidence intervals)
☐	☒	For null hypothesis testing, the test statistic (e.g. *F*, *t*, *r*) with confidence intervals, effect sizes, degrees of freedom and *P* value noted<br>*Give P values as exact values whenever suitable.*
☒	☐	For Bayesian analysis, information on the choice of priors and Markov chain Monte Carlo settings
☒	☐	For hierarchical and complex designs, identification of the appropriate level for tests and full reporting of outcomes
☒	☐	Estimates of effect sizes (e.g. Cohen's *d*, Pearson's *r*), indicating how they were calculated

Our web collection on statistics for biologists contains articles on many of the points above.

Software and code

Policy information about availability of computer code

Data collection	CFX Maestro version 1.1 (Bio-Rad), Unicorn version 7.9.02440 (Cytiva), Xcalibur version 2.4 (Thermo Fisher Scientific), ITC200 version 1.26.1 (GE Healthcare), ImageStudio Lite version 5.2 (LI-COR), Azure Biosystems version 1.5.0.0518 (Azure Biosystems), Image Reader LAS-1000 Pro version 2.6 (Fuji Photo Film Co.), EPSON scan version 3.9.3.4 (EPSON), Tune version 4.0.4084.22 (Thermo Scientific)
Data analysis	Prism version 9.3.1 (471) (GraphPad), PyMOL version 2.5.0 (Schrödinger), ImageStudio Lite version 5.2 (LI-COR), OriginPro 2020b (OriginLab), Freestyle version 1.9 (Thermo Fisher Scientific), MaxQuant version 2.0.3.0 (Max Planck Institute of Biochemistry), ProteinLynx Global SERVER (PLGS) version 3.0.1 (Waters), DynamX version 3.0 (Waters), MSConvert (ProteoWizard Project), AlphaFold version 3.0.1 (DeepMind), GROMACS version 2022.6 (GROMACS Development Team), Python version 3.9.13 (Python Software Foundation), Psi4 version 1.9.1 (Psi4 Developer Community), FFParam version 1.1 (MacKerell Lab, University of Maryland), MestReNova version 14.2.0-26256 (Mestrelab Research), Unicorn version 7.9.02440 (Cytiva), Perseus version 1.6.14.0 (Max Planck Institute of Biochemistry)

For manuscripts utilizing custom algorithms or software that are central to the research but not yet described in published literature, software must be made available to editors and reviewers. We strongly encourage code deposition in a community repository (e.g. GitHub). See the Nature Portfolio guidelines for submitting code & software for further information.

Data

Policy information about availability of data

All manuscripts must include a data availability statement. This statement should provide the following information, where applicable:

- Accession codes, unique identifiers, or web links for publicly available datasets
- A description of any restrictions on data availability
- For clinical datasets or third party data, please ensure that the statement adheres to our policy

The HDX-MS data are available in Supplementary Data 1, global and diGly proteomic data in Supplementary Data 2. Additionally, the HDX-MS data (accession code: PXD066563), the global and diGly proteomic data (accession code: PXD067088), and other MS data (accession code: PXD063550) have been uploaded to the ProteomeXchange Consortium via the PRIDE partner repository.70 The MD force field parameters for BI8626, MD-derived models and the AF3-based dataset have been deposited to Zenodo (DOI: 10.5281/zenodo.15772692). Source data are provided with this paper.

Research involving human participants, their data, or biological material

Policy information about studies with human participants or human data. See also policy information about sex, gender (identity/presentation), and sexual orientation and race, ethnicity and racism.

Reporting on sex and gender	not applicable
Reporting on race, ethnicity, or other socially relevant groupings	not applicable
Population characteristics	not applicable
Recruitment	not applicable
Ethics oversight	not applicable

Note that full information on the approval of the study protocol must also be provided in the manuscript.

Field-specific reporting

Please select the one below that is the best fit for your research. If you are not sure, read the appropriate sections before making your selection.

☒ Life sciences ☐ Behavioural & social sciences ☐ Ecological, evolutionary & environmental sciences

For a reference copy of the document with all sections, see nature.com/documents/nr-reporting-summary-flat.pdf

Life sciences study design

All studies must disclose on these points even when the disclosure is negative.

Sample size	The data presented in the manuscript represent the averages and/or representatives of at least 3 independent replicates (see section "statistics and reproducibility") . These sample sizes were chosen to generate data at sufficient depth and assess differences between conditions robustly. These sample sizes are sufficient, since the observed effects of interest are clearly detectable between conditions and robust across replicates.
Data exclusions	No data were excluded.
Replication	All experiments were performed for at least n=3 independent samples, as described, and all attempts were successful. Immunoblots and enzyme assays were performed independently 3 times with similar results. Biochemical in-vitro experimets and functional cell-based assays were performed on separate and fully independent occassions and verified each other.
Randomization	Gel-based samples were run in different orders with the same result.
Blinding	MS data were analyzed with script-based pipelines, in which results are largely independent from interference of the researchers. The precise workflows are detailed in the methods section. Gel-based assays were replicated by different individuals. The investigators were not blinded, which is standard in this type of study due to multiple steps that require precise operations for accuracy and precision.

Reporting for specific materials, systems and methods

We require information from authors about some types of materials, experimental systems and methods used in many studies. Here, indicate whether each material, system or method listed is relevant to your study. If you are not sure if a list item applies to your research, read the appropriate section before selecting a response.

Materials & experimental systems

n/a	Involved in the study
☐	☒ Antibodies
☐	☒ Eukaryotic cell lines
☒	☐ Palaeontology and archaeology
☒	☐ Animals and other organisms
☒	☐ Clinical data
☒	☐ Dual use research of concern
☒	☐ Plants

Methods

n/a	Involved in the study
☒	☐ ChIP-seq
☒	☐ Flow cytometry
☒	☐ MRI-based neuroimaging

Antibodies

Antibodies used

The following primary antibodies were used: anti-Actin mouse monoclonal antibody (A1978, Sigma-Aldrich; dilution 1:1,000); anti-HECTH9 AX8D1 mouse monoclonal antibody (5695, Cell Signaling Technology; dilution 1:1,000), anti-MCL1 D2W9E (94296, Cell Signaling Technology; dilution 1:1,000), anti-HA-HRP mouse monoclonal antibody (H6533, Sigma-Aldrich; dilution 1:4000), and the K-ε-GG kit 5562, Cell Signaling Technology (for IP-MS). Secondary antibodies for luminescence-based detection included goat anti-rabbit HRP-linked antibody 7074 or goat anti-mouse HRP-linked antibody 7076 (both Cell Signaling Technology; dilution 1:10,000).

Validation

Validation was performed by the manufacturer, as follows: A1978 (Sigma-Aldrich): WB, IHC, IF; 5695 (Cell Signaling Technology): WB; 94296 (Cell Signaling Technology): WB, IP, IF, F; H6533 (Sigma-Aldrich): WB; 7074 (Cell Signaling Technology): WB; 7076 (Cell Signaling Technology): WB.

Eukaryotic cell lines

Policy information about cell lines and Sex and Gender in Research

Cell line source(s)

Commercial HEK293T cells (ATCC)

Authentication

Cell lines were not authenticated beyond monitoring the morphology and growth rates continuously, and PCR-amplifying the CRISPR/Cas9-mediated deletion site from genomic DNA extracted from the cells to confirm the HUWE1 KO.

Mycoplasma contamination

All cell lines were tested negative for mycoplasma contamination.

Commonly misidentified lines
(See ICLAC register)

No commonly misidentified lines were used.

Plants

Seed stocks

not applicable

Novel plant genotypes

not applicable

Authentication

not applicable
